# The *ben1-1* Brassinosteroid-Catabolism Mutation Is Unstable Due to Epigenetic Modifications of the Intronic T-DNA Insertion

**DOI:** 10.1534/g3.113.006353

**Published:** 2013-09-01

**Authors:** Kulbir Singh Sandhu, Pushpa Sharma Koirala, Michael M. Neff

**Affiliations:** Department of Crop and Soil Sciences, Washington State University, Pullman, Washington 99164

## Abstract

Loss-of-function genetic analysis plays a pivotal role in elucidating individual gene function as well as interactions among gene networks. The ease of gene tagging and cloning provided by transfer DNA (T-DNA) insertion mutants have led to their heavy use by the *Arabidopsis* research community. However, certain aspects of T-DNA alleles require caution, as highlighted in this study of an intronic insertion mutant (*ben1-1*) in the *BEN1* (*BRI1-5 ENHANCED 1*) gene. As a part of our analysis of brassinosteroid catabolic enzymes, we generated a genetic triple-mutant from a cross between the *bas1-2 sob7-1* double-null (T-DNA exonic insertion mutants of *phyB-4 ACTIVATION TAGGED SUPPRESSOR 1* and *SUPPRESSOR OF phyB-4 7*) and *ben1-1*. As previously described, the single *ben1-1* line behaves as a transcript null. However, in the triple-mutant background *ben1-1* was reverted to a partial loss-of-function allele showing enhanced levels of the wild-type-spliced transcript. Interestingly, the enhanced expression of *BEN1* remained stable when the *ben1-1* single-mutant was reisolated from a cross with the wild type. In addition, the two genetically identical pretriple and posttriple *ben1-1* mutants also differed phenotypically. The previously functional *NPTII* (*NEOMYCIN PHOSPHOTRANSFERASE II*) T-DNA marker gene (which encodes kanamycin resistance) was no longer functional in the recovered *ben1-1* allele, though the length of the T-DNA insertion and the *NPTII* gene sequence did not change in the pretriple and posttriple *ben1-1* mutants. Methylation analysis using both restriction endonuclease activity and bisulfite conversion followed by sequencing showed that the methylation status of the T-DNA is different between the original and the recovered *ben1-1*. These observations demonstrate that the recovered *ben1-1* mutant is epigenetically different from the original *ben1-1* allele.

Loss-of-function mutants play an essential role in genetic analysis. Mutation-tagging approaches, such as mutagenesis by transfer DNA (T-DNA) insertion, takes advantage of *Agrobacterium*-mediated plant transformation where a modified Ti plasmid is integrated into the plant genome (Krysan *et al.* 1999). In plant species that are amenable to transformation by *Agrobacterium*, community projects have been undertaken to generate large collections of genomic T-DNA insertion libraries (Sessions *et al.* 2002; Alonso *et al.* 2003; An *et al.* 2005; Thole *et al.* 2011). The ease of gene tagging and cloning provided by T-DNA insertion mutants has led to their extensive use in *Arabidopsis* research.

Mutagenic T-DNAs could be inserted into any part of a gene, leading to an array of outcomes (Krysan *et al.* 1999). Exonic T-DNA insertions may create null-alleles due to the introduction of a premature stop codon or a frame-shift mutation. Intronic T-DNA insertions may generate null-alleles due to failure of proper splicing, leading to an aberrant/truncated protein (Wang 2008). However, in some cases intronic T-DNA insertions generate knock-down alleles due to RNA/RNA interaction between the extended RNA (transcribed from the gene including the inserted T-DNA) and an RNA transcribed from a part of the complementary T-DNA strand (Gao and Zhao 2012). Existence of this mechanism is supported by the observation that intronic T-DNA insertion alleles are susceptible to instability because of trans T-DNA interactions which disrupt or inhibit the RNA/RNA duplex (Gao and Zhao 2012). The trans T-DNA interactions are known to be commonly associated with DNA methylation, which might play a role in the mechanism of locus instability. For example, the instability of the intronic SALK T-DNA insertion mutation in the gene essential for cellulose synthesis, *COBRA* (*cob-6*), is shown to be dependent upon the activity of enzymes involved in DNA methylation (Xue *et al.* 2012).

These complications associated with T-DNA-insertion alleles require caution, as highlighted in this study of an intronic insertion mutant (*ben1-1*) in the *BEN1* (*BRI1-5 ENHANCED 1*) gene. The *BEN1* overexpressor mutant *BRI1-5 ENHANCED1-1DOMINANT* (*ben1-1D*) has a characteristic BR-deficient phenotype (Yuan *et al.* 2007). A transcript-null mutant *ben1-1* with a T-DNA insertion in the second intron of *BEN1* has elevated BR levels compared with the wild type. Although the mechanism of BEN1 activity is not known, genetic data support the hypothesis that *BEN1* is involved in BR inactivation. *BEN1* expression is up-regulated in seedlings grown in white light compared with those grown in the dark. *ben1-1* seedlings are also less responsive to light-mediated inhibition of hypocotyl growth, suggesting a role in seedling photomorphogenesis (Yuan *et al.* 2007).

BR inactivation also involves members of the cytochrome P450 gene family, *BAS1* (*phyB-4 ACTIVATION TAGGED SUPPRESSOR 1*) and *SOB7* (*SUPPRESSOR OF phyB-4 7*) (Neff *et al.* 1999; Turk *et al.* 2003; Turk *et al.* 2005). Overexpression of either *BAS1/CYP72B1/CYP734A1* or *SOB7/CYP72C1* suppresses the long-hypocotyl phenotype of *phyB-4* and also confers a BR-deficient phenotype (Neff *et al.* 1999; Turk *et al.* 2003; Turk *et al.* 2005). *BAS1* transcript accumulation is strongly feedback regulated in positive manner by BR levels (Tanaka *et al.* 2005). BR levels are increased in the *bas1-2 sob7-1* double-null mutant (first-exon T-DNA insertion alleles) compared with the wild type or either single null allele. *BAS1* and *SOB7* also affect developmental processes, such as hypocotyl elongation and flowering, in a synergistic/redundant fashion (Turk *et al.* 2005). Independent evolution of multiple BR-inactivating pathways indicates the importance of this process in plant growth and development. Therefore, identifying the contributions of enzymes and pathways related to the inactivation of these hormones is important for understanding BR-mediated development (Sandhu *et al.* 2012).

As a part of our analysis of BR catabolic enzymes, we generated a genetic triple-mutant from a cross between the *bas1-2 sob7-1* double-null and *ben1-1*. Our results show that the full loss-of-function *ben1-1* mutation was transformed to a partial loss-of-function mutation in the *bas1-2 sob7-1 ben1-1* (triple-mutant) background showing enhanced levels of the wild-type−spliced transcript. Interestingly, the enhanced expression of *BEN1* remained stable when the *ben1-1* single-mutant was reisolated from a cross with the wild-type. In addition, the *ben1-1* single-mutant isolated back from the triple mutant was phenotypically different than the original *ben1-1* allele in terms of seedling-development. The size of T-DNA insertion and the *NPTII* (*NEOMYCIN PHOSPHOTRANSFERASE II*) gene sequence did not change in the pretriple and posttriple *ben1-1* mutants. However, the previously functional *NPTII* T-DNA marker gene (which encodes kanamycin resistance) was no longer functional in the recovered *ben1-1* allele. Methylation analysis using both restriction endonuclease activity with methylation sensitive enzymes and bisulfite conversion followed by sequencing showed that the methylation status of the T-DNA is different between the original and the recovered *ben1-1*. Our study shows that the *ben1-1* BR-catabolism mutation is unstable due to epigenetic modifications of the intronic T-DNA insertion.

## Materials and Methods

### Plant material

All mutants used in this study, *ben1-1* (Yuan *et al.* 2007), *bas1-2*, and *sob7-1* (Turk *et al.* 2005), were in the Columbia (Col-0) background. *ben1-1* was crossed with *bas1-2 sob7-1*, and multiple mutant combinations were isolated from populations of F2 individuals derived from self-pollinated F1 plants. The *ben1-1* allele was characterized by amplifying genomic DNA with gene specific polymerase chain reaction (PCR) primers GSP1 and GSP2, and T-DNA−specific PCR primers LBb1.3 and PRT2 (Supporting Information, Table S1). Molecular-genetic analysis of the *bas1-2* and *sob7-1* alleles is described in Turk *et al.* (2005).

*ben1-1* was isolated from the triple-mutant *bas1-2 sob7-1 ben1-1* background by crossing with the wild-type (Col-0) and allowing the F1 population to self-pollinate. The subsequent F2 population was screened with PCR markers specific for the *bas1-2*, *sob7-1*, and *ben1-1* T-DNA insertions. An individual F2 plant homozygous for wild-type *SOB7* allele and heterozygous for *bas1-2* and *ben1-1* T-DNA insertions was thus identified. The F3 progeny of this individual was further screened to identify and recover multiple single *ben1-1* F3 lines (#11.4 and #11.5) as well as a *bas1-2 sob7-1 ben1-1* triple-mutant line. The homozygous F4 seeds derived from the identified individual F3 lines were used for quantitative RT-PCR and hypocotyl growth analysis.

### Exogenous hormone treatment

The stock solution of brassinolide (BL) was dissolved in 95% ethanol (v/v). BL treatment was given by adding the BL stock solution to the seedling growth media to a final concentration of 100 nM. Equal amounts of 95% ethanol were added to the negative-hormone control media.

### Hypocotyl measurement

Procedures for seed sterilization, plating, growth conditions and hypocotyl measurement were done as described in Turk *et al.* (2003).

### Transcript analysis

Total RNA was isolated, by the use of the RNeasy Plant Kit (QIAGEN, Valencia, CA), from 5-d-old seedlings grown in continuous white light (25 µmol m^−2^ sec^—1^). On-column DNase digestion was performed using the RNase-Free DNase Set (QIAGEN) to eliminate genomic DNA contamination. Total cDNA was synthesized using SuperScriptIII First-Strand Synthesis System (Invitrogen, Carlsbad, CA). The complete *BEN1* transcript was amplified using primers PRT1 and PRT2. *ACTIN2* was used as an internal control in RT-PCR (Table S1). The linear range of amplification for each gene transcript was determined by comparing samples obtained using different numbers of cycles. Lack of genomic and foreign DNA contamination was ascertained by using all RNA samples and water as a template in a PCR.

### Real-time quantitative RT-PCR analysis

For real-time quantitative RT-PCR analysis, Applied Biosystems 7500 Fast Real-Time PCR System (Applied Biosystems, Foster City, CA) was used. *BEN1* transcript was amplified using primers PQ1 and PQ2. *BAS1* transcript was amplified using primers PQ3 and PQ4 (Table S1). Internal control ubiquitin gene (*At5g15400*) was amplified using PCR Primers UBQ1 and UBQ2 (Table S1). PCR thermocycling program profile used was as following: initial denaturation at 95º for 20 sec, followed by 40 cycles of 95º for 3 sec, and 60º for 30 sec. Melt curve profile used melt curve analysis was as following: 95º for 15 sec, 60º for 1 min, 95º for 30 sec, and 60º for 15 sec.

### Amplification of T-DNA insertion

The T-DNA insertion fragment was amplified using PrimeSTAR GXL polymerase (Clonetech, Mountain View, CA). PCR thermocycling program profile used was as following: 35 cycles of 98º for 10 sec, 64º for 15 sec, and 68º for 10 min.

### Bisulfite conversion and DNA sequencing

DNA was converted using the EZ DNA Methylation-Lightning kit (Zymo Research, Irvine, CA). Converted DNA was amplified using Zymo*Taq* DNA polymerase (Zymo research) using primers listed in Table S1. The gel-purified PCR product was directly used for sequencing.

## Results

### The *ben1-1* T-DNA insertion mutation is unstable in the *bas1-2 sob7-1* background

**Figure 1:**
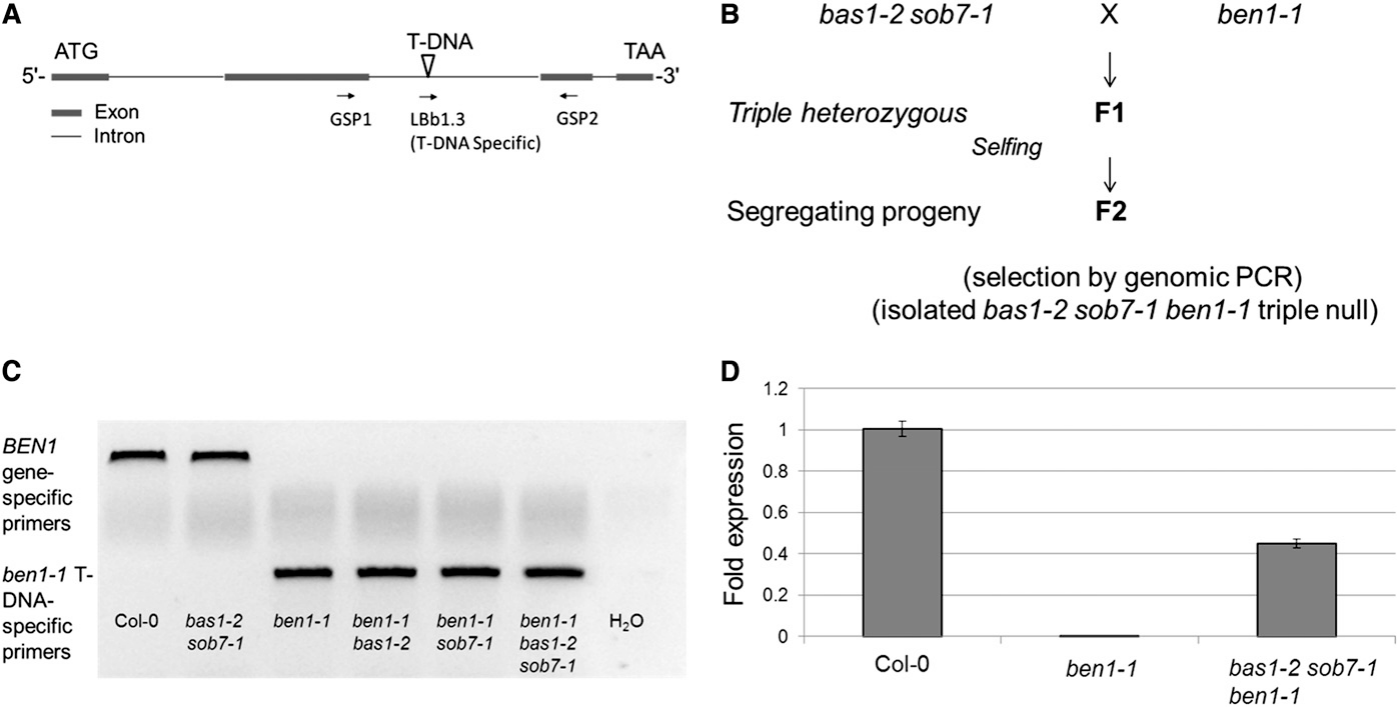
*ben1-1* mutation is unstable in the *bas1-2 sob7-1* background. (A) Graphical depiction of *BEN1* gene structure and location of T-DNA insertion in the *ben1-1* allele. (B) Depiction of the crossing scheme used to isolate *bas1-2 sob7-1 ben1-1* triple-mutant. (C) Example of the genetic analysis of *ben1-1* allele. (D) Quantitative RT-PCR analysis of *BEN1* expression in the wild type, *ben1-1* and triple mutant. Error bars indicate SEM.

A *bas1-2 sob7-1 ben1-1* triple mutant line was isolated from a cross between *bas1-2 sob7-1* double- and *ben1-1* single-mutants (Figure 1, A–C). Transcript levels of all three genes were examined to confirm that the triple mutant was a transcript-accumulation-null at all three loci. Surprisingly, quantitative RT-PCR showed that the triple mutant had significantly enhanced levels of *BEN1* transcript compared with the single *ben1-1* parental line, which showed very low levels of *BEN1* transcript accumulation (Figure 1D). Sequencing confirmed that the *BEN1* cDNA from both the wild type and the triple mutant was identical, indicating that the T-DNA containing second intron in the *ben1-1* allele in the triple mutant had spliced in a wild-type manner. These observations show that the *ben1-1* null mutation is unstable in the *bas1-2 sob7-1* background.

### The *BEN1* transcript levels stay steady in the reisolated *ben1-1* single mutant lines

**Figure 2:**
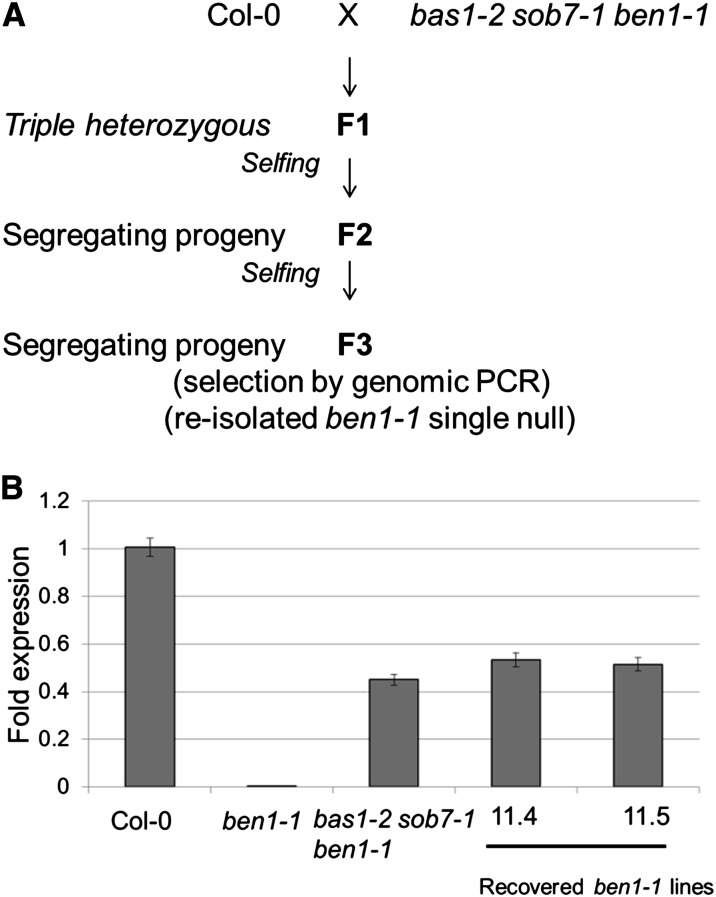
The *BEN1* transcript levels stay steady in the reisolated *ben1-1* single-null lines. (A) Depiction of the crossing scheme used to reisolate *ben1-1*. (B) Quantitative RT-PCR analysis of the *BEN1* transcript in the 5-d-old seedlings of Col-0, original *ben1-1*, *bas1-2 sob7-1 ben1-1*, F3# 11.4 (reisolated line), and F3# 11.5 (reisolated line) shows that the transcript accumulation levels in the reisolated lines (F3# 11.4 and F3# 11.5) remain stable at the same level as that of triple mutant line. Error bars indicate SEM.

The *bas1-2 sob7-1 ben1-1* triple mutant was back crossed to the wild type and in the segregating F3 progeny single *ben1-1* mutant lines (# 11.4 and # 11.5) were reisolated (Figure 2A). Interestingly, *BEN1* transcript levels in the two reisolated *ben1-1* lines were still enhanced relative to the original *ben1-1* line and similar to the *bas1-2 sob7-1 ben1-1* triple mutant (Figure 2B). The unstable nature of the *ben1-1* mutation in the *bas1-2 sob7-1 ben1-1* background, and the persistence of this phenotype in the reisolated *ben1-1* single-mutant lines, suggests that the *ben1-1* locus was modified either genetically or epigenetically in a heritable manner during the creation of the triple mutant.

### Reisolated *ben1-1* single-null lines are phenotypically different from the original *ben1-1* lines

**Figure 3:**
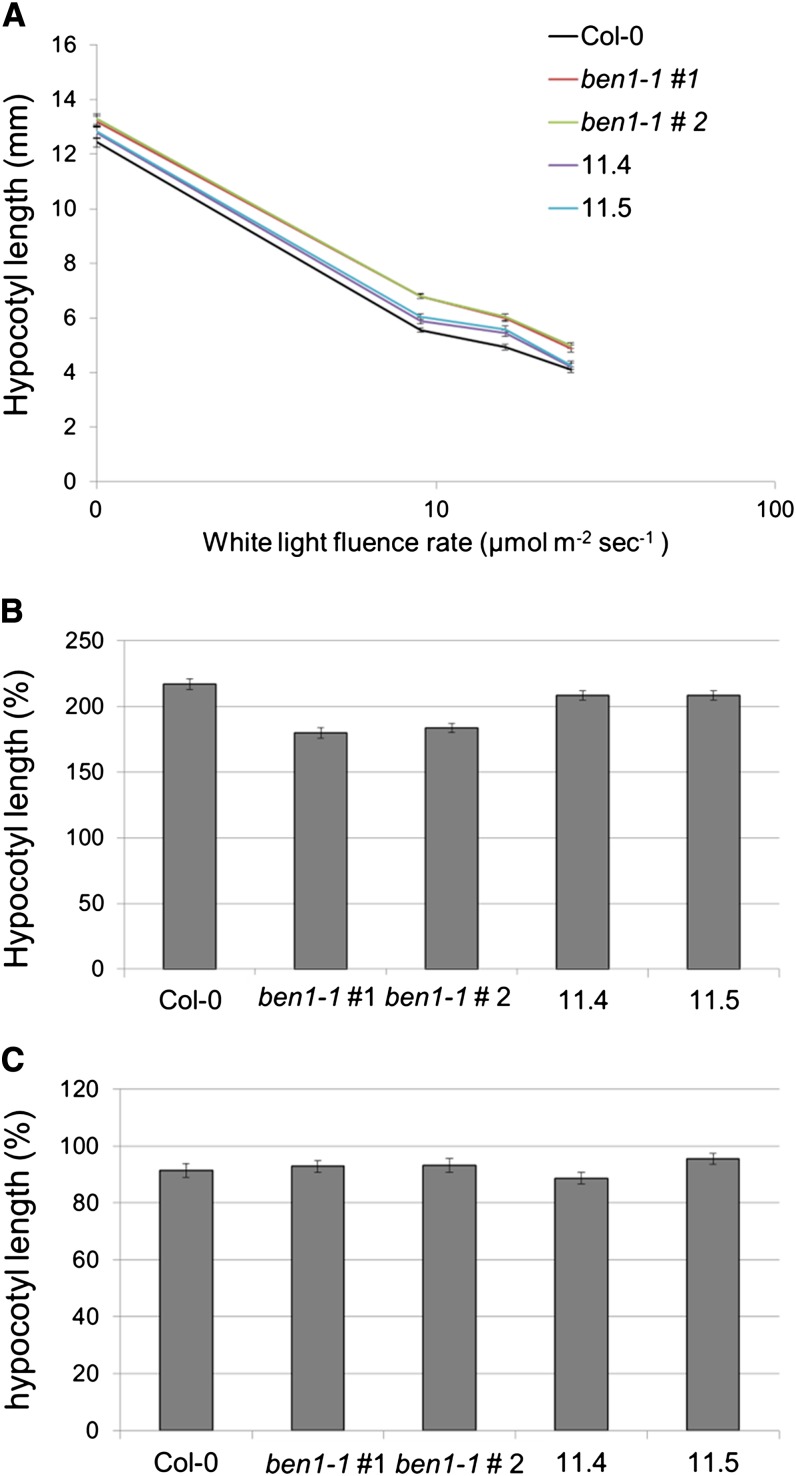
Reisolated *ben1-1* single-null lines are phenotypically different from the original *ben1-1* lines. (A) Fluence-rate analysis of the Col-0, original *ben1-1* line #1 and #2, and reisolated *ben1-1* lines at three different white-light fluence rates. The hypocotyl length of the reisolated *ben1-1* is not significantly different from original *ben1-1* (*P* > 0.05) in the dark. At all three white-light fluence rates, the hypocotyl length of the reisolated *ben1-1* is significantly different from original *ben1-1* lines (*P* < 0.05 in all cases). (B) Hypocotyl-elongation response of the reisolated and original *ben1-1* lines to the exogenous BL treatment (100 nm BL) in white-light. In white light (24 µmol m^−2^ sec^−1^) the hypocotyl-elongation response of the reisolated *ben1-1* is similar to the wild-type. The hypocotyl elongation is suppressed by BL treatment in the original *ben1-1* lines compared with the wild type. (C) In the dark the hypocotyl-elongation response of the reisolated *ben1-1* is not significantly different from original *ben1-1* (*P* > 0.05). To calculate the percentage of BL-negative-control hypocotyl length (as in B and C), each seedling value in BL treatment experiment was normalized to the average of the same genotype in BL negative control experiment. The resulting group of values was used to calculate SEM.

To test the hypothesis that the reisolated *ben1-1* mutant lines are also phenotypically different from the original *ben1-1* allele, fluence-rate response analysis of the hypocotyl-elongation response was studied in these lines (Figure 3A). The two reisolated *ben1-1* lines showed attenuated hypocotyl-elongation phenotypes compared with the two original *ben1-1* lines (Figure 3A). At all three fluence rates of white light the reisolated lines displayed significantly shorter hypocotyls than the original *ben1-1* lines (*P* < 0.05). There were no significant differences in hypocotyl-length between the original and the reisolated *ben1-1* lines in the dark (*P* > 0.05). Hypocotyl-elongation responses to exogenous BL treatments were also examined in these lines (Figure 3, B and C). In white light, two original *ben1-1* lines showed reduced hypocotyl-elongation compared with the wild type, whereas the behavior of the two reisolated *ben1-1* lines was similar to the wild type (Figure 3B). The two original and reisolated *ben1-1* lines did not respond differently to BL treatment in darkness (Figure 3C). To test the hypothesis that the epigenetically modified *ben1-1* locus is stably inherited, the attenuated hypocotyl-elongation phenotype associated with this locus was also analyzed in the next generation in various genotypic lines. These results indicated that the attenuated hypocotyl-elongation phenotype of the reisolated *ben1-1* lines was stably inherited to the next generation (Figure S1).

### *BEN1* transcript accumulation is not feedback regulated by BRs

**Figure 4:**
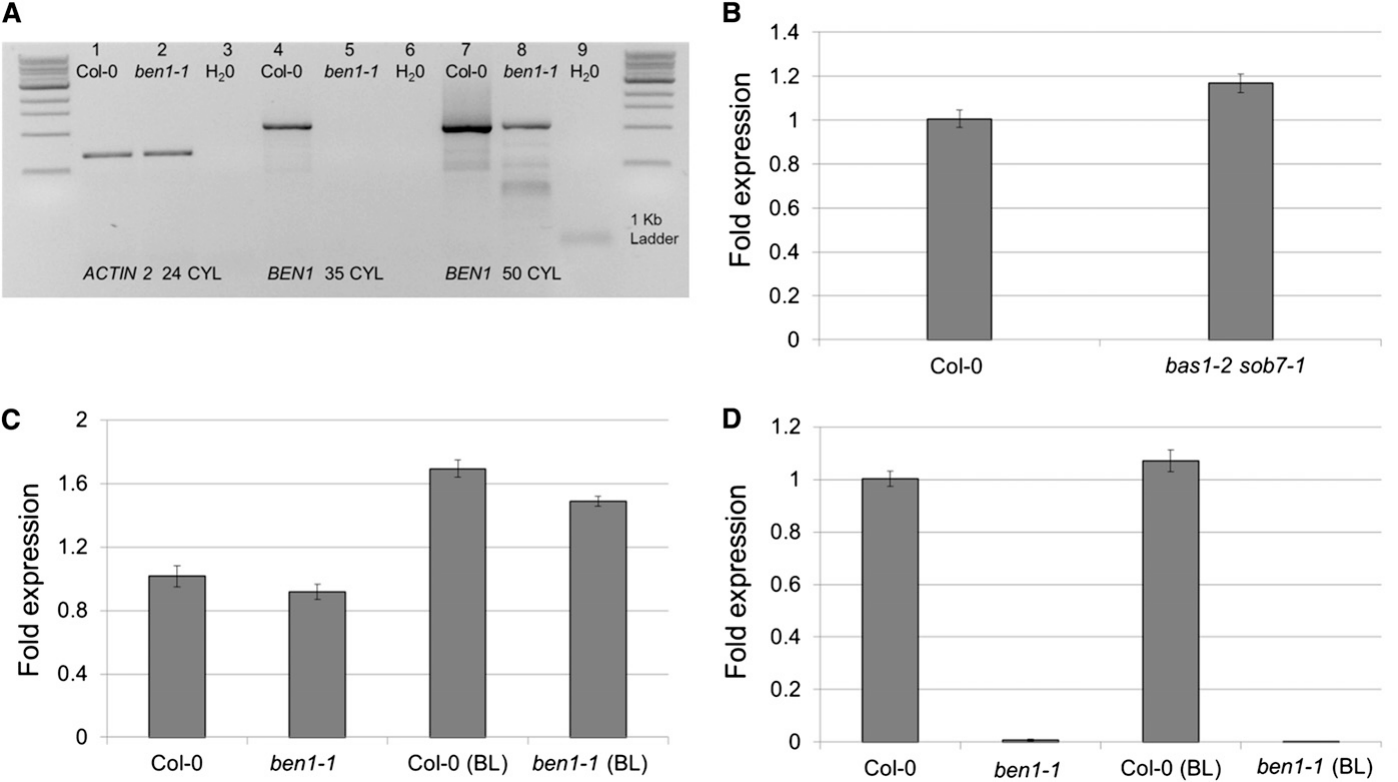
The *ben1-1* is a leaky mutant based on RT-PCR analysis and the enhanced T-DNA-containing-intron splicing in recovered *ben1-1* is not induced by BR feedback regulation. (A) Primer pair, PRT1, and PRT2 was used to amplify full-length coding region of *BEN1* transcript. Amplification of *ACTIN2* transcript was used as a cDNA loading control (Lane 1 and 2). At 35 cycles no product is detected in the *ben1-1* lane (Lane 5) compared with the Col-0 lane (Lane 4). After 50 cycles, the *BEN1* transcript is detectable in the *ben1-1* lane (Lane 8). Primer sequence information is given in Table S1. (B) *BEN1* expression is not affected in the *bas1-2 sob7-1* double-null background relative to the wild type background. (C) Exogenous BL treatment induces *BAS1* expression in Col-0 and *ben1-1* background. (D) Exogenous BL treatment does not induces *BEN1* expression in Col-0 and *ben1-1* background. Error bars indicate the standard error of the mean (SE).

It is possible that the original *ben1-1* was a leaky mutation to start with and that the elevated BR levels in the *bas1-2 sob7-1* genetic background further increased *BEN1* transcript accumulation which amplified the leakiness of *ben1-1*. To test this hypothesis first, a modified RT-PCR approach was used to detect the rare wild-type-spliced *BEN1* transcripts in the original *ben1-1* line. As was previously published, after 35 PCR cycles no product was detected in the *ben1-1* line (Yuan *et al.* 2007; Figure 4A). However after 50 PCR cycles, a *BEN1* amplification product could be detected, showing the presence of low level wild-type-spliced *BEN1* transcript in the original *ben1-1* line (Figure 4A).

To further test the hypothesis that the BR feed-back regulation in the *bas1-2 sob7-1* background was causing the instability of the *ben1-1* mutation, *BEN1* transcript levels were examined in the wild-type and the *bas1-2 sob7-1* double-null line using quantitative RT-PCR (Figure 4B). *BEN1* transcript levels were also examined in response to exogenous BL treatment in the wild type and the original *ben1-1* line (Figure 4D). Results showed that compared with the wild type, *BEN1* transcript levels were not significantly enhanced in the *bas1-2 sob7-1* background (Figure 4B). Moreover, unlike *BAS1* (Figure 4C) exogenous BL treatment did not result in any increase in *BEN1* transcript accumulation levels in the wild type (Figure 4D). In addition, exogenous BL treatment also did not result in an increased accumulation of wild-type spliced *BEN1* transcript in the original *ben1-1* line. These observations clearly show that BR-mediated feed-back regulation in the *bas1-2 sob7-1* background is not responsible for the *ben1-1* mutation instability in the triple mutant.

### T-DNA *trans*-interactions likely cause *ben1-1* instability

Given that *BEN1* transcript accumulation is not feedback regulated by changing levels of BRs, it is possible that T-DNA *trans*-interactions between T-DNA insertions located in the *bas1-2*, *sob7-1* and *ben1-1* alleles can also lead to *ben1-1* mutation instability in the triple-mutant. Epigenetic silencing of the T-DNA-located resistance markers often indicates the presence of T-DNA *trans*-interactions (Daxinger *et al.* 2008).

**Figure 5:**
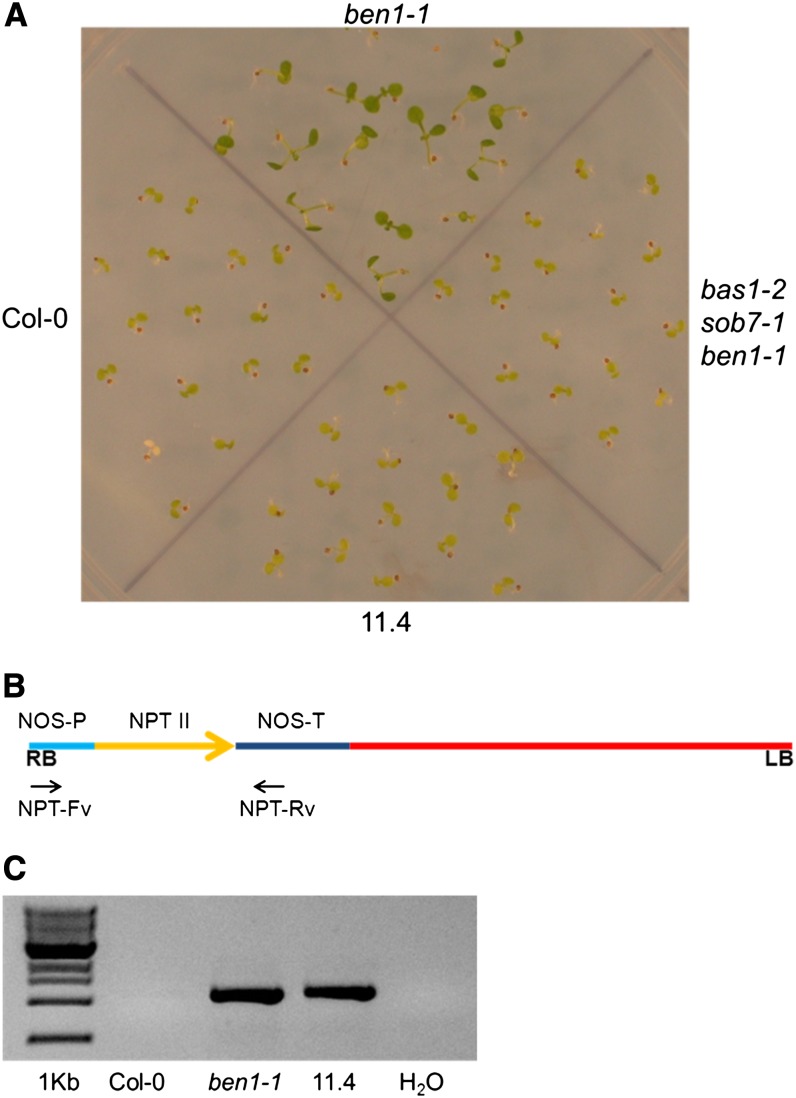
The kanamycin resistance trait, conferred by the T-DNA resistance marker *NPTII*, is absent in the *bas1-2 sob7-1 ben1-1* and the recovered *ben1-1* line. (A) Seedlings were grown on the plant media containing kanamycin for 7 d before photographing. (B) The graphic depiction of the location of the primers used to amplify *NPTII* from the T-DNA. (C) The gel image shows that genomic PCR amplifies bands of equal size from original *ben1-1* and the recovered *ben1-1* lines.

To test this hypothesis, *NPTII* resistance marker gene function in the *ben1-1* T-DNA was examined by planting seeds of the wild type, original *ben1-1*, triple-mutant and the reisolated *ben1-1* (# 11.4) lines on kanamycin containing plant growth media. Seedlings of all the lines except the original *ben1-1* line showed complete kanamycin sensitivity (Figure 5A). This observation suggests the presence of T-DNA *trans*-interactions resulting in silencing of the resistance marker in the triple- and the reisolated *ben1-1* single-mutant lines. Another possible explanation for these observations is that the *NPTII* locus had been mutated during the crossing and recombination process. To test this second possibility, the *NPTII* gene was amplified and sequenced from both the original and the reisolated *ben1-1* lines (Figure 5, B and C). The *NPTII* gene sequence was found to be unaltered in the original and the reisolated *ben1-1* lines (Figure S2). These observations confirm that the loss of the kanamycin resistance marker in the triple-mutant and the reisolated *ben1-1* lines is the effect of the presence of T-DNA *trans*-interactions in the triple-mutant.

### The *ben1-1* T-DNA is differentially methylated in the original and the reisolated *ben1-1* lines

**Figure 6:**
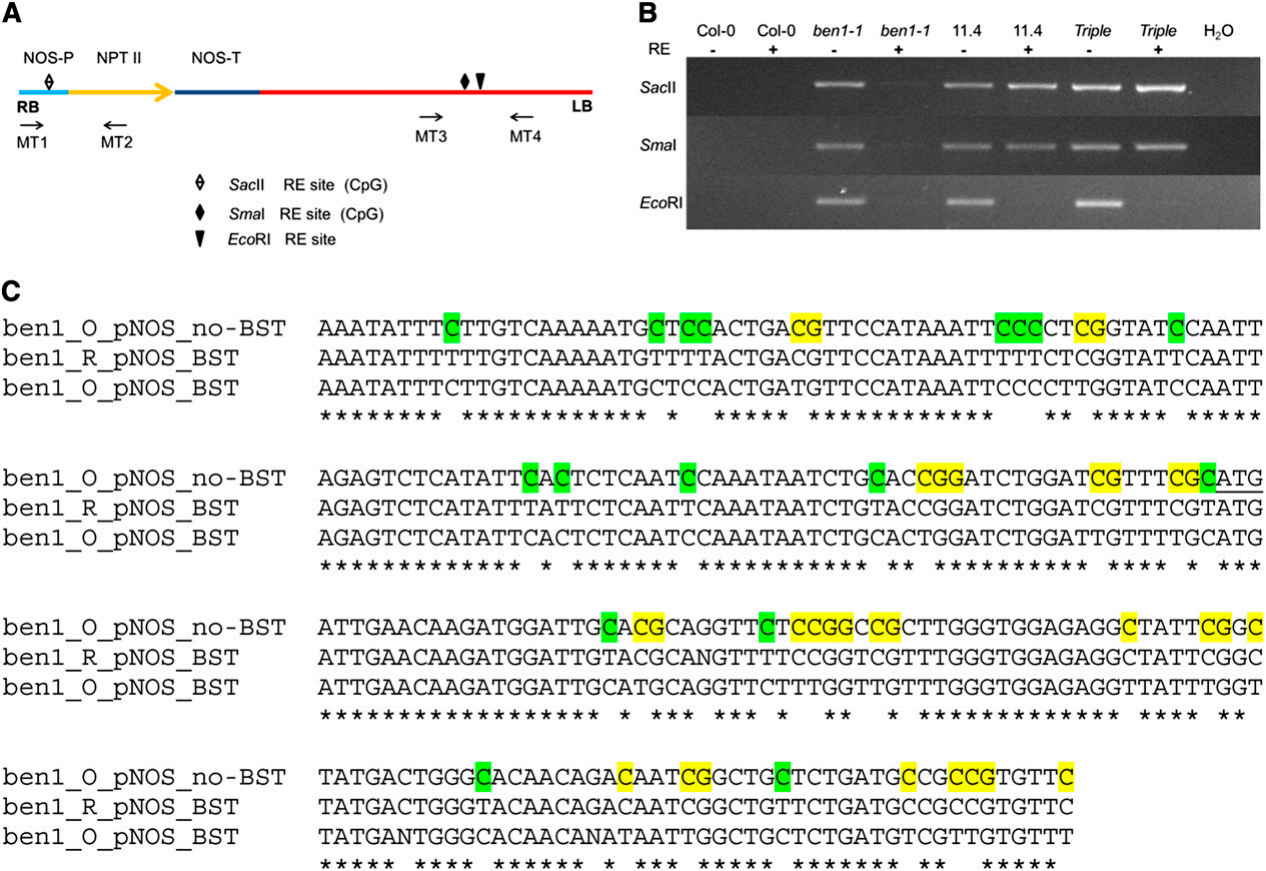
Genomic DNA in the *NOS* Promoter region of the T-DNA insertion in the recovered *ben1-1* shows methylation. (A) Graphic depiction of RE sites and the primer pair locations in the T-DNA used for the methylation study. (B) Genomic PCR amplification from the undigested and the restriction digested DNA of the Col-0, original *ben1-1*, recovered *ben1-1* (11.4), and the *bas1-2 sob7-1 ben1-1* (triple). Primer pairs MT1 and MT2 were used to test methylation at the *Sac*II site. Primer pairs MT3 and MT4 were used to test methylation at the *Sma*I site. Primer pairs MT3 and MT4 were also used to amplify genomic DNA digested with nonmethylation-sensitive *Eco*RI. PCR amplification, by using *Sac*II site and *Sma*I/*Eco*RI flanking PCR primers (Table S1), is seen only from the digested genomic DNA of the recovered *ben1-1*. (C) *NOS* promoter and adjacent *NPTII* DNA was amplified using primers pNOS-BS-F and pNOS-BS-F (Table S1) from the bisulfite converted genomic DNA of the pretriple and the posttriple *ben1-1* lines. The nonbisulfite-treated pretriple *ben1-1* genomic DNA also was amplified and sequenced for comparison. The differentially methylated cytosine nucleotides are color coded (Yellow are differentially methylated in posttriple *ben1-1* and green are differentially demethylated in the posttriple *ben1-1* line compared with the pretriple *ben1-1* line). Guanine nucleotides are also color coded when they follow methylated cytosine nucleotides to show the CG context. The translation initiation codon ATG for *NPTII* gene is underlined. BST, bisulfite treatment; ben1_O, pretriple *ben1-1*; and ben1_R, posttriple *ben1-1*.

Silencing of the *NPTII* resistance marker gene in the triple-mutant and reisolated *ben1-1* lines suggest that this genomic region has been differentially modified by methylation in the triple-mutant and the reisolated *ben1-1* T-DNAs. To test this hypothesis, genomic DNA from the wild type, triple-, original, and reisolated *ben1-1* single-mutant lines was digested with CpG methylation sensitive and insensitive restriction enzymes (REs). The restriction site−specific sequences for the CpG methylation sensitive enzymes *Sac*II and *Sma*I are present in the *NPTII* promoter and in proximity to the left border of the *ben1-1* inserted T-DNA respectively (Figure 6A). The restriction site−specific sequence for the CpG methylation insensitive enzyme *Eco*RI is located adjacent to the *Sma*I RE site (Figure 6A). After digestion with REs, genomic DNA was used as a PCR template in which primers flanking the corresponding RE sites were used to prime the amplification (Figure 6A). Results of the PCR amplification showed that the triple mutant and reisolated *ben1-1* T-DNA were more resistant to RE digestion by both the CpG methylation sensitive REs compared with the original *ben1-1* T-DNA (Figure 6B), whereas there were no differences among these genotypes with regard to *Eco*RI digestion (Figure 6B). These observations demonstrate that the *ben1-1* T-DNA in the triple- and the reisolated *ben1-1* single-mutant is differentially methylated in both the promoter region of *NPTII* gene as well as in the adjacent regions.

To further understand the genomic context of cytosine methylation, bisulfite conversion and sequencing was performed on the genomic DNA of the pretriple and posttriple *ben1-1* lines. The results demonstrated that cytosine nucleotides in the *NOS* (*NOPALINE SYNTHASE*) promoter and the adjacent *NPTII* genomic DNA were differentially methylated (in the CG and CCG context) in the posttriple *ben1-1* compared with the pretriple *ben1-1* (Figure 6C). Interestingly, many cytosine nucleotides were also differentially demethylated (in a non CG and CCG context) in the posttriple *ben1-1* compared with the pretriple *ben1-1* line (Figure 6C). Genomic DNA in the part of the *BEN1* promoter (immediately upstream of the translation start codon) and second exon-intron junction of the *BEN1* gene were also studied for cytosine methylation. The sequencing results showed that cytosine nucleotides in these regions were not methylated in both the pretriple and the *bas1-2 sob7-1 ben1-1* triple-mutant lines (Figure S3).

### T-DNA size and structure remains unchanged in the original and the reisolated *ben1-1* lines

**Figure 7:**
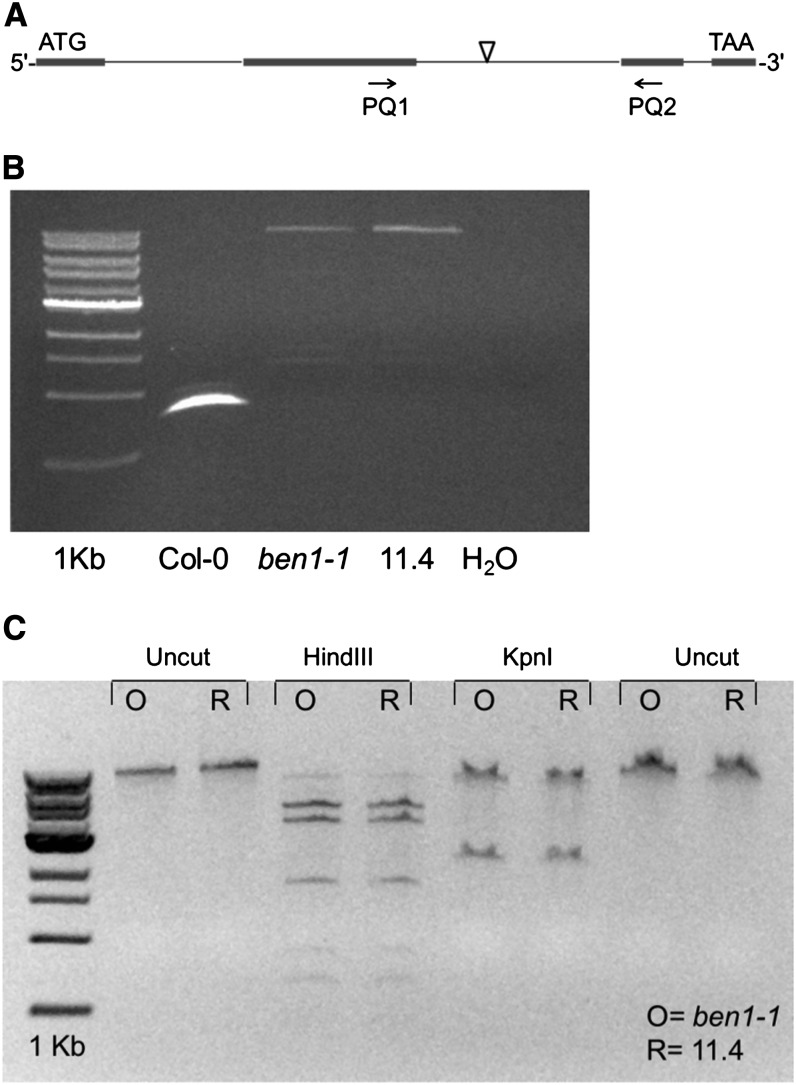
The size and sequence of the T-DNA insertion remains unchanged in the pretriple and posttriple *ben1-1* lines. (A) Graphic depiction of the location of the second intron flanking primer pair used for amplification in the *ben1-1* allele. (B) The genomic PCR using the second intron flanking primers amplify equal size band from the original *ben1-1* and the recovered *ben1-1* line. (C) The original and the recovered *ben1-1* alleles show identical restriction digestion pattern. The PCR amplification product from gel image (B) was gel purified and restriction digested with *Hind*III and *Kpn*I.

In another scenario, a shortening of T-DNA insertion due to unequal recombination during the creation of the triple-mutant could also result in enhanced *BEN1* transcript levels compared with the original *ben1-1* line. To test this hypothesis, the inserted T-DNA was amplified from the original and the reisolated *ben1-1* lines using primers anchored in the second and third exons of the *BEN1* gene (Figure 7A). The PCR results show that, using wild-type DNA as a PCR template gave a band of the size expected in the case of no T-DNA insertion (Figure 7B). On the other hand, both the original and reisolated *ben1-1* lines showed a large band of the same size, indicating the presence of approximately two T-DNA insertions in each case (Figure 7B). To further test the possibility of any re-arrangement of the T-DNA in original *vs.* the reisolated *ben1-1*, the large T-DNA containing amplification product of the extended PCR (Figure 7B) was purified and cut using two different REs. Identical restriction pattern of the two amplification products showed the absence of any T-DNA rearrangement between the original and the reisolated T-DNA insertions (Figure 7C). These experiments suggest that the T-DNA size and structure have not changed between the original and the reisolated *ben1-1* lines.

## Discussion

As a part of our analysis of BR catabolic enzymes, we generated a genetic triple mutant from a cross between the *bas1-2 sob7-1* double-null and *ben1-1*. Surprisingly, the originally stable *ben1-1* mutation was converted to a partial loss-of-function mutation in the triple mutant background (Figure 1D). In addition to this report, the phenomenon of T-DNA mutant instability due to trans T-DNA interactions was also recently described by two groups with different sets of mutants (Gao and Zhao 2012; Xue *et al.* 2012). To study the genetics of *ben1-1* instability, we reisolated the *ben1-1* mutation by back crossing the triple mutant with the wild type (Figure 2). Molecular analysis of reisolated *ben1-1* lines showed that the unstable state of the *ben1-1* mutation in the triple mutant is maintained in an otherwise wild-type background. The *BEN1* expression in the triple and the reisolated *ben1-1* was intermediate between the wild type and the original *ben1-1* mutant. These observations reflect a known phenomenon, that the strength of T-DNA *trans*-interactions can be locus specific (Xue *et al.* 2012).

The original and the reisolated *ben1-1* single lines also differed phenotypically (Figure 3). The original *ben1-1* single-mutant line displays an aberrant hypocotyl-elongation phenotype in white light (Yuan *et al.* 2007). Compared with the original *ben1-1* allele, the reisolated *ben1-1* lines displayed significantly different hypocotyl-elongation phenotypes in white-light fluence-rate response experiments as well as in a BL dose-response treatment (Figure 3, A–C). These genetic and molecular observations suggest that the changes in the reisolated *ben1-1* are of an epigenetic nature. However, the exact mechanism leading to the enhanced T-DNA-containing intron-splicing is unknown.

In the case of intronic T-DNA insertion mutations, presence of wild-type-spliced transcript is not uncommon (Wang 2008). Additionally, conditional mutant instability has been observed in an intronic T-DNA insertion mutation in the *OPR3* (*OPDA REDUCTASE 3*) gene (Chehab *et al.* 2011). *opr3* is an intronic T-DNA insertion mutant of *OPR3* which does not produce any detectable jasmonic acids (JAs) and acts as a complete loss-of-function allele under normal conditions. However, the *opr3* allele becomes unstable when mutant plants are infected with a fungus (Chehab *et al.* 2011). This suggests that environment may play a role in the phenomenon of T-DNA insertion mutant instability.

To test the hypothesis that the epigenetic transformation of *ben1-1* in the triple mutant could be due to elevated BR content in the *bas1-2 sob7-1* genetic background, we studied *BEN1* expression in the *bas1-2 sob7-1* double-null. *BEN1* expression was not significantly affected in the *bas1-2 sob7-1* background (Figure 4B), suggesting that *BEN1* is not strongly feedback regulated by changes in BR levels. This finding suggests that increased levels of BR in *bas1-2 sob7-1* (Turk *et al.* 2005) did not result in *ben1-1* instability in the triple-mutant background (Figure 4B). Another possible explanation is that *BEN1* is not expressed in the same tissues where both *BAS1* and *SOB7* are expressed. *bas1-2* and *sob7-1* single-null mutants do not have altered BR levels. Because BAS1 and SOB7 expression patterns do not overlap completely (Sandhu *et al.* 2012), it is possible that all tissues in the *bas1-2 sob7-1* double-null do not have altered BR levels. Analysis of the publically available microarray data suggests that *BEN1* transcript accumulation is not significantly affected by BL treatment (Winter *et al.* 2007). To complement the microarray data analysis, we also studied *BEN1* expression in the wild type and the original *ben1-1* single-mutant in response to exogenous BL (Figure 4D). *BAS1* expression was also studied in the same experiment to compare *BEN1* gene expression response to a known BR-feedback regulated gene. The results of gene expression analysis also showed that *BEN1* expression is not affected by BL treatment and hence it is highly unlikely that BR feed-back regulation is responsible for the *ben1-1* instability (Figure 4, C and D).

Because the mutagenic T-DNA is the same in *bas1-2*, *sob7-1*, and *ben1-1* (Alonso *et al.* 2003), a possible cause of *ben1-1* instability could be the effect of T-DNA *trans*-interactions. As documented previously, T-DNA *trans*-interactions can result in the loss of antibiotic resistance (Daxinger *et al.* 2008). In fact, the original *ben1-1* mutant seedlings showed kanamycin resistance, whereas the reisolated lines as well as the triple were completely sensitive to kanamycin (Figure 5A). Interestingly, the loss of kanamycin resistance also showed an epigenetic pattern of inheritance. Similar loss of kanamycin resistance is observed in the case of *yuc1-1* (*yucca1-1*) and *ag-TD* (*agamous-TDNA* insertion mutant) interactions (Gao and Zhao 2012).

Silencing of homologous genes is known to be an effect of T-DNA *trans*-interactions (Daxinger *et al.* 2008). The silencing of a GUS reporter gene in activation tagging lines with multiple insertions was also found to be associated with methylation of the CaMV35S enhancer elements in those T-DNAs (Chalfun-Junior *et al.* 2003). Homology-induced silencing is also associated with a phenomenon known as RNA-directed DNA methylation in the region of RNA-DNA homology (Wasseneger 2000). In fact, the promoter region of the reisolated *ben1-1* line T-DNA showed cytosine methylation, which is not the case with the original *ben1-1* T-DNA (Figure 6B). The T-DNA methylation was also observed in the region adjacent to the *NPTII* locus (Figure 6B). The spread of DNA methylation to adjacent parts of the RNA-DNA homology region has previously been documented (Wasseneger 2000). Another possibility is that apart from the *NPTII* gene, the mRNA transcribed from other parts of the T-DNA is also present in the *ben1-1* mutant.

T-DNA *trans*-interactions have also been associated with instability of an intronic T-DNA insertion mutant (Gao and Zhang 2012; Xue *et al.* 2012). However, currently there is no direct evidence which supports that the mutant instability is caused directly by T-DNA *trans*-interactions. The most significant difference in the original and the reisolated *ben1-1* lines is the presence of methylation in the reisolated *ben1-1* T-DNA region (Figure 6). Methylation has also been shown to be responsible for the instability of the *cobra* SALK T-DNA mutant (Xue *et al.* 2012). The *epicob-6* (*epigenetic cob-6*) mutant phenotype was reverted back to the *cob-6* phenotype, when grown on media containing methylation inhibitors. In addition, the *epicob-6* reversion phenomenon was shown to happen in genetic backgrounds lacking enzymes required for CG and CHG DNA methylation (Xue *et al.* 2012). In agreement with Xue *et al.* (2012), our bisulfite sequencing analysis also showed that cytosine methylation in the unstable *ben1-1* T-DNA was in the CG and CCG context (Figure 6C). Interestingly, cytosine de-methylation (in the non CG context) was also observed in the posttriple *ben1-1* line suggesting that this phenomenon may also contribute to epigenetic instability of the *ben1-1* T-DNA insertion.

One mechanism that can explain this epigenetic suppression is RNA/RNA interactions between a long transcribed RNA from the gene and the T-DNA and a complementary RNA from the T-DNA (Gao and Zhao 2012). Our observation that cytosine methylation is located only in the *ben1-1* T-DNA region and absent in other parts of the *ben1-1* locus (Figure 6 and Figure S3) supports this hypothesis. This phenomenon is similar to the epigenetic changes in gene expression caused by long intronic non-coding RNAs (Heo and Sung 2010). This mechanism may also cause instability for exonic T-DNA insertion mutations.

Another possible mechanism is that methylation of the T-DNA region is altering the splicing efficiency of the T-DNA containing intron. The splicing efficiency could be affected by a change in the size of the T-DNA insertion due to events such as unequal recombination. However, the size of T-DNA insertion and structure is unchanged in the pre- and posttriple *ben1-1* mutant lines (Figure 7). The most significant difference in the original and the reisolated *ben1-1* lines is the presence of methylation in the reisolated *ben1-1* T-DNA region (Figure 6).

RNA-induced DNA methylation has been associated with chromatin modification (Aufsatz *et al.* 2002a,b). Chromatin modifications in turn have been linked to the regulation of pre-mRNA splicing by many studies (for review, see Hnilicová and Staněk 2011). For example, alternative splicing in the CD44 gene required proper recruitment of argonaute RNAi proteins to the CD44 transcribed regions (Ameyar-Zazoua *et al.* 2012). Therefore, it is possible that the methylation of the T-DNA region is altering the splicing efficiency of the T-DNA containing intron. However, more studies are required to test the association between T-DNA methylation and the T-DNA containing intron splicing. Primarily, this study indicates the need for more caution while using T-DNA insertional mutants. Ultimately, this study also highlights a need for an alternative high-throughput technology for generating and cataloging genetic mutants. In the future, EMS mutagenesis coupled with improved low-price, high-throughput sequencing technologies may provide additional alleles for genetic analysis. Future directions for this study will include isolation of additional mutations in the *BEN1* locus. An EMS mutagenesis approach (Street *et al.* 2008) using the activation-tagged mutant *ben1-D* will be used to isolate true null-alleles of *BEN1*.

## Supplementary Material

Supporting Information
