# Supplementary material for: The ben1-1 Brassinosteroid-Catabolism Mutation Is Unstable Due to Epigenetic Modifications of the Intronic T-DNA Insertion
Source: G3 (Bethesda). 2013 Sep 1;3(9):1587–95. doi: 10.1534/g3.113.006353 (PMC3755919; doi:10.1534/g3.113.006353)
Supplement: Supporting Information [file supp_3_9_1587__index.html]

The ben1-1 Brassinosteroid-Catabolism Mutation Is Unstable Due to Epigenetic Modifications of the Intronic T-DNA Insertion — Supporting Information 

# The *ben1-1* Brassinosteroid-Catabolism Mutation Is Unstable Due to Epigenetic Modifications of the Intronic T-DNA Insertion

## Supporting Information for Sandhu, Koirala, and Neff, 2013

**Files in this Data Supplement:**

- Supporting Information - Figures S1-S3, Files S1-S8, and Table S1 (PDF, 727 KB)
- Figure S1 - The attenuated hypocotyl-elongation phenotype of the re-isolated *ben1-1* lines is stably inherited to the next generation. (PDF, 114 KB)
- Figure S2 - *NPTII* gene sequence did not change in the pre-triple and post-triple *ben1-1* mutants. (PDF, 105 KB)
- Figure S3 - Genomic DNA in the *BEN1* Promoter and *BEN1* 2nd Exon-Intron junction does not show any methylation in both the pre-triple *ben1-1* and the *bas1-2 sob7-1 ben1-1* triple-mutant lines. (PDF, 101 KB)
- File S1 - Bisulfite *BEN1* 2nd exon raw sequences (PDF, 397 KB)
- File S2 - Bisulfite sequences of the *BEN1* promoter (PDF, 409 KB)
- File S3 - Bisulfite pNOS raw sequences (PDF, 116 KB)
- File S4 - *NPTII* gene sequencing V2 (PDF, 331 KB)
- Table S1 - Table of PCR primer sequences used in the study (PDF, 56 KB)
- File S5 - *BEN1* BL-100 analysis (.xlsx, 62 KB)
- File S6 - Fluence rate analysis (.xlsx, 45 KB)
- File S7 - Next generation unstable *ben1-1* Hypocotyl data (.xlsx, 46 KB)
- File S8 - Real time RT-PCR Expression data (.xlsx, 41 KB)
