# Supplementary material for: The ben1-1 Brassinosteroid-Catabolism Mutation Is Unstable Due to Epigenetic Modifications of the Intronic T-DNA Insertion
Source: G3 (Bethesda). 2013 Sep 1;3(9):1587–95. doi: 10.1534/g3.113.006353 (PMC3755919; doi:10.1534/g3.113.006353)
Supplement: Supporting Information [file supp_g3.113.006353_006353SI.pdf]

G3·Genes | Genomes | Genetics

The *ben1-1* brassinosteroid-catabolism mutation is unstable due to epigenetic modifications of the intronic T-DNA insertion

Kulbir Singh Sandhu[*], Pushpa Sharma Koirala[*] and Michael M. Neff[*1]

[*]Department of Crop and Soil Sciences
Washington State University
Pullman, WA 99164, US

[1]Corresponding Author:
Michael M. Neff
Department of Crop and Soil Sciences
Washington State University
Pullman, WA 99164
Phone: 509-335-7705
Fax: 509-335-8674
E-mail: mmneff@wsu.edu

DOI: 10.1534/g3.113.006353

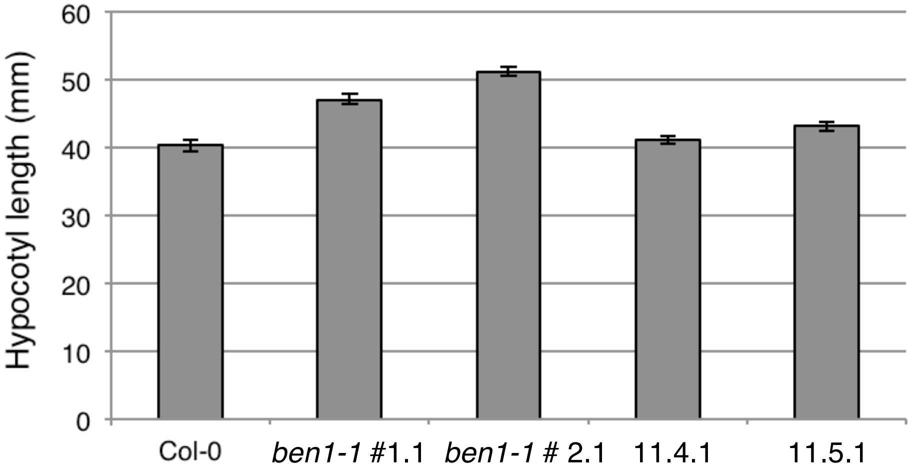

Figure S1 The attenuated hypocotyl-elongation phenotype of the re-isolated *ben1-1* lines is stably inherited to the next generation. The hypocotyl-elongation phenotype of progeny from the re-isolated *ben1-1* lines is significantly different from the original *ben1-1* lines ($p < 0.05$). Seedlings were grown in darkness or 9 µmol m^{-2} sec^{-1} of white-light for five days before being digitized and measured. Hypocotyl lengths were then normalized to seedlings grown in the dark and expressed as a percentage change. Error bars represent standard error (SE). To calculate SE, each seedling value in light was normalized to the average of the genotype in dark. The resulting group of values was used to calculate standard error (SE).

```
ben1_O_NPTII-F1  NNNNNNNACNTNNNNGAGCCACTCAGCCGCGGGTTTCTGGAGTTTAATGAGCTAAGCACA
ben1_R_NPTII-F1  NNNNNNNACNTNNANGANCCNCTCAGCCGCGGGTTTCTGGAGTTTAATGAGCTAAGCACA
                 ************* *** ** ***************************************

ben1_O_NPTII-F1  TACGTCAGAAACCATTATTGCGCGTTCAAAAGTCGCCTAAGGTCACTATCAGCTAGCAAA
ben1_R_NPTII-F1  TACGTCAGAAACCATTATTGCGCGTTCAAAAGTCGCCTAAGGTCACTATCAGCTAGCAAA
                 ************************************************************

ben1_O_NPTII-F1  TATTTCTTGTCAAAAATGCTCCACTGACGTTCCATAAATTCCCCTCGGTATCCAATTAGA
ben1_R_NPTII-F1  TATTTCTTGTCAAAAATGCTCCACTGACGTTCCATAAATTCCCCTCGGTATCCAATTAGA
                 ************************************************************

ben1_O_NPTII-F1  GTCTCATATTCACTCTCAATCCAAATAATCTGCACCGGATCTGGATCGTTTCGCATGATT
ben1_R_NPTII-F1  GTCTCATATTCACTCTCAATCCAAATAATCTGCACCGGATCTGGATCGTTTCGCATGATT
                 ************************************************************

ben1_O_NPTII-F1  GAACAAGATGGATTGCACGCAGGTTCTCCGGCCGCTTGGGTGGAGAGGCTATTCGGCTAT
ben1_R_NPTII-F1  GAACAAGATGGATTGCACGCAGGTTCTCCGGCCGCTTGGGTGGAGAGGCTATTCGGCTAT
                 ************************************************************

ben1_O_NPTII-F1  GACTGGGCACAACAGACAATCGGCTGCTCTGATGCCGCCGTGTTCCGGCTGTCAGCGCAG
ben1_R_NPTII-F1  GACTGGGCACAACAGACAATCGGCTGCTCTGATGCCGCCGTGTTCCGGCTGTCAGCGCAG
                 ************************************************************

ben1_O_NPTII-F1  GGGCGCCCGGTTCTTTTTGTCAAGACCGACCTGTCCGGTGCCCTGAATGAACTGCAGGAC
ben1_R_NPTII-F1  GGGCGCCCGGTTCTTTTTGTCAAGACCGACCTGTCCGGTGCCCTGAATGAACTGCAGGAC
                 ************************************************************

ben1_O_NPTII-F1  GAGGCAGCGCGGCTATCGTGGCTGGCCACGACGGGCGTTCCTTGCGCAGCTGTGCTCGAC
ben1_R_NPTII-F1  GAGGCAGCGCGGCTATCGTGGCTGGCCACGACGGGCGTTCCTTGCGCAGCTGTGCTCGAC
                 ************************************************************

ben1_O_NPTII-F1  GTTGTCACTGAAGCGGGAAGGGACTGGCTGCTATTGGGCGAAGTGCCGGGGCAGGATCTC
ben1_R_NPTII-F1  GTTGTCACTGAAGCGGGAAGGGACTGGCTGCTATTGGGCGAAGTGCCGGGGCAGGATCTC
                 ************************************************************

ben1_O_NPTII-F1  CTGTCATCTCACCTTGCTCCTGCCGAGAAAGTATCCATCATGGCTGATGCAATGCGGCGG
ben1_R_NPTII-F1  CTGTCATCTCACCTTGCTCCTGCCGAGAAAGTATCCATCATGGCTGATGCAATGCGGCGG
                 ************************************************************

ben1_O_NPTII-F1  CTGCATACGCTTGATCCGGCTACCTGCCCATTCGACCACCAAGCGAAACATCGCATCGAG
ben1_R_NPTII-F1  CTGCATACGCTTGATCCGGCTACCTGCCCATTCGACCACCAAGCGAAACATCGCATCGAG
                 ************************************************************

ben1_O_NPTII-F1  CGAGCACGTACTCGGATGGAAGCCGGTCTTGTCGATCAGGATGATCTGGACGAAGAGCAT
ben1_R_NPTII-F1  CGAGCACGTACTCGGATGGAAGCCGGTCTTGTCGATCAGGATGATCTGGACGAAGAGCAT
                 ************************************************************
```

```
ben1_O_NPTII-F2  ACCAAGCGAAACATCGCATCGAGCGAGCACGTACTCGGATGGAAGCCGGTCTTG-TCGAT
ben1_R_NPTII-F2  ACCAAGCGAAACATCGCATCGAGCGAGCACGTACTCGGATGGAAGCCGGTCTNNGTCGAT
                 ****************************************************   *****

ben1_O_NPTII-F2  CAGGATGATCTGGACGAAGAGCATCAGGGGCTCGCGCCAGCCGAACTGTTCGCCAGGCTC
ben1_R_NPTII-F2  CAGGATGATCTGGACGAAGAGCATCAGGGGCTCGCGCCAGCCGAACTGTTCGCCAGGCTC
                 ************************************************************

ben1_O_NPTII-F2  AAGGCGCGCATGCCCGACGGCGATGATCTCGTCGTGACCCATGGCGATGCCTGCTTGCCG
ben1_R_NPTII-F2  AAGGCGCGCATGCCCGACGGCGATGATCTCGTCGTGACCCATGGCGATGCCTGCTTGCCG
                 ************************************************************

ben1_O_NPTII-F2  AATATCATGGTGGAAAATGGCCGCTTTTCTGGATTCATCGACTGTGGCCGGCTGGGTGTG
ben1_R_NPTII-F2  AATATCATGGTGGAAAATGGCCGCTTTTCTGGATTCATCGACTGTGGCCGGCTGGGTGTG
                 ************************************************************

ben1_O_NPTII-F2  GCGGACCGCTATCAGGACATAGCGTTGGCTACCCGTGATATTGCTGAAGAGCTTGGCGGC
ben1_R_NPTII-F2  GCGGACCGCTATCAGGACATAGCGTTGGCTACCCGTGATATTGCTGAAGAGCTTGGCGGC
                 ************************************************************

ben1_O_NPTII-F2  GAATGGGCTGACCGCTTCCTCGTGCTTTACGGTATCGCCGCTCCCGATTCGCAGCGCATC
ben1_R_NPTII-F2  GAATGGGCTGACCGCTTCCTCGTGCTTTACGGTATCGCCGCTCCCGATTCGCAGCGCATC
                 ************************************************************

ben1_O_NPTII-F2  GCCTTCTATCGCCTTCTTGACGAGTTCTTCTGAGCGGGACTCTGGGGNN-GAAATGAAAG
ben1_R_NPTII-F2  GCCTTCTATCGCCTTCTTGACGAGTTCTTCTGAGCGGGACTCTGGGGNNCNAAATGAANN
                 ***********************************************   *******
```

Figure S2 ***NPTII* gene sequence did not change in the pre-triple and post-triple *ben1-1* mutants.** The *NPTII* gene was amplified using NPT-Fv1, NPT-Fv2 and NPT-Rv from the pre-triple and post-triple *ben1-1* lines. Primers NPT-Fv1 and NPT-Fv2 were used for sequencing *NPTII* gene (Table S1). The color coded yellow nucleotides show the complete *NPTII* coding region in the sequence.

(A)

```
ben1_O_pBEN1_BST  GGAATTTAATAATAATGTTTGTAATAAATGGATAAAGAATGTGAATTATTTATATAAATG
ben1_T_pBEN1_BST  GGAATTTAATAATAATGTTTGTAATAAATGGATAAAGAATGTGAATTATTTATATAAATG
Col-0_BST         GGAATTTAATAATAATGTTTGTAATAAATGGATAAAGAATGTGAATTATTTATATAAATG
Col-0_no_BST      GGAATCCAATAACAATGTTTGTAATAAACGGACAAAGAATGTGAATTATTCACATAAATG
                  *****  ***** *************** *** ***************** * *******

ben1_O_pBEN1_BST  ATTTGTAAAATGTGAGTATTTGATTATAATGGATATAATAAAATAAAAATTGTAATAAAG
ben1_T_pBEN1_BST  ATTTGTAAAATGTGAGTATTTGATTATAATGGATATAATAAAATAAAAATTGTAATAAAG
Col-0_BST         ATTTGTAAAATGTGAGTATTTGATTATAATGGATATAATAAAATAAAAATTGTAATAAAG
Col-0_no_BST      ATCTGTAAAATGTGAGCATTTGATTATAACGGATATAATAAAACAAAAATTGTAATAAAG
                  ** ************* ************ ************* ****************

ben1_O_pBEN1_BST  AGAGAAGAGGGTAAAATGGGGAATATGTTATGTGGATAGGTGTGAGAGAGTAAATGATGT
ben1_T_pBEN1_BST  AGAGAAGAGGGTAAAATGGGGAATATGTTATGTGGATAGGTGTGAGAGAGTAAATGATGT
Col-0_BST         AGAGAAGAGGGTAAAATGGGGAATATGTTATGTGGATAGGTGTGAGAGAGTAAATGATGT
Col-0_no_BST      AGAGAAGAGGGCAAAACGGGGAACATGTCACGCGGATAGGTGTGAGAGAGTAAATGACGT
                  *********** **** ****** **** * * ************************ **

ben1_O_pBEN1_BST  AGGTTTTGTTATTTAAGAATTTTTTTAGAGAGGTTTTTATATAT-AGNNG----------
ben1_T_pBEN1_BST  AGGTTTTGTTATTTAAGAATTTTTTTAGAGAGGTTTTTATATATTAGAAGAGAGAGAAAG
Col-0_BST         AGGTTTTGTTATTTAAGAATTTTTTTAGAGAGGTTTTTATATATTAGAAGAGAGAGAAAG
Col-0_no_BST      AGGTTCCGTTATTTAAGAACCCTTCTAGAGAGGTCCC-ACACACCAGAAGAG--------
                  *****  ************   ** *********    * * *  **  *

ben1_O_pBEN1_BST  -------------------
ben1_T_pBEN1_BST  AGATAGAGAGAAAATGGTG
Col-0_BST         AGATAGAGAGAAAATGGTG
Col-0_no_BST      -------------------
```

(B)

```
ben1_O_Ex2_BST  GGTGGTGTTTGAGTTTGGTGGGAAGAATGGGTTAGAGGTTGTGATGTTTGTTATTTTTTT
ben1_T_Ex2_BST  GGTGGTGTTTGAGTTTGGTGGGAAGAATGGGTTAGAGGTTGTGATGTTTGTTATTTTTTT
Col-0_BST       GGTGGTGTTTGAGTTTGGTGGGAAGAATGGGTTAGAGGTTGTGATGTTTGTTATTTTTTT
Col-0_no_BST    GGCGGCGCTTGAGTTCGGTGGGAAGAATGGGTTAGAGGTTGTGACGCTCGTTATTCCTCT
                ** ** * ******* **************************** * * ******  * *

ben1_O_Ex2_BST  TGTTGTTGGATTTTTTATATTTTTGTTGTTGTTTTTTTTTGTTTTTATATTTTTTGTTAT
ben1_T_Ex2_BST  TGTTGTTGGATTTTTTATATTTTTGTTGTTGTTTTTTTTTGTTTTTATATTTTTTGTTAT
Col-0_BST       TGTTGTTGGATTTTTTATATTTTTGTTGTTGTTTTTTTTTGTTTTTATATTTTTTGTTAT
Col-0_no_BST    CGTCGTCGGACCTTTTATATCTTCGTCGTTGCCTTCCTCCGTCTTCATATCTCTCGCCAT
                 ** ** ***  ******** ** ** ****  **  *  ** ** **** * * *  **

ben1_O_Ex2_BST  GTTTTTTG/GTATTTAA
ben1_T_Ex2_BST  GTTTTTTG/GTATTTAA
Col-0_BST       GTTTTTTG/GTATTTAA
Col-0_no_BST    GCTCTTTG/GTACTTAA
                * * **** *** ****
```

Figure S3 Genomic DNA in the *BEN1* Promoter and *BEN1* 2nd Exon-Intron junction does not show any methylation in both the pre-triple *ben1-1* and the *bas1-2 sob7-1 ben1-1* triple-mutant lines. (A) *BEN1* promoter DNA was amplified using primers pBEN1-BS-F and pBEN1-BS-R from the bisulfite converted genomic DNA from the Col-0, pre-triple *ben1-1* and the triple-mutant lines. (B) *BEN1* DNA from the 2nd Exon-Intron junction was amplified using primers BEN1-E2-BS-F and BEN1-E2-BS-R from the bisulfite converted genomic DNA from the Col-0, pre-triple *ben1-1* and the triple-mutant lines. BST= bisulfite treatment. ben1_O= pre-triple *ben1-1*, and ben1_T= bas1-*2 sob7-1 ben1-1* triple-mutant.

Bisulfite *BEN1* 2nd exon raw sequences

ACCAGAAGAGAGAGAAAGAGACAGAGAGAAAATGGTGAGAGAAGAACAAGAAGAAGATGACAACAACAACAACAACAACGGAGG
AGGAGAGAGAAAGCTGCTTGTGGCTGATGAAACGGTGCCGTCTTTGTTGGACGAAACGGGGTTGGTTTGTGTAACGGGAGGTTCTG
GTTTCGTTGCTTCTTGGCTCATCATGCGTCTCCTTCAACGTGGCTACTCCGTTCGGGCCACGGTTCGAACCAACTCAGGTTCGAGTTAA
TTAACTTCACTTTCTCTTTACTTATACACTTTGAATCATCATCACTGATTAAGCAAATGCCAATAAAATTATTGTTGTCATATATGGCTGA
TTGGTCCGTTTTAACATAAAGTACATAAATAGAAAGATAGATTATAGTTTTTTTTTTATATATAGAGAGAGATAACATTTAAAGTTGAT
TTTTGGATCGACAATTAAAAATATATATATTTTTATGGATGTGAAAAATTTATTGTACTTGGCTGGAATTATCTGTCAGTTTGTAGGAT
CTTGAAGCTTCATAGCTATGTAAATATGTCATGTTTCCATGCATCTTCTATAGAAAGTCAACCATATATTTTATGATCAATAACTAATAA
GTAATCACGGACCATAAGATATAAATGAAGTTAATAATTCATAAGTTTGTTTATTTTATGCATATTACAGAAGGGAATAAGAAAGATA
TAAGCTACCTAACCGAGCTACCATTTGCATCGGAGAGGCTTCAAATATTCACCGCCGATCTCAACGAACCGGAGAGCTTCAAACCGGC
AATCGAAGGATGCAAAGCCGTATTCCACGTGGCACATCCTATGGACCCAAACAGCAACGAAACAGAAGAGACCGTTACGAAACGTA
CTGTGCAAGGTCTCATGGGGATACTAAAGTCGTGTTTGGACGCTAAAACCGTGAAACGTTTTTTCTACACGTCAAGCGCCGTGACTGT
TTTCTATAGCGGTGGAAACGGCGGTGGAGGAGGCGAAGTAGACGAGAGCGTTTGGAGCGACGTCGAGGTGTTTAGGAATCAAAAG
GAGAAGAGGGTGAGTAGCTCTTACGTCGTGTCGAAAATGGCGGCGGAGACGGCGGCGCTTGAGTTCGGTGGGAAGAATGGGTTAG
AGGTTGTGACGCTCGTTATTCCTCTCGTCGTCGGACCTTTTATATCTTCGTCGTTGCCTTCCTCCGTCTTCATATCTCTCGCCATGCTCTT
TGGTACTTAATTCTCATATCTAATAAGTTTTTTATTTTATTTTTCTCCATGATTAAACTAAAGCTGAAAATTAATAATTTTAAAAACTTAA
CCACTTTGATTACGTATACGTTATATATAGGTATTAATTTTTAAAACTAACCAAACCTTTTATTTCTCAAATAAAGTAAAGTAAATTCAT
AGTACTAGCCTACTAGCTAGCTTGTACTC(**insertion**)TAGTAGTAGTTAAATGGTTTATGGTTGGTTCACCAAGTATCTTGGGAGATCA
TAGCTCGAATTTTCTCAAAAAAAAAATTTTTGAACAAATGCTCGAATTTTCTCATTTATACTTTTGTTTTAAATGAAAAAAATATTCTGC
TCAGGCTTAGTAGATTTATGTTTGGACATAGTGGCTATTGAAAATGTTAGAATTCGAACCAAAATATGTATATTTTATACGAAAAAAC
AGAACTAAAATCCTAATTACTATACGTAACTAATAAAACTCTTTTCTTTTGCTTATAACCTACGTGCGTGGAATTTTTGAGTTATTTAGA
ACGAATTTTGTGTGTGTTAAACTCGTGTAAACATGTGCTAATAATATTCACAAATAGTGTTTTGAGTAATAATCACTATTCACTATAGT
AATATTACGTTTTTTTATGAAGGAAATTACAAGGAGAAGTATTTGTTTGATACTTACAATATGGTGCATATTGATGATGTGGCAAGGG
CGATGATATTTCTATTAGAAAAGCCAGTTGCAAAAGGTAGATATATTTGTTCGTCGGTGGAGATGAAGATCGATGAGGTCTTTGAGT
TTTTGTCTACGAAATTTCCTCAGTTTCAGCTACCTTCAATAGAGTATGTCATTTTTTACTAGTATAATTAATTTCACGTGTAAATATTCTA
TTGTTTTTTTCCAAGAGTTTTTATTATGTTCTTTTTTTGTGTGGGTACGTAAAGTTTGAATAAATATAAAGTAGAGAAGAGGATGGGTC
TTTCATCGAAGAAGCTGAAAAGTGCCGGGTTCGAATTCAAGTATGGAGCCGAGGAAATATTCAGTGGAGCTATAAGGAGCTGTCAA
GCAAGGGGATTTCTTTAAATTCTTATGGGATTTTTGTGTTAAAATTTGTATATGAATTGAAAATATGTTTTTTCTTTATTGGCCATTTCC
GTTTTGTAACATGTTCTAGTTACTTGTTGACGAAAAAAAAAGAAAGTGTTTTGATTCAG

BLUE=Exons; Black=Introns

Primers:

BEN1-E2-BS-F: TTAGGAATTAAAAGGAGAAGAG

BEN1-E2-BS-R: AATTAAATACCAAAAAACATAAC

Replicate 1

>PK_329142-505_11_BS3_E04.ab1/>Col-0_BST
NNNNNNNNNNNNNGNTGTGTTGANTGGTGGTGGAGATGGTGGTGTTTGAGTTTGGTGGGAAGAATGGGTTAGAGGTTGTGA

TGTTTGTTATTTTTTTTGTTGTTGGATTTTTTATATTTTTGTTGTTGTTTTTTTTTGTTTTTATATTTTTTGTTATGTTT
TTTGGTATTTAATTAATCCANCCGCTNTCNNAGCAACTTGNCCCNNNNACCCCCTCGAACGNCTCNNNTCCCCCCCTCCC
CGGCCCCCCAGCTACNAACNCNCTCATCCCNAAGGCTCCANCTAAAACCGTGACTNNCTNCCGTCCCAATTAAGAANGTG
TCTCNNTAAACAAANNTGCNCCCCNNANGACCACCGGTGTCAAACACTTCACTCNTNNAACCTGAAGCCAAANAATCATN
GCCTCNNTTNTGACCCNAANCTCGGAGGGACGCCCNTNATATNNNANCCTTCCCCNCTCCTCTCCCCCNCTTACCCCGAN
ACNNAANCCCGACTGCCCCNTCNNACNTTTCTCAGCCTCCGACAGCGACGCGTATTGATCTCCNCTTTTAACTCCCNCTC
ACNCACTCTTTNGGGTCATNNACCCCNCTNCNCCNNNNNNTNNAGTTTGTAATTATCTCAGGCCCA

>PK_329142-506_12_BS3_F04.ab1/>ben1_O_Ex2_BST
NNNNNNNNNNNNGNTGTGTTGANTGGTGGTGGAGANGGTGGTGTTTGAGTTTGGTGGGAAGAATGGGTTAGAGGTTGTGA
TGTTTGTTATTTTTTTTGTTGTTGGATTTTTTATATTTTTGTTGTTGTTTTTTTTTGTTTTTATATTTTTTGTTATGTTT
TTTGGTATTTAATTANCCAACNCNACNCANNGCCNNTNNACCCCATAACCCCCNCNANNCTNNCTCGTACNCCCCCCNCC
NGNCCCCCTCNCCNNGAACNNCCCGGCCCCGACGGGTCCATCNTANGNCNCGACGGCCCGCGGTGCCAGATANCCAATGG
GCTNCCNAATGAAACNCAAACNCCNCAGGNCCGAGCAGNTCAAAACGNCNNCCATCCCACNGNGAAGGACGAACANCACN
NANNCTCTATCTGGCCNCCCNGTNNGCCNNNGNNCGCTCACNGTCCACCACCCNACCNCAACCCNCCTATNCCNATCAAG
ACACNCCNCNCNACCTGCGNCCTGTCACTCCCAGGCNCCCNACANCGCCGNCTAGNGCGCTCTCCTCNAGCTCCCCCCAC
ATCNNTTCTGAGNTACNCANCCACCTGCNCCTCCCCCNCGATTNCCATNACAANAGCCNAT

>PK_329142-507_13_BS3_G04.ab1/>ben1_R_Ex2_BST
NNNNNNNNNNNGNTGTGTTGANTGGTGGTGGAGATGGTGGTGTTTGAGTTTGGTGGGAAGAATGGGTTAGAGGTTGTGATG
TTTGTTATTTTTTTTGTTGTTGGATTTTTTATATTTTTGTTGTTGTTTTTTTTTGTTTTTATATTTTTTGTTATGTTTTT
TGGTATTTAATANCCAANCGCCTTCACAGCAACCTGGCCCCAAAACCCCTTCGAATGCCTCTCNTCCAACCCCCTCCTCT
CCCCNACNCTANNNNCNACCCNACTCCNGCGGCTCCANCCAACANNCCTTCCNCCNNCTCCCCCATCCTNANCCGGCCTT
CNCCNNNNAACATGATCCNCNCANGGCCNANGGCGNCNAANACCGCATCCCTCCATGGNNGAAGCTTGACCATCACNGAC
TCAATATCTGACTCCCAGCCCGGAGAGAGGGCCGCAACNTCACCCCACCCGCCNNCAACCCCCCATNNCTTCCTCGATAC
NGATCCATGNCTGCGACTTGCCTCTTCCCTTGAGTGCNATCGCGGTGCGTAGTGATCTACTCCTCCNACTTCGCCCTGCA
CACACTCATTTNNTTTACTANNATGATCNNNCTNNTCCCNNCNCNNCNNCNAATTATCNCAAGGCACCATTNNAAAANNN
AA

>PK_329142-508_14_BS3_H04.ab1/>ben1_T_Ex2_BST
NNNNNNNNNNTNGNTGTGTTGANTGGTGGTGGAGATGGTGGTGTTTGAGTTTGGTGGGAAGAATGGGTTAGAGGTTGTGA
TGTTTGTTATTTTTTTTGTTGTTGGATTTTTTATATTTTTGTTGTTGTTTTTTTTTGTTTTTATATTTTTTGTTATGTTT
TTTGGTATTTAANNNNCCCNCCCNNNCNGCCNCCCGNCCCCNANTCCCCNTCGNANGCNNCTCNTCCTCCCCCCCTCCTCN
CCCCACTCTTNCCCCCCCCCGACCCCNACGGCTCCANCNNACGATCNTTCCCCCCNCNCCNCCTTCGCAGANGGGNNAGN
CNNCACCNAACATGATCCCCCCAGGGCCAAAGGCGTCAAAGACAGCTTCCCNGCANGGNNGAAGNATGACAATCACCCAC
TCAATATCGGACCCCCAGCCCGGANAGAGGCCCCCAACTTCACCCCCCCCGCCCNCGACCCCCCTNTNCGTTNCCCGACC
CTGANNCACGGNCCCGACTTGCCTCTTCCATTGAGTGAAATCGCGCTGCGTACCGACCCACTCCTCACACCTCNCCCGCA
CACTACNTCCGNTTTACCAANNNNATCCGTCNNNCCCCTCANNTCNNCCNATTATCACCAGGCCCATTNTNANNNNNNNN
N

>PK_329142-519_11_BS4_E06.ab1/>Col-0_BST
NNNNNNNNNANNNAAAATATAAAAAATCCAACAACAAAAAAAATAACAAACATCACAACCTCTAACCCATTCTTCCCACC
AAACTCAAACACCACCATCTCCACCACCATTTTCAACACAACATAAAAACTACTCACCCTCTTCTCCTTTTAATTCCTAA
AANNGGNNGNNNACNNNANGGNNNTGNNTNNNCNNATACTNCTCCGGGNGAGCGAGACTACGCTATNTGNTGAGAGGAGT
TGTTNGAGCGAAATNCCATNANAAAAAGGACAT

>PK_329142-520_12_BS4_F06.ab1/>ben1_O_Ex2_BST
NNNNNNNNNANNAAAAATATAAAAAATCCAACAACAAAAAAAATAACAAACATCACAACCTCTAACCCATTCTTCCCACC
AAACTCAAACACCACCATCTCCACCACCATTTTCAACACAACATAAAAACTACTCACCCTCTTCTCCTTTTAATTCCTAA
AAANGTANGNNGAGGGCTNGNGNGGTCAGTGTGACCATCTTCTTCTGGAGGACGTGCCAAAANTTTACTCCTAANCNAGG
TGATTTNCGAGGGCCACCCCATGGNGAANCNCATCGAGTTGAGGGGCATCCTCTTCNAGGAGGACAGAAANNTCN

>PK_329142-521_13_BS4_G06.ab1/>ben1_R_Ex2_BST
NNNNNNNNNNNANCANNNAAAATATAAAAAATCCAACAACAAAAAAAATAACAAACATCACAACCTCTAACCCATTCTTCC
CACCAAACTCAAACACCACCATCTCCACCACCATTTTCAACACAACATAAAAACTACTCACCCTCTTCTCCTTTTAATTC
CTAAAANNNNGANGGGNGTANGGGNNGANCTTGTCCTACATNCNTCGGGCNCCTGCTNTGACTCCANCNNCTGGGCCCCG
TGTTTTCCGGGCNATCCCATGNACAATAGNNCACCGAGTACCGGAATGTTCCCGTCCCANGCTTGAACCNGTCNGATCNC

ATTGGTGAANACNANATTACGAACANTCTCNAGTCANCTATTTTNGTGTTACGGACTAANAGNATCANNGTANNCTTCTA
AATTGTCCACNACAACCNAGNANGTTCCNCCACGGTCGCTCATCGGCCGCCGGCTACCCTCANNCTGCATACAGACTGAT
TGGATGACNCAACATCCTNNNTTTGATGAAGGCGATATTCNAGATTAGNTACNTAATGCTNANTCTTAANTCTCTCANAC
TGNTGCATTGGGGACCNTAANGCNCATGCTCTCTNTNCCTAGNCGAACCNACTATAACCTTCNTCTGN

>PK_329142-522_14_BS4_H06.ab1/>ben1_T_Ex2_BST
NNNNNNNNNNNNNNNANNNAAAAATATAAAAAATCCAACAACAAAAAAAATAACAAACATCACAACCTCTAACCCATTCT
TCCCACCAAACTCAAACACCACCATCTCCACCACCATTTTCAACACAACATAAAAACTACTCACCCTCTTCTCCTTTTAA
TTCCTAAATNNGCCNGNGGGTTNGNGGGGNGGGGNCGGTCTTCNTCGGGATGGGNCNGACTNACNNGCTGTGAANGTGGT
TTCGGGNNATACNCCCNGGAGAACCACATCGCTCTGAGGGGCATCCTCTTGGAGGANANCGAAACGTCCTGGGGGAGNNG
TGGAGTACAANNNGANNNACCACAGCNTTANCNTGNTGGCGGACAAGAATAAGGGTNGCATTTTGTTGAACTTCATGATC
CACCTGTACTCGAAGGCTGGTGTTGTAGCTNANCNACCCNTGCCTACAGCTCAGCCCNNGGANNGACCGGCCCTGGTGGC
GGCCCGAACTNNTTNCCTGATC

```
ben1_O_Ex2_BST  GGTGGTGTTTGAGTTTGGTGGGAAGAATGGGTTAGAGGTTGTGATGTTTGTTATTTTTTT
ben1_T_Ex2_BST  GGTGGTGTTTGAGTTTGGTGGGAAGAATGGGTTAGAGGTTGTGATGTTTGTTATTTTTTT
Col-0_BST       GGTGGTGTTTGAGTTTGGTGGGAAGAATGGGTTAGAGGTTGTGATGTTTGTTATTTTTTT
Col-0_no_BST    GGCGGCGCTTGAGTTCGGTGGGAAGAATGGGTTAGAGGTTGTGACGCTCGTTATTCCTCT
                ** ** * ******* **************************** * * ******  * *

ben1_O_Ex2_BST  TGTTGTTGGATTTTTTATATTTTTGTTGTTGTTTTTTTTTGTTTTTATATTTTTTGTTAT
ben1_T_Ex2_BST  TGTTGTTGGATTTTTTATATTTTTGTTGTTGTTTTTTTTTGTTTTTATATTTTTTGTTAT
Col-0_BST       TGTTGTTGGATTTTTTATATTTTTGTTGTTGTTTTTTTTTGTTTTTATATTTTTTGTTAT
Col-0_no_BST    CGTCGTCGGACCTTTTATATCTTCGTCGTTGCCTTCCTCCGTCTTCATATCTCTCGCCAT
                 ** ** ***  ******** ** ** ****  **  *  ** ** **** * * *  **

ben1_O_Ex2_BST  GTTTTTTG/GTATTTAA
ben1_T_Ex2_BST  GTTTTTTG/GTATTTAA
Col-0_BST       GTTTTTTG/GTATTTAA
Col-0_no_BST    GCTCTTTG/GTACTTAA
                * * **** *** ****
```

Replicate 2

>PK_329142-511_17_BS3_C05.ab1/>ben1_O_Ex2_BST
NNNNNNNNNNNNGNTGTGTTGANTGGTGGTGGAGATGGTGGTGTTTGAGTTTGGTGGGAAGAATGGGTTAGAGGTTGTGA
TGTTTGTTATTTTTTTTTGTTGTTGGATTTTTTATATTTTTGTTGTTGTTTTTTTTTTTATTTTATATTTTTTGTTATGTTT
TTTGGTATTTAATTANGAANCNTATGGACTGGACCCCNACCCNCTTCTCCGAANCGCNNTCCCNNCNTNNTCTGNTGAAC
CTACTTCTCTGCGGCANCCACCTNATTAANCCCNCCNNNTCNATATGCCCCTNACCCNCCTNCAAANACTATANCCTCNG
GGNGCCCCNGGTCCCTNTCCTNNTGCCNGNCCNCCACTTNTCCNCNNCCNCNTACTNNNAATATGCNCTNNTGGTATATT
ACTAGNATATNACCCNNNNTATTNNTGCNT

>PK_329142-512_18_BS3_D05.ab1/>ben1_R_Ex2_BST
NNNNNNNNNNNNTGTGTTGANTGGTGGTGGAGATGGTGGTGTTTGAGTTTGGTGGGAAGAATGGGTTAGAGGTTGTGATG
TTTGTTATTTTTTTTGTTGTTGGATTTTTTATATTTTTGTTGTTGTTTTTTTTTTGTTTTTATATTTTTTGTTATGTTTTT
TGGTATTTAATAAATACCNNNNNTTTAAGGATGTTTTTGAAACGGTCTNCTTCGGGTCGGGGTCCCGCCGCTGCTTNGTA
AACGTAGTTNGTNNACCNCNCCTCCCTNGNACCACGCCANATCNACNTGTTCCTNACTCTCCACCAAGNN

>PK_329142-513_19_BS3_E05.ab1/>ben1_T_Ex2_BST
NNNNNNNNNNNTGNTGTGTTGANTGGTGGTGGAGATGGTGGTGTTTGAGTTTGGTGGGAAGAATGGGTTAGAGGTTGTGA
TGTTTGTTATTTTTTTTTGTTGTTGGATTTTTTATATTTTTGTTGTTGTTTTTTTTTTGTTTTTATATTTTTTGTTATGTTT
TTTGGTATTTAATTAATACCCACCAATTTCGGANNNNNNTTNNCNNTCTTCTTCNGGGNGGNGCGANTGCTCTNCTTGTT

GTGNTTTTTTTTTGAGTGCCACCCCCTTTTTAATACGACATCTCTTTNTGTATCGNANTCGTCTTCTTNTTGN

>PK_329142-514_20_BS3_F05.ab1/>ben1_T_Ex2_BST
NNNNNNNNNNGTTGTGTTGANTGGTGGTGGAGATGGTGGTGTTTGAGTTTGGTGGGAAGAATGGGTTAGAGGTTGTGATG
TTTGTTATTTTTTTTGTTGTTGGATTTTTTATATTTTTGTTGTTGTTTTTTTTTGTTTTTATATTTTTTGTTATGTTTTT
TGGTATTTAATAANNNCCNNCCGCTACGTCCAGGAGCNNACCNTCTNTCTCCGGACCACGGCGACTCCGCTACCTGCTCC
GACGTGTTNTTCGAGGGCCACACCCTGTTGAACCGCNTCNCGCTGAATGGTACCNACTTCNNNCATGACNGCNACATCCT
GGGGCTAANNTGGANTNNCCTACNACAGCCACAANNTCTATATCCTGTTTCNATTCNNTGAAGAACAGCANCNCNGTGAA
CTTCTNNATCCNCCNCNCNCNNCNCTAAGGTTCCNTCTNANNNNNNACCNCTTNCTNCNCAACNCTCCCCTCGTNGACGG
NCCCGTGNTGCTGCCCGCAACCACTATCTGANCACCCCTNCCTCCCTGAGTTNTATTTTANNNATN

>PK_329142-525_17_BS4_C07.ab1/>ben1_O_Ex2_BST
NNNNNNNNNNNNNNNNNNNAAANTATAANANATCCAACANNNNAAAAAATAACAAACTTCCCATCCTCTAACCCATTCTTC
CCACCAGACTCAAACACCACCATCTCCACCACCATTTTCAACACAACATAAAAACTACTCACCCTCTTCTCCTTTTAATT
CCTAAAANGTNNNNNCCGNCNTGGNCNGNNNCGGCATGNATATNNTGCGGGTGGNGCTGACCGTGCTGTNCTGNNTGANC
GTTTNGTTGATCTGCNCCCTGGCCATGNNGAANGNGACCTTGTTCNACTGTAACCNTGTNCTGCTGCNACAACCGNNNNG
GGACCCACNGTGTGTGAAATNGCTGGTGCATAGNGCCGGCTAAATGCACTNCTCGTTGGNNGACNACTCCCATGTTGGGC
CCCAAGCCACCNNTGCTTGATGACTTCTCNCCGTGCTCGCCCTGCTGGTNGCTCTGTTTGNNCTGCTGGTTGANTGNCCG
GNCCNNCACCGGTACCNACTTGGNACAAACAGACNGNATATGACGNCANTCTCTTACANGCTCCATTNGTTNNGAATTGN
GTCTTTNTCTNNCTNTCTCTCAACCGTGTTNTACTNNCCATTCGCTCGTGCNGGGCTCCTGCCACCTCGNGATGTCGGAC
TNCNANAATAATGTANTTTANCNNTGCCTATTNATNTCCTNTGCGNTAAACCNCCTGCTACGCCCAGGTGG

>PK_329142-526_18_BS4_D07.ab1/>ben1_R_Ex2_BST
NNNNNNNNNNNANAANAAAAATATAAAAAAATCCAACAACAAAAAAAAATAACAAACATCACAACCTCTAACCCATTCTTCCC
ACCAAACTCAAACACCACCATCTCCACCACCATTTTCAACACAACATAAAAACTACTCACCCTCTTCTCCTTTTAATTCC
TAAAANGTAAAGGGTGATGGNNTCNGCCGGCANGGCCATCCTNTGCGAGAGGCGGACCCTGTGCTGTTGAGTGAACGAAC
TGNTGAGCTGCGCCCTGCCTNNTAGGNATGTGANCNCCTTCNNNGCCAACNCNNNGNNCTAGTNCCATAACNNCATCNAG
CCCCTGNGGATGAGCTNNGNGGGGGGAGCANCGNCCCTATTANCACTANTCGTTAGATCANGACTCGNATGTGGTACGAG
ATTACACCGCTGCTTGAGGGCTGCCCATGATCNGCGTCCTGGAGGCCCTGCTNNATCGTTGNTGTACATANNAGGCCTCT
CNTGCACCGGCTGCTGANAAGAAACAGGCTCCCTGGACAGGCTGGNACTAATGNCCAGGATCGTGTTCCTGATCAAATGG
ATGTTTATCTGGCTCTCTCTGTGCTGTCCTGCTCACTCCATTCGCGCGNGCTTGGACNCCCCCNTGNTGGNAGGNNGTNN
NAAAGNAAACTGNNNNCNANCCNTGCCTGNNCTGNTCCNGGGNCNNNATGCTGCTGNTNNNGNNAGN

>PK_329142-527_19_BS4_E07.ab1/>ben1_T_Ex2_BST
NNNNNNNNNNNNNNNNAAAAATATAAAAAAATCCAACAACAAAAAAAAATAACAAACATCACAACCTCTAACCCATTCTTCCC
ACCAAACTCAAACACCACCATCTCCACCACCATTTTCAACACAACATAAAAACTACTCACCCTCTTCTCCTTTTAATTCC
TAAAANGGGTAGAGCGNCCATGATCTCNGCCGGCGGGNNGATCCTGGGCGGGGTGGGGACCCTGCGCGGCTGGGTGAACG
GGCTGGGGTGATGCCCCCTGCCGNTGTGGTAGGTNACCGCCTTGTGTGCCTTAATTATCGGGGTGGATGGNTNGGTGTGG
GAAGGCCAGNGGATGAGNTGTGCGGTGCNCCGCNCCGGNCAGAGTCCTNGCANGGTGNGGACAGCAGCNNGGATGTGCCC
CTGGACGCGCAGGCCGCAAGAGGACTGCGCGTGATCACCCNGCTGGTGGCCCTGTTCNNNCTGATGGTGTNCATGGCNGC
NCTCATNTGCACCACCAGNGCGCAGGACAAAGACAGCCAGGCCAGGCTGGCGCTGACCACCGGCCTCGTGTTCGTGATCN
NNGGGTGCTGAACNTGATCACGCTGAGCTGGGCCGCCNNCTCCATGATCACGTACTTCTNTATTGCCGTGTTGGNNTANN
GNAGNANNANNAAGNNGNNGTCNTACACGTNNCTGTGCTGGTGCNCGGTCGTCAGNTGNTGCTNNNGNNTGGTTGGNCNN
NNGTTGNNTCGNNNTTNGCANNANGNNNNNGCCCANCANACNGGCNCCATCTACACGANCANGTCGCAGAGCCGCCTNNN
GANGNCCCANGGNANNNNGNNNNGGNTNNN

>PK_329142-528_20_BS4_F07.ab1/>ben1_T_Ex2_BST
NNNNNNNNNCNANNANNAAAATATAAAAAAATCCAACAACAAAAAAAAATAACAAACATCACAACCTCTAACCCATTCTTCC
CACCAAACTCAAACACCACCATCTCCACCACCATTTTCAACACAACATAAAAACTACTCACCCTCTTCTCCTTTTAATTC
CTAAAANNTGGCTGGGNTGATNTGGNNGGTGACGGAATGGTCTGCGCCGGCATGCCCATCCTGGGCGTGGTGGTGANCCT
GCTGGGCTGGGCGAACGGCCTGGTGAGCTGCGCCTTGTCTATGTGGAATGTGACCTCCTTCNTCNGCAACAACCTCNTGG
TGGCCCAGGTGGTGTGGGAGGGCCTGTGGATGANCTGNGNGGTGCANANCACCGGCGGATGCANTGTCNNGNGTAGGACC
CTCTGCTGGNNCTGCCCNAGGACCTGNANGANCAACAGCCCTGTGGNTGATCNNCCTGCTGGTGGNCCTGTTCGGCCTGN
TGGTGTACCTGGACGGCGCTAGGTGCAACAANTGCGTGCAGGATAACGATATCCACGNCAANCTGGTGGCNACTGGCGCN
NTCCTGTTTCGTGATCACGGCGAGCTGACCCNCATCNCNGTGNNCTNGAGCNNCGANCNTGCCNTCTGGGAGTCCTGNNC
ANGCTGNNGGTGNAGNCNCACACCNANGCANNTNNGCTGNNCGNNTGTACTTGNNNNGCANCTCNANCGNNNN

File S2

Bisulfite sequences of the *BEN1* promoter

ccattccatctcattcacgcgttttattttctctcattcatattaattatatattaatcacacaatcctaataatatcccttaatctatgcct
agaacatgcatggtattataattaatacattcgacaccactagaagccaaaagtatagtagtaaaaaaaagaagaagaagctggtttgtacgt
aagaatgaaaataacggaatccaataacaatgtttgtaataaacggacaaagaatgtgaattattcacataaatgatctgtaaaatgtgagca
tttgattataacggatataataaaacaaaaattgtaataaagagagaagagggcaaaacggggaacatgtcacgcggataggtgtgagagagt
aaatgacgtaggttccgttatttaagaacccttctagagaggttcccacacaccagaagagagagaaagagacagagagaaaATGGTGAGAGA
AGAACAAGAAGAAGATGACAACAACAACAACAAC

Primers:

pBEN1-BS-Fv: 5'-GTATAGTAGTAAAAAAAAGAAGAAGAAG-3'

pBEN1-BS-Rv: 5'-CTTRTTCTTCTCTCACCATTTTCTCTCT-3'

(RC=reverse compliment)

Replicate 1

>PK_328409-528_5-R_Primer-D_A02.ab1/>Col-0_no_BST
NNNNNNNNNNNCTCTTCTGGTGTGTGGGACCTCTCTAGAAGGGTTCTTAAATAACGGAACCTACGTCATTTACTCTCTCA
CACCTATCCGCGTGACATGTTCCCCGTTTTGCCCTCTTCTCTCTTTATTACAATTTTTGTTTTATTATATCCGTTATAAT
CAAATGCTCACATTTTACAGATCATTTATGTGAATAATTCACATTCTTTGTCCGTTTATTACAAACATTGTTATTGGATT
CCGTTATTTTCATTCTTACGTACAAACCAGCTTCTTCTTCTTTTTTTTACTACTATAN

>RC_328409-528_5-R_Primer-D_A02.ab1/>Col-0_no_BST
NTATAGTAGTAAAAAAAAGAAGAAGAAGCTGGTTTGTACGTAAGAATGAAAATAACGGAATCCAATAACAATGTTTGTAATAAAC
GGACAAAGAATGTGAATTATTCACATAAATGATCTGTAAAATGTGAGCATTTGATTATAACGGATATAATAAAACAAAAATTGTA
ATAAAGAGAGAAGAGGGCAAAACGGGGAACATGTCACGCGGATAGGTGTGAGAGAGTAAATGACGTAGGTTCCGTTATTTAAGAA
CCCTTCTAGAGAGGTCCCACACACCAGAAGAG

>PK_328409-529_6-F_Primer-C_B02.ab1/>Col-0_BST
NNNNNNNNNNGTAGATGANATAATGGAATTTAATAATAATGTTTGTAATAAATGGATAAAGAATGTGAATTATTTATATA
AATGATTTGTAAAATGTGAGTATTTGATTATAATGGATATAATAAAATAAAAATTGTAATAAAGAGAGAAGAGGGTAAAA
TGGGGAATATGTTATGTGGATAGGTGTGAGAGAGTAAATGATGTAGGTTTTGTTATTTAAGAATTTTTTTAGAGAGGTTT
TTATATATTAGAAGAGAGAGAAAGAGATAGAGAGAAAATGGTGAGAGAAGAACAAGACNNCCTNCNNCCTTCCCTCCCCC
CCACCTATCCACATACCATTTTCCCCCTTTCACCCCCTTCTCTCTTTATNCAAATCTTTTTTGCTCATCNCCCNACNAAT
CAAATACCCCATTTTTCACATCNNTCTTCTAAATAATTCCATTCTTTATCCATTTTTCCCTCACATTAGGCTAATTCCTC
ACTNTTTTCTTTCCTTATACAAACNNCTTCTTCTTCTTTTNGGGTACNCNANACCAGCTNTTGCTNCCTTTAGTNNN

>PK_328409-530_6-R_Primer-D_C02.ab1/>Col-0_BST
NNNNNNNNNNCTTCNATATATATAAAAACCTCTCTAAAAAAATTCTTAAATAACAAAACCTACATCATTTACTCTCTCACAC
CTATCCACATAACATATTCCCCATTTTACCCTCTTCTCTCTTTATTACAATTTTTATTTTATTATATCCATTATAATCAA
ATACTCACATTTTACAAATCATTTATATAAATAATTCACATTCTTTATCCATTTATTACAAACATTATTATTAAATTCCA
TTATTTTCATTCTTACATACAAACCAACTTCTTCTTCTTTTTTTTACTACTATAN

>PK_328409-531_7-F_Primer-C_D02.ab1/>ben1_O_pBEN1_BST
NNNNNNNNNNNNNTGTNNATGANATAANGGAATTTAATAATAATGTTTGTAATAAATGGATAAAGAATGTGAATTATTTAT
ATAAATGATTTGTAAAATGTGAGTATTTGATTATAATGGATATAATAAAATAAAAATTGTAATAAAGAGAGAAGAGGGTA
AAATGGGGAATATGTTATGTGGATAGGTGTGAGAGAGTAAATGATGTAGGTTTTGTTATTTAAGAATTTTTTTNNAGAGG
TTCTTATATATTAGACNACAGAGCAAGAG

>PK_328409-532_7-R_Primer-D_E02.ab1/>ben1_O_pBEN1_BST
NNNNNNNNNNNNNNCNNCTATATATAAAAACCTCTCTAAAAAAATTCTTAAATAACAAAACCTACATCATTTACTCTCTC

ACACCTATCCACATAACATATTCCCCATTTTACCCTCTTCTCTCTTTATTACAATTTTTATTTTATTATATCCATTATAA
TCAAATACTCACATTTTACAAATCATTTATATAAATAATTCACATTCTTTATCCATTTATTACAAACATTATTATTAAAT
TCCATTATTTTCATTCTTACATACAAACCAACTTCTTCTTCTTTTTTTTACTACTATACANNN

>PK_328409-533_8-F_Primer-C_F02.ab1/>ben1_R_pBEN1_BST
NNNNNTNNGNNTGTANGATGANNTAATGGAATTTAATAATAATGTTTGTAATAAATGGATAAAGAATGTGAATTATTTAT
ATAAATGATTTGTAAAATGTGAGTATTTGATTATAATGGATATAATAAAATAAAAATTGTAATAAAGAGAGAAGAGGGTA
AAATGGGGAATATGTTATGTGGATAGGTGTGAGAGAGTAAATGATGTAGGTTTTGTTATTTAAGAATTTTTTTAGAGAGG
TTTTTATATATTAGAAGAGAGAGAAAGAGATAGAGAGAAAATGGTGAGAGAAGAACAAGAANANANACATNTTTACTCTC
TCACACCTATCCACATAANTATTCCCATTTTACCCTCTTCTCTCTTTATTAAATTTTTATTNNN

>PK_328409-534_8-R_Primer-D_G02.ab1/>ben1_O_pBEN1_BST
NNNNNNNNNNNCTTCNATATATAAAAACCTCTCTAAAAAAATTCTTAAATAACAAAACCTACATCATTTACTCTCTCACA
CCTATCCACATAACATATTCCCCATTTTACCCTCTTCTCTCTTTATTACAATTTTTATTTTATTATATCCATTATAATCA
AATACTCACATTTTACAAATCATTTATATAAATAATTCACATTCTTTATCCATTTATTACAAACATTATTATTAAATTCC
ATTATTTTCATTCTTACATACAAACCAACTTCTTCTTCTTTTTTTTACTACTATAN

>PK_328409-535_9-F_Primer-C_H02.ab1/>ben1_T_pBEN1_BST
NNNNNNNNNNGTAGATGANATAATGGAATTTAATAATAATGTTTGTAATAAATGGATAAAGAATGTGAATTATTTATATA
AATGATTTGTAAAATGTGAGTATTTGATTATAATGGATATAATAAAATAAAAATTGTAATAAAGAGAGAAGAGGGTAAAA
TGGGGAATATGTTATGTGGATAGGTGTGAGAGAGTAAATGATGTAGGTTTTGTTATTTAAGAATTTTTTTAGAGAGGTTT
TTATATATTAGAAGAGAGAGAAAGAGATAGAGAGAAAATGGTGAGAGAAGAACAAGAAAACCTACATCATTTACTCTCTC
ACACCTATCCACATAACATATTCCCCATTTTACCCTCTTCTCTCTTTANNNANNTTTTATTTTATTATATCCATTANAAT
CAATACTCCATTTTCAAATCATTNATATAAATAATTCCNTTCNTTNNNCNNNGTTCCANACATTNTTNTTNNNT

>PK_328409-536_9-R_Primer-D_A03.ab1/>ben1_T_pBEN1_BST
NNNNNNNNNNNNNNNNTCTNNNNNNTATAAAAACCTCTCTAAAAAAATTCTTAAATAACAAAACCTACATCATTTACTCTC
TCACACCTATCCACATAACATATTCCCCATTTTACCCTCTTCTCTCTTTATTACAATTTTTATTTTATTATATCCATTAT
AATCAAATACTCACATTTTACAAATCATTTATATAAATAATTCACATTCTTTATCCATTTATTACAAACATTATTATTAA
ATTCCATTATTTTCATTCTTACATACAAACCAACTTCTTCTTCTTTTTTTTACTACTATACCANNGNNNNGGCCGTGGNN
NGAGCGTNCGGCCTTTTTGNTGTTGCGTNCGGTCTGTTTTTTGTAACAACAATNGCGCCTANGCCATCTTCNATTTTCGG
NGNCCTTTANCTTGCCACGNCCCAGAGCNCCNCAGTAGCCGCCGNATCCNCCNNAAANNGGGNAGTCCACCCANNAGATG
AAGCTGATCGTTTCCCNCNACCCCNACGGGGGGCNCGNTNCCCAACTTTTGTTCCTGTTTNNGAGAGCNCANNTNANNAA
TTGAGACTGNNNNGGNCTGGCTGGTTCCCNGTTNAGATTNTAAACCGCACACTGTTCCCNATATCTCANGACCGGNAGNA
TATGNNTNAGCCCGNCGNANNNNATNNTTANNTAANTGNNCTAGNTTNTGCGGTTANTNCCACTTTCCNNACNNNGAGTC
CTTNNNNCNNNCTNCATTTTCTAACNTCTANCAATGNAGCNNGTTNTTGNAATACCTAAT

Sequence alignment:

```
ben1_O_pBEN1_BST  GGAATTTAATAATAATGTTTGTAATAAATGGATAAAGAATGTGAATTATTTATATAAATG
ben1_T_pBEN1_BST  GGAATTTAATAATAATGTTTGTAATAAATGGATAAAGAATGTGAATTATTTATATAAATG
Col-0_BST         GGAATTTAATAATAATGTTTGTAATAAATGGATAAAGAATGTGAATTATTTATATAAATG
Col-0_no_BST      GGAATCCAATAACAATGTTTGTAATAAACGGACAAAGAATGTGAATTATTCACATAAATG
                  *****  ***** *************** *** ***************** * *******

ben1_O_pBEN1_BST  ATTTGTAAAATGTGAGTATTTGATTATAATGGATATAATAAAATAAAAATTGTAATAAAG
ben1_T_pBEN1_BST  ATTTGTAAAATGTGAGTATTTGATTATAATGGATATAATAAAATAAAAATTGTAATAAAG
Col-0_BST         ATTTGTAAAATGTGAGTATTTGATTATAATGGATATAATAAAATAAAAATTGTAATAAAG
Col-0_no_BST      ATCTGTAAAATGTGAGCATTTGATTATAACGGATATAATAAAACAAAAATTGTAATAAAG
                  ** ************* ************ ************* ****************

ben1_O_pBEN1_BST  AGAGAAGAGGGTAAAATGGGGAATATGTTATGTGGATAGGTGTGAGAGAGTAAATGATGT
ben1_T_pBEN1_BST  AGAGAAGAGGGTAAAATGGGGAATATGTTATGTGGATAGGTGTGAGAGAGTAAATGATGT
Col-0_BST         AGAGAAGAGGGTAAAATGGGGAATATGTTATGTGGATAGGTGTGAGAGAGTAAATGATGT
Col-0_no_BST      AGAGAAGAGGGCAAAACGGGGAACATGTCACGCGGATAGGTGTGAGAGAGTAAATGACGT
                  *********** **** ****** **** * * ************************ **
```

```
ben1_O_pBEN1_BST  AGGTTTTGTTATTTAAGAATTTTTTTAGAGAGGTTTTTATATAT-AGNNG----------
ben1_T_pBEN1_BST  AGGTTTTGTTATTTAAGAATTTTTTTAGAGAGGTTTTTATATATTAGAAGAGAGAGAAAG
Col-0_BST         AGGTTTTGTTATTTAAGAATTTTTTTAGAGAGGTTTTTATATATTAGAAGAGAGAGAAAG
Col-0_no_BST      AGGTTCCGTTATTTAAGAACCCTTCTAGAGAGGTCCC-ACACACCAGAAGAG--------
                  *****  ************    ** *********     * * *   **   *

ben1_O_pBEN1_BST  -------------------
ben1_T_pBEN1_BST  AGATAGAGAGAAAATGGTG
Col-0_BST         AGATAGAGAGAAAATGGTG
Col-0_no_BST      -------------------
```

Replicate 2

>PK_329142-503_7_pBEN1-Fv_C04.ab1/>ben1_O_pBEN1_BST
NNNNNNNNNNNNNNNNNATGANATAATGGAATTTAATAATAATGTTTGTAATAAATGGATAAAGAATGTGAATTATTTAT
ATAAATGATTTGTAAAATGTGAGTATTTGATTATAATGGATATAATAAAATAAAAATTGTAATAAAGAGAGAAGAGGGTA
AAATGGGGAATATGTTATGTGGATAGGTGTGAGAGACTAAATGATGTAAGTTTTGTNANTCCTNCNTTTTGTCCNCCCCC
CTCTCCCCCCTCTNACCCCNCCCNCNCCNCTACAGAGGNCCNCCNCCTACCNCNNNCCCGACCNTCCCNCCCCAGTACCN
GTCNNATCCCCTNGACAANNCCCACTCCCCCNACGCCCTCCGGACCCNNCCCTCCNNNNNCTCNCTCNNANANCCATGCT
NCNCTCTNNNTGTCNNCCNCCCCCNNCNCCGAGCCACCCTCCTCCACCNCGCCCCCCCGCCCCCCCCCCCTCTGCCNCTTT
CCCATCGCNNANTGCCTGATGCCACATCCNTCTNNGCCACCCTCCAACTTCGCCGCGTCGTGCTCAACCNCCNCTCNCCC
CNCCCCCACCCTCTCTCNNGCCNCCTCCCACCCCCCNTCCCTNNCCAANNN

>PK_329142-504_8_pBEN1-Fv_D04.ab1/>ben1_R_pBEN1_BST
NNNNNNNNNNNNGTAGATGANATAATGGAATTTAATAATAATGTTTGTAATAAATGGATAAAGAATGTGAATTATTTATA
TAAATGATTTGTAAAATGTGAGTATTTGATTATAATGGATATAATAAAATAAAAATTGTAATAAAGAGAGAAGAGGGTAA
AATGGGGAATATGTTATGTGGATAGGTGTGAGAGAGTAAATGATGTAGGTTTTGTTATTTAAGAATTTTTTTAGAGAGGT
TTTTATATATTAGAAGAGAGAGAAAGAGATAGAGAGAAAATGGTGAGAGAAGAACAAGANNNCCCCCTNANNCCCGTCGG
GTCTCCTTANCNNATACCACCTCNCCGATGCCCCNCNGCGCCNTGANCNNGTCNNCTCTCNGAGNGGCNTGCNCCNCNNC
GCCCCNNTTGNGNCCNNNNACTCCGAGCNNNNNNCGCCCNCCCCGCCCCCCCNNCCCCCACCCTCTGCCTCGNATCTGAC
TCCTGANTGCCGGTGGCCAACCTCCCNCGAATGACNCCCTCCGACNGCGGCGCGTCGTGCTCGCCNCCACTCNCCCCCCC
CCGACACCNCNCTGGGNTCCGNCNCCCCCTGCCCCCCCNCN

>PK_329142-517_7_pBEN1-Rv_C06.ab1/>ben1_O_pBEN1_BST
NNNNNNNNNNNNNNNNNNNNNAAAACCTCTCTAAAAAAATTCTTAAATAACAAAACCTACATCATTTACTCTCTCACACC
TATCCACATAACATATTCCCCATTTTACCCTCTTCTCTCTTTATTACAATTTTTATTTTATTATATCCATTATAATCAAA
TACTCACATTTTACAAATCATTTATATAAATAGGTCACATTCTTTATCCATTTATTACAAACATTATTATTAAATTCCAT
TATTTTCATTCTTACATACAAACCAACTTCTTCTTCTTTTTTTTACTACTATACAGGNGCNGNNGGTGGTCNTGTAGGGN
CGGGGTGGCTGNCTGNNGCNGNTNGNNNNGGTGCGTACATTTACTAGGNNCCTANCNNNNGCGAGNCGCTGGCNGCGTAG
TCCTGNAGCGGCNGCGGCCCGGNGACCGCTGGTGGGCCGCGNCCTCGAGGGGGNTGTANANGGCCCNGANGATGANNGNN
CGGNCGNGCNGCNGNGGATCAGGNTNAACNNGCCNNTGATCANGGNNGGGATGNCGNNGGCCATCGCGNTN

>PK_329142-518_8_pBEN1-Rv_D06.ab1/>ben1_R_pBEN1_BST
NNGNGNNGNNNNNNNNNANGNNNNNNNNNNTGCTNNAAAAAGGCTTAAATAACAAAACCTACATNATTTGNTCTCTNNNN
CCTATCCACATAACATATTCCCCATTTTACCCTCTTCTCTCTTTATTACAATTTTTATTTTATTATATCCATTATAATCA
AATGCTCACATTTTACAGATCATTTATATAGATAGTTNACATTCTTTATCCATTTATTACAAACATTATTATTAAATTCC
ATTATTTTCATTCTTACATACAAACCAACTTCTTCTTCTTTTTTTTACTACTATACN

File S3

Bisulfite pNOS raw sequences

GTTTACCCGCCAATATATCCTGTCAAACACTGATAGTTTAAACTGAAGGCGGGAAACGACAATCTGATCATGAGCGGAGAATTAAGGGAGTCACGT
TATGACCCCCGCCGATGACGCGGGACAAGCCGTTTTACGTTTGGAACTGACAGAACCGCAACGTTGAAGGAGCCACTCAG**CCGCGG**GTTTCTGGA
GTTTAATGAGCTAAGCACATACGTCAGAAACCATTATTGCGCGTTCAAAAGTCGCCTAAGGTCACTATCAGCTAGCAAATATTTCTTGTCAAAAATG
CTCCACTGACGTTCCATAAATTCCCCTCGGTATCCAATTAGAGTCTCATATTCACTCTCAATCCAAATAATCTGCACCGGATCTGGATCGTTTCGCATG
ATTGAACAAGATGGATTGCACGCAGGTTCTCCGGCCGCTTGGGTGGAGAGGCTATTCGGCTATGACTGGGCACAACAGACAATCGGCTGCTCTGA
TGCCGCCGTGTTCCGGCTGTCAGCGCAGGGGCGCCCGGTTCTTTTTGTCAAGACCGACCTGTCCGGTGCCCTGAATGAACTGCAGGACGAGGCAG
CGCGGCTATCGTGGCTGGCCACGACGGGCGTTCCTTGCGCAGCTGTGCTCGACGTTGTCACTGAAGCGGGAAGGGACTGGCTGCTATTGGGCGAA
GTGCCGGGGCAGGATCTCCTGTCATCTCACCTTGCTCCTGCCGAGAAAGTATCCATCATGGCTGATGCAATGCGGCGGCTGCATACGCTTGATCCG
GCTACCTGCCCATTCGACCACCAAGCGAAACATCGCATCGAGCGAGCACGTACTCGGATGGAAGCCGGTCTTGTCGATCAGGATGATCTGGACGA
AGAGCATCAGGGGCTCGCGCCAGCCGAACTGTTCGCCAGGCTCAAGGCGCGCATGCCCGACGGCGATGATCTCGTCGTGACCCATGGCGATGCCT
GCTTGCCGAATATCATGGTGGAAAATGGCCGCTTTTCTGGATTCATCGACTGTGGCCGGCTGGGTGTGGCGGACCGCTATCAGGACATAGCGTTG
GCTACCCGTGATATTGCTGAAGAGCTTGGCGGCGAATGGGCTGACCGCTTCCTCGTGCTTTACGGTATCGCCGCTCCCGATTCGCAGCGCATCGCCT
TCTATCGCCTTCTTGACGAGTTCTTCTGA

Primers:

pNOS-BS-F (Primer A/pROK2-Fv): 5'-GGGTTTYTGGAGTTTAATGAGYTAAG-3'
pNOS-BS-R (Primer B/pROK2-Rv): 5'-CACTTCRCCCAATARCARCCARTCCCTTCC-3'

Replicate 1

>PK_328409-501_1-F_Primer-A_A01.ab1/>*ben1*_O_pNOS_no_BST
CNNNNNNNNNNNANATTATTGCGCGTTCNNNGTCGCCTAAGGTCACTATCAGCTAGCAAATATTTCTTGTCAAAAATGCT
CCACTGACGTTCCATAAATTCCCCTCGGTATCCAATTAGAGTCTCATATTCACTCTCAATCCAAATAATCTGCACCGGAT
CTGGATCGTTTCGCATGATTGAACAAGATGGATTGCACGCAGGTTCTCCGGCCGCTTGGGTGGAGAGGCTATTCGGCTAT
GACTGGGCACAACAGACAATCGGCTGCTCTGATGCCGCCGTGTTCCGGCTGTCAGCGCAGGGGCGCCCGGTTCTTTTTGT
CAAGACCGACCTGTCCGGTGCCCTGAATGAACTGCAGGACGAGGCAGCGCGGCTATCGTGGCTGGCCACGACGGGCGTTC
CTTGCGCAGCTGTGCTCGACGTTGTCACTGAAGCGGGAAGGGACTGGCTGCTATTGGGCGAAGTGAACNCNTCAAGCACA
GCTGCGCAGGNNNNNCGTCNTGGCCAGCCNNATAGCCCCGCTNCTTCTCCTGNNNTTCNTTAAGGNNCCGGNANNGGNNG
NCTTGANAAAAAAGAACCGGGGGCCCNNGNNNNAAANCCGGAAACCGGGCGCNNNNAACCCCNNNNNNNNTTGTNNNNGCC
NNANAAANCNNAAANGCCTCCCCCCCNNGGGNNNNNANCNTGNTNNNNTNNNTTNTNTTCGNNNNNNNNNCNNATNNNAN
NNNTNN

>PK_328409-502_1-R_Primer-B_B01.ab1
NNNNNNNNNNNNNNNNNNGTCGAGCACAGCTGCGCAGGAACGCCCGTCGTGGCCAGCCACGATAGCCGCGCTGCCTCGTCC
TGCAGTTCATTCAGGGCACCGGACAGGTCGGTCTTGACAAAAAGAACCGGGCGCCCCTGCGCTGACAGCCGGAACACGGC
GGCATCAGAGCAGCCGATTGTCTGTTGTGCCCAGTCATAGCCGAATAGCCTCTCCACCCAAGCGGCCGGAGAACCTGCGT
GCAATCCATCTTGTTCAATCATGCGAAACGATCCAGATCCGGTGCAGATTATTTGGATTGAGAGTGAATATGAGACTCTA
ATTGGATACCGAGGGGAATTTATGGAACGTCAGTGGAGCATTTTTGACAAGAAATATTTGCTAGCTGATAGTGACCTTAG
GCGACTTTTGAACGCGCAATAATGGTTTCTGACGTATGTGCTTAACTCATTAAACTCCAAAAACNNNN

>RC_328409-502_1-R_Primer-B_B01.ab1/>*ben1*_O_pNOS_no-BST
NNNNGTTTTTGGAGTTTAATGAGTTAAGCACATACGTCAGAAACCATTATTGCGCGTTCAAAAGTCGCCTAAGGTCACTATCAGCTA
GCAAATATTTCTTGTCAAAAATGCTCCACTGACGTTCCATAAATTCCCCTCGGTATCCAATTAGAGTCTCATATTCACTCTCAATCCAAA

TAATCTGCACCGGATCTGGATCGTTTCGCATGATTGAACAAGATGGATTGCACGCAGGTTCTCCGGCCGCTTGGGTGGAGAGGCTAT
TCGGCTATGACTGGGCACAACAGACAATCGGCTGCTCTGATGCCGCCGTGTTCCGGCTGTCAGCGCAGGGGCGCCCGGTTCTTTTTG
TCAAGACCGACCTGTCCGGTGCCCTGAATGAACTGCAGGACGAGGCAGCGCGGCTATCGTGGCTGGCCACGACGGGCGTTCCTGCG
CAGCTGTGCTCGACNNNNNNNNNNNNNNNNN

>PK_328409-503_2-F_Primer-A_C01.ab1/>*ben1*_O_pNOS_BST
CNNNNNNNNANANNTTATTGCNTGTTCNNNTTGCCTAAGNTCACTATCANCTAGCAAATATTTCTTGTCAAAAATGCTCC
ACTGATGTTCCATAAATTCCCCTTGGTATCCAATTAGAGTCTCATATTCACTCTCAATCCAAATAATCTGCACTGGATCT
GGATTGTTTTGCATGATTGAACAAGATGGATTGCATGCAGGTTCTTTGGTTGTTTGGGTGGAGAGGTTATTTGGTTATGA
NTGGGCACAACANATAATTGGCTGCTCTGATGTCGTTGTGTTTNGGTTGTCANNGCANGGNTGCTTGGTTCTTTTTGTCA
AGATTGACCTGTCTGGNGCNCTGAATGAACTGCAGGATGAGGCANNGTGGCTATTGTGGCTGGNCATGATGGGTGTTCCT
TGCGCANCTGTGCTNGATGTTGTCACTGAANTGNGAAGGGACTGGCTGCTATTGGGCGAAGTGA

>PK_328409-504_2-R_Primer-B_D01.ab1
CNNNNNNNNNNNNNNNTCNANCNCNNCTACNCAAAANACACCCATCATANCCAACCACAATAACCACACTACCTCATCCT
ACAATTCATTCAAAACACCAAACAAATCAATCTTAACAAAAAAAACCAAACACCCCTACACTAACAACCAAAACACAACA
ACATCAAAACAACCAATTATCTATTATGCCCAATCATAACCAAATAACCTCTCCACCCAAACAACCCAAAAAANTGCGNT
GCAANCCATCTTTGNNCNATCATGCAAAAAAACCCAAANCCNGGGGAAATTNTTTGGATNGNNAGNGGNANATGANNNNC
TAATTGGATACCAAGGGGAATTTNTGGNANNTCANGGGNNNNTTTTTGANNAGAAATATTTGNTAGCNGNNNNGGGNCTT
ANGGNANTTTTGNANACNCAAAANNNNNTTCNGANNNATGGGGNNNACTCNNTAAACNCCAAAAAACCCAN
>RC_328409-504_2-R_Primer-B_D01.ab1/>*ben1*_O_pNOS-BST
NTGGGTTTTTTGGNGTTTANNGAGTNNNCCCCATNNNTCNGAANNNNNTTTTGNGTNTNCAAAANTNCCNTAAGNCCCNNNNNCN
GCTANCAAATATTTCTNNTCAAAAANNNNCCCNTGANNTNCCANAAATTCCCCTTGGTATCCAATTAGNNNNTCATNTNCCNCTNN
CNATCCAAANAATTTCCCCNGGNTTTGGGTTTTTTTGCATGATNGNNCAAAGATGGNTTGCANCGCANTTTTTTGGGTTGTTTGGGT
GGAGAGGTTATTTGGTTATGATTGGGCATAATAGATAATTGGTTGTTTTGATGTTGTTGTGTTTTGGTTGTTAGTGTAGGGGTGTTTG
GTTTTTTTTGTTAAGATTGATTTGTTTGGTGTTTTGAATGAATTGTAGGATGAGGTAGTGTGGTTATTGTGGTTGGNTATGATGGGTG
TNTTTTGNGTAGNNGNGNTNGANNNNNNNNNNNNNNNG

>PK_328409-505_3-F_Primer-A_E01.ab1/>*ben1*_R_pNOS_BST
NNNNNNNNNNNATTNTTATTNCGCGTTCAAAGTCGTCTAAGGTTACTATCAGTTAGNAAATATTTTTTGTCAAAAATGTTT
TACTGACGTTCCATAAATTTTTCTCGGTATTCAATTAGAGTCTCATATTTATTCTCAATTCAAATAATCTGTACCGGATC
TGGATCGTTTCGTATGATTGAACAAGATGGATTGTACGCANGTTTTCCGGTCGTTTGGGTGGAGAGGCTATTCGGCTATG
ACTGGGTACAACAGACAATCGGCTGTTCTGATGCCGCCGTGTTCCGGCTGTCAGTGCANGGGTGTCCGGTTTTTTTTGTC
AAGACCNATCTGTCCGGTGTTCTGAATGAACTGCANGACGAGGTANCGCGGCTATCGTGGCTGGTTACGANGGGNGTTTT
TTGCNTANCTGTGTTCGATGTTGTTACTGAAGCGGGAANGGACTGGCTGCTATTGGGCGAAGTGAN

>PK_328409-506_3-R_Primer-B_F01.ab1
NNNNNNNNNNNNNNNNCGANNCAGCTANCGCAANAACAGCCNGTCNGNACACCACGATAGCGCGCTANCNCGTCCTNGNAG
TTCATTCAGACACCGGACAGATCGGTCTTGACAANANNACCGGACACCCCTGCACTGACAGCCGNAACACGGCGGCATCA
GAACAGCCGATTGTCTGTTGTACCCAGTCATAGCNNNATANCCTCTCCACCCAANNNNNNNGGAAAACCTGCGTACAATCC
ATCTTGTTCAATCATACGAAACGATCCAGATCCGGTACAGATTATTTGAATTGAGANTGAATATGAGACTCTAATTGAAT
ACCGAGAAAAATTTATGNAACGTCANTNNACCATTTTTGACAAAAAATATTTGCTANCTGATAGTAACCTTAGACGACTT
TTGAACGCGCAATAATANTTTCTGACGTATGTACTTNNTCATTAAACTCCAAAANNNNNNNN

>RC_328409-506_3-R_Primer-B_F01.ab1/>*ben1*_R_pNOS-BST

NNNNNNNNNTTTTGGAGTTTAATGANNAAGTACATACGTCAGAAANTATTATTGCGCGTTCAAAAGTCGTCTAAGGTTACTATCAGN
TAGCAAATATTTTTTGTCAAAAATGGTNNANTGACGTTNCATAAATTTTTCTCGGTATTCAATTAGAGTCTCATATTCANTCTCAATTC
AAATAATCTGTACCGGATCTGGATCGTTTCGTATGATTGAACAAGATGGATTGTACGCAGGTTTTCCNNNNNNTTGGGTGGAGAGG
NTATNNNGCTATGACTGGGTACAACAGACAATCGGCTGTTCTGATGCCGCCGTGTTNCGGCTGTCAGTGCAGGGGTGTCCGGTNNT
NTTGTCAAGACCGATCTGTCCGGTGTCTGAATGAACTNCNAGGACGNGNTAGCGCGCTATCGTGGTGTNCNGACNGGCTGTTNTTG
CGNTAGCTGNNTCGNNNNNNNNNNNNNNN

>PK_328409-507_4-F_Primer-A_G01.ab1/>*Triple*_pNOS-BST
NNNNNNNNNNNNANNNTTATTGCGCGTTCAAAGTCGTCTAAGGTTACTATCAGCTAGCAAATATTTTTTGTCAAAAATGCT
CCACTGACGTTCCATAAATTTTTCTCGGTATTCAATTAGAGTCTCATATTCATTCTCAATTCAAATAATCTGTACCGGAT
CTGGATCGTTTCGTATGATTGAACAAGATGGATTGTACGCAGGTTCTCCGGCCGCTTGGGTGGAGAGGCTATTCGGCTAT
GACTGGGTACAACAGACAATCGGCTGTTCTGATGCCGCCGTGTTCCGGCTGTCAGCGCAGGGGCGTCCGGTTTTTTTTGT
CAAGACCGATCTGTCCGGTGTTCTGAATGAACTGCAGGACGAGGCAGCGCGGCTATCGTGGCTGGTTACGACGGGCGTTT
CTTGCGCAGCTGTGTTCGACGTTGTTACTGAAGCGGGAAGGGACTGGCTGCTATTGGGCGAAGTGA

>PK_328409-508_4-R_Primer-B_H01.ab1
NNNNNNNNNNNNNNNNNNNNNNTCNANCACAGCTGCGCANAACGCCCGTCGTANCCAGNCCACGATAGCNCGCGCTGCCTCG
TCCTGCAGTTCATTCAGAACACCGGACAGATCGGTCTTGACAAAANNNNNCGGACGCCCCTGCGCTGACAGCCGGAACAC
GGCGGCATCAGAACAGCCGATTGTCTGTTGTACCCAGTCATAGCCGAATAGCCTCTCCACCCAAGCGNNNGGANAACCTG
CGTNNNATCCATCTTGTTCAATCATNCGAAACGATCCAGATCCGGTACAGATTATTTGAATTGAGANTGAATATGAGACT
CTAATTGAATACCGAGANAAATTTATGGAACGTCAGTGNAGCATTTTTGACAAAAAATATTTGCTAGCTGATAGTANCCT
TAGACGACTTTTGAACGCGCAATANTNNTTTCTGACGTATGTNCTTAACTCATTAAACTCCANAAACCCN

>RC_328409-508_4-R_Primer-B_H01.ab1/>*Triple*_pNOS-BST
NGGGTTTNTGGAGTTTAATGAGTTAAGNACATACGTCAGAAANNANTATTGCGCGTTCAAAAGTCGTCTAAGGNTACTATCAGCTA
GCAAATATTTTTTGTCAAAAATGCTNCACTGACGTTCCATAAATTTNTCTCGGTATTCAATTAGAGTCTCATATTCANTCTCAATTCAAA
TAATCTGTACCGGATCTGGATCGTTTCGNATGATTGAACAAGATGGATNNNACGCAGGTTNTCCNNNCGCTTGGGTGGAGAGGCTA
TTCGGCTATGACTGGGTACAACAGACAATCGGCTGTTCTGATGCCGCCGTGTTCCGGCTGTCAGCGCAGGGGCGTCCGNNNNNTTTT
GTCAAGACCGATCTGTCCGGTGTTCTGAATGAACTGCAGGACGAGGCAGCGCGNGCTATCGTGGNCTGGNTACGACGGGCGTTNTG
CGCAGCTGTGNTNGANNNNNNNNNNNNNNNNNNN

Sequence alignment:

```
ben1_O_pNOS_no-BST  AAATATTTCTTGTCAAAAATGCTCCACTGACGTTCCATAAATTCCCCTCGGTATCCAATT
ben1_R_pNOS_BST     AAATATTTTTTGTCAAAAATGTTTTACTGACGTTCCATAAATTTTTCTCGGTATTCAATT
ben1_O_pNOS_BST     AAATATTTCTTGTCAAAAATGCTCCACTGATGTTCCATAAATTCCCCTTGGTATCCAATT
                    ******** ************ *  ***** ************   ** ***** *****

ben1_O_pNOS_no-BST  AGAGTCTCATATTCACTCTCAATCCAAATAATCTGCACCGGATCTGGATCGTTTCGCATG
ben1_R_pNOS_BST     AGAGTCTCATATTTATTCTCAATTCAAATAATCTGTACCGGATCTGGATCGTTTCGTATG
ben1_O_pNOS_BST     AGAGTCTCATATTCACTCTCAATCCAAATAATCTGCACTGGATCTGGATTGTTTTGCATG
                    ************* * ******* *********** ** ********** **** * ***

ben1_O_pNOS_no-BST  ATTGAACAAGATGGATTGCACGCAGGTTCTCCGGCCGCTTGGGTGGAGAGGCTATTCGGC
ben1_R_pNOS_BST     ATTGAACAAGATGGATTGTACGCANGTTTTCCGGTCGTTTGGGTGGAGAGGCTATTCGGC
ben1_O_pNOS_BST     ATTGAACAAGATGGATTGCATGCAGGTTCTTTGGTTGTTTGGGTGGAGAGGTTATTTGGT
                    ****************** * *** *** *  **  * ************* **** **
```

```
ben1_O_pNOS_no-BST  TATGACTGGGCACAACAGACAATCGGCTGCTCTGATGCCGCCGTGTTC
ben1_R_pNOS_BST     TATGACTGGGTACAACAGACAATCGGCTGTTCTGATGCCGCCGTGTTC
ben1_O_pNOS_BST     TATGANTGGGCACAACANATAATTGGCTGCTCTGATGTCGTTGTGTTT
                    ***** **** ****** * *** ***** ******* **   *****
```

Replicate 2

>PK_329142-501_2_pROK2-Fv_A04.ab1/>*ben1*_O_pNOS-BST
CCNNNNNNANANNTTATTGCGTGTTCNNNTTGCCTAAGGTCACTATCAGCTAGCAAATATTTCTTGTCAAAAATGCTCCA
CTGATGTTCCATAAATTCCCCTTGGTATCCAATTAGAGTCTCATATTCACTCTCAATCCAAATAATCTGCACTGGATCTG
GATTGTTTTGCATGATTGAACAAGATGGATTGCATGCAGGTTCTTTGGTTGTTTGGGTGGAGAGGTTATTTGGTTATGAT
TGGGTATAATAGATAATTGGTTGCTCTGATGTTGTTGTGTTTTGGTTGTCAGTGCAGGGGTGTTTGGTTCTTTTTGTCAA
GATTGATCTGTCTGGTGCTCTGAATGAACTGCAGGATGAGGCAGTGTGGCTATTGTGGCTGGTCATGATGGGTGTTCCTT
GCGCAGCTGTGCTTGATGTTGTCACTGAAGCGGGAAGGGACTGGCTGCTATTGGGCGAANNTGANCTCCCNCNAGACTGN
CCNANNNNNCNNCCTCCCNCTTTTNCTTGTGANCNNNNANCCANCNTNNCNNNNNNNNNCTNNNCTTCACTCNCNCNNNCT
CNNNCTTTGGTCTTCCCTCNCCCCNCTGCTCTCTCCCTCCCNN

>PK_329142-502_3_pROK2-Fv_B04.ab1/>*ben1*_R_pNOS-BST
NNNNNNNNNNNNNNTTATTGCGCGTTCAAAGTCGTCTAAGGTTACTATCAGTTANCAAATATTTTTTGTCAAAAATGTTNT
ACTGACGTTCCATAAATTTTTCTCGGTATTCAATTAGAGTCTCATATTTATTCTCAATTCAAATAATCTGTACCGGATCT
GGATCGTTTCGTATGATTGAACAAGATGGATTGTACGCAGGTTTTCCGGTCGTTTGGGTGGAGAGGCTATTCGGCTATGA
CTGGGTACAACAGACAATCGGCTGTTCTGATGCCGCCGTGTTCCGGCTGTCAGTGCAGGGGTGTTCGGTTTTTTTTGTCA
NNANCGATCTGTCCGGTGTTCTGAATGAACTGCAGGACGAGGCANCGCGGCTATCGTGGCTGGTTACGATGGGTGTTTTT
TGCGTANCTGTGTTCGATGTTGTTACTGAANCGGGAAGGGACTGGCTGCTATTGGGGCGAANTGANACNNCCAGNGNCCA
CACNCCTTNNTGCCNNCNNCCNNNTCTNNCNNATGACACCCAACANCNNCNNNCGNNTANNNATCNCCNCNNCTNNNCTC
CCGCNNACTCNTGCATAGNNGTCNNNNNNTCCTGACTGCN

>PK_329142-515_2_pROK2-Rv_A06.ab1/>*ben1*_O_pNOS-BST
NNNNNNNNNANNNNNNNNNNNNNCNNCTACNCAAAAGACACCCATCATAACCNACCACAATAACCACACTACCTCATCCTA
CAATTCATTCAAAACACCAAACAAATCAATCTTAACAAAAAAAACCAAACACCCCTACGCTAACAACCAAAACACAACAA
CATCAAAACAACCAATTATCTATTATACCCAATCGTAACCAAATAACCTCTCCACCCAAACAACCAANANAANNNNNGGN
NNNNCNNNTNNNANATAGNAAAAAGNNTNNAANCNGGGGNNANTATTTGGANNGAGAGNGGNATGAGANNTNNNTGGNNN
CCAGGGGAAATTTTGGGACNTTCAGGGGAGCTTTTTGGACAGAAATATTTGGTAACTGGNNANGGNCCTAAGGCAATTTT
GGACACNNAATAAAGGTTTCNGGNNNANGGGGCTNACCCCTTAAACCNANAAACCCAANNAGGNTGCGGGAGNNGNNGNN
GCACCAGNNGCTCGNTGAGAAAANGCNNNNGAGACTGGTACGATGTCGNAGNATCCGNNCCTGATCNTGCTGNGGGCTGN
NNTGCACTGTGTGNAGCNNNACGGCGACTAAATACTTGGTTTGNNATTGGANGTGCCGACGTCATGCTGGGGTGAANGTG
CCTGTG

>PK_329142-516_3_pROK2-Rv_B06.ab1/>*ben1*_R_pNOS-BST
NNNNNNNNNNNNANNNNCNNNNNCNGCNNNNNNNNNNNNNCCCNNNCNNNAANNNNCNNNATAAGCCNGCGNTAGCNCGGN
CCTGNANNNNNTTCNNANCACCGGACNNATCGGCCTNGANNNNNNNNACCGGANNCCCCTGCNCTGACAACCGGAACACGG
NGGCATCANANCAGNNGATNGTCTGTTGTACCNANNGNTAGCCNATTAACCTTCTCCCCCCCAANCGNCCCGGAAAACCT
GCGTACAATCCATCTTGTTCAATCATACGAAACGATCCAGATCCGGTACAGATTATTTGAATTGAGAGTGAATATNANAC
TCTAATTGAATACCGANAAAANNTNATGGAACGTCNGNGNAANATTTTTGNNAAAAAAATATTTGCTANCTGATAGTAAC
CTTAGACGACTTTTGAACGCGCAATANTAATTTCGGACGTATGTACTTAACTCATTAAACTCNNAAAAANNAAN

File S4

***NPTII* gene sequencing V2**

GTTTACCCGCCAATATATCCTGTCAAACACTGATAGTTTAAACTGAAGGCGGGAAACGACAATCTGATCATGAGCGGAGAATTAAGGGAGTCACGT
TATGACCCCCGCCGATGACGCGGGACAAGCCGTTTTACGTTTGGAACTGACAGAACCGCAACGTTGAAGGAGCCACTCAG**CCG/CGG**GTTTCTGGA
GTTTAATGAGCTAAGCACATACGTCAGAAACCATTATTGCGCGTTCAAAAGTCGCCTAAGGTCACTATCAGCTAGCAAATATTTCTTGTCAAAAATG
CTCCACTGACGTTCCATAAATTCCCCTCGGTATCCAATTAGAGTCTCATATTCACTCTCAATCCAAATAATCTGCACCGGATCTGGATCGTTTCGCATG
ATTGAACAAGATGGATTGCACGCAGGTTCTCCGGCCGCTTGGGTGGAGAGGCTATTCGGCTATGACTGGGCACAACAGACAATCGGCTGCTCTGA
TGCCGCCGTGTTCCGGCTGTCAGCGCAGGGGCGCCCGGTTCTTTTTGTCAAGACCGACCTGTCCGGTGCCCTGAATGAACTGCAGGACGAGGCAG
CGCGGCTATCGTGGCTGGCCACGACGGGCGTTCCTTGCGCAGCTGTGCTCGACGTTGTCACTGAAGCGGGAAGGGACTGGCTGCTATTGGGCGAA
GTGCCGGGGCAGGATCTCCTGTCATCTCACCTTGCTCCTGCCGAGAAAGTATCCATCATGGCTGATGCAATGCGGCGGCTGCATACGCTTGATCCG
GCTACCTGCCCATTCGACCACCAAGCGAAACATCGCATCGAGCGAGCACGTACTCGGATGGAAGCCGGTCTTGTCGATCAGGATGATCTGGACGA
AGAGCATCAGGGGCTCGCGCCAGCCGAACTGTTCGCCAGGCTCAAGGCGCGCATGCCCGACGGCGATGATCTCGTCGTGACCCATGGCGATGCCT
GCTTGCCGAATATCATGGTGGAAAATGGCCGCTTTTCTGGATTCATCGACTGTGGCCGGCTGGGTGTGGCGGACCGCTATCAGGACATAGCGTTG
GCTACCCGTGATATTGCTGAAGAGCTTGGCGGCGAATGGGCTGACCGCTTCCTCGTGCTTTACGGTATCGCCGCTCCCGATTCGCAGCGCATCGCCT
TCTATCGCCTTCTTGACGAGTTCTTCTGAGCGGGACTCTGGGGTTCGAAATGACCGACCAAGCGACGCCCAACCTGCCATCACGAGATTTCGATTCC
ACCGCCGCCTTCTATGAAAGGTTGGGCTTCGGAATCGTTTTCCGGGACGCCGGCTGGATGATCCTCCAGCGCGGGGATCTCATGCTGGAGTTCTTC
GCCCACGGGATCTCTGCGGAACA

>ben1_O_NPTII-F1
NNNNNNNACNTNNNNGAGCCACTCAGCCGCGGGTTTCTGGAGTTTAATGAGCTAAGCACATACGTCAGAAACCATTATTG
CGCGTTCAAAAGTCGCCTAAGGTCACTATCAGCTAGCAAATATTTCTTGTCAAAAATGCTCCACTGACGTTCCATAAATT
CCCCTCGGTATCCAATTAGAGTCTCATATTCACTCTCAATCCAAATAATCTGCACCGGATCTGGATCGTTTCGCATGATT
GAACAAGATGGATTGCACGCAGGTTCTCCGGCCGCTTGGGTGGAGAGGCTATTCGGCTATGACTGGGCACAACAGACAAT
CGGCTGCTCTGATGCCGCCGTGTTCCGGCTGTCAGCGCAGGGGCGCCCGGTTCTTTTTGTCAAGACCGACCTGTCCGGTG
CCCTGAATGAACTGCAGGACGAGGCAGCGCGGCTATCGTGGCTGGCCACGACGGGCGTTCCTTGCGCAGCTGTGCTCGAC
GTTGTCACTGAAGCGGGAAGGGACTGGCTGCTATTGGGCGAAGTGCCGGGGCAGGATCTCCTGTCATCTCACCTTGCTCC
TGCCGAGAAAGTATCCATCATGGCTGATGCAATGCGGCGGCTGCATACGCTTGATCCGGCTACCTGCCCATTCGACCACC
AAGCGAAACATCGCATCGAGCGAGCACGTACTCGGATGGAAGCCGGTCTTGTCGATCAGGATGATCTGGACGAAGAGCAT
CAGGGGCTCGCGCCAGCCGAACTGTTCGCCAGGCTCAAGGCGCGCATGCCCGACGGCGATGATCTCGTCGTGACCCATGG
CGATGCCTGCTTGCCGAATATCATGGTGGAAAATGGCCGCTTTTCTGGATTCATCGACTGTGGCCGGCTGGGTGTGGCGG
ACCGCTATCNNNCATAGCGTTGGCTACCCGTGATATTGCTGAAGAGCTTGGCGGCGAATGGGCTGACCGCTTCCTCGTGC
TTTACGGTATCGCCGCTCCCGATTCGCAGCGCATCNCCTTCTATCGCCTNCTTGACNAGTTCTTCTGANCGGNNNNNNNN
NNGGGGNNNNNNNNNNNNNNNNNAAAAAGNAN

>ben1_R_NPTII-F1
NNNNNNNACNTNNANGANCCNCTCAGCCGCGGGTTTCTGGAGTTTAATGAGCTAAGCACATACGTCAGAAACCATTATTG
CGCGTTCAAAAGTCGCCTAAGGTCACTATCAGCTAGCAAATATTTCTTGTCAAAAATGCTCCACTGACGTTCCATAAATT
CCCCTCGGTATCCAATTAGAGTCTCATATTCACTCTCAATCCAAATAATCTGCACCGGATCTGGATCGTTTCGCATGATT
GAACAAGATGGATTGCACGCAGGTTCTCCGGCCGCTTGGGTGGAGAGGCTATTCGGCTATGACTGGGCACAACAGACAAT
CGGCTGCTCTGATGCCGCCGTGTTCCGGCTGTCAGCGCAGGGGCGCCCGGTTCTTTTTGTCAAGACCGACCTGTCCGGTG
CCCTGAATGAACTGCAGGACGAGGCAGCGCGGCTATCGTGGCTGGCCACGACGGGCGTTCCTTGCGCAGCTGTGCTCGAC
GTTGTCACTGAAGCGGGAAGGGACTGGCTGCTATTGGGCGAAGTGCCGGGGCAGGATCTCCTGTCATCTCACCTTGCTCC
TGCCGAGAAAGTATCCATCATGGCTGATGCAATGCGGCGGCTGCATACGCTTGATCCGGCTACCTGCCCATTCGACCACC
AAGCGAAACATCGCATCGAGCGAGCACGTACTCGGATGGAAGCCGGTCTTGTCGATCAGGATGATCTGGACGAAGAGCAT
CAGGGGCTCGCGCCAGCCGAACTGTTCGCCAGGCTCAAGGCGCGCATGCCCGACGGCGATGATCTCGTCGTGACCCATGG
CGATGCCTGCTTGCCGAATATCATGGTGGAAAATGGCCGCTTTTCTGGATTCATCGACTGTGGCCGGCTGGGTGTGGCGG
ACCGCTATCNGACATAGCGTTGGCTACCCGTGATATTGCTGAAGAGCTTGGCGGCGAATGGGCTGACCGCTTCCTCGTGC
TTTACGGTATCGCCGCTCCCGATTCNCAGCGCATCGCCTTCTATCGCCTTCTTGACGANTTCTTCTGANCNGGNNNNNNN
NNNNNNGGNNNGNNNANNNNNAA

```
>ben1_O_NPTII-F2
NNNNNNNNGNGGCGGNNNGCNNNNNNCNNNNNTCCGGCTNACCTNNCCCATTCGACCACCAAGCGAAACATCGCATCGAG
CGAGCACGTACTCGGATGGAAGCCGGTCTTGTCGATCAGGATGATCTGGACGAAGAGCATCAGGGGCTCGCGCCAGCCGA
ACTGTTCGCCAGGCTCAAGGCGCGCATGCCCGACGGCGATGATCTCGTCGTGACCCATGGCGATGCCTGCTTGCCGAATA
TCATGGTGGAAAATGGCCGCTTTTCTGGATTCATCGACTGTGGCCGGCTGGGTGTGGCGGACCGCTATCAGGACATAGCG
TTGGCTACCCGTGATATTGCTGAAGAGCTTGGCGGCGAATGGGCTGACCGCTTCCTCGTGCTTTACGGTATCGCCGCTCC
CGATTCGCAGCGCATCGCCTTCTATCGCCTTCTTGACGAGTTCTTCTGAGCGGGACTCTGGGGNNGAAATGAAAGAACCC
CCTCCNNACCTTCCCCCGGATGGGGGNCGAGGGAGCTGATCGCCANGNGGNNNCCANCNNNNANNACAANCCNCCGGCTT
GTGCTNNTTTTNNNCNNNNTTGTTGGTTATTTCNNANNNNTCNCNNNCCNNANNANTCATCNTNCTCACNNCACCNNNNN
NNCNNCNNNNTGTTGTTTTTNTTCTGATTGTTGATCANNNNNCTAANNAGCNNGGCTNNTTTTCTTNNNNCAGAGNNNAA
NNAAACACGCCNACNNCATNCNCNCCCNTTANTCNNTCTTCAGANACGANNTCANNNNNACAAACTNNNNNGCGCNANAN
ANANNATNTATCNCNNNACTCNNNNCTNGAAATANNNTACGANNNNNANANNAAAAANCANCCCCTCTTGCGTGGNTNTN
NACGAGAGTNNNTAGNNNNCNNNNGCGNAAAATNNNNNANACANNNANAGNNNNTGNNTTNATANNCGNCTANTNNTNGG
ANNNNTNNGTNATCNANNTNTNNANNNTNNNNTGTTCNCTNTGNGCCTGAATTTCNCTNTNNTCCCCCCNNNNNNTNNNN
NNCNNGGCTATCNGGCNNNTNTNTTTNTACNNTNTTGGAGGGAAGNNNNNNNNNNNNNNN
```

```
>ben1_R_NPTII-F2
NNNNNNNNNGCGGNNNNNNNNNNNNNNNCNNNNNNCCGNNTNACNTNNCCCATTTNCGACCACCAAGCGAAACATCGCATC
GAGCGAGCACGTACTCGGATGGAAGCCGGTCTNNGTCGATCAGGATGATCTGGACGAAGAGCATCAGGGGCTCGCGCCAG
CCGAACTGTTCGCCAGGCTCAAGGCGCGCATGCCCGACGGCGATGATCTCGTCGTGACCCATGGCGATGCCTGCTTGCCG
AATATCATGGTGGAAAATGGCCGCTTTTCTGGATTCATCGACTGTGGCCGGCTGGGTGTGGCGGACCGCTATCAGGACAT
AGCGTTGGCTACCCGTGATATTGCTGAAGAGCTTGGCGGCGAATGGGCTGACCGCTTCCTCGTGCTTTACGGTATCGCCG
CTCCCGATTCGCAGCGCATCGCCTTCTATCGCCTTCTTGACGAGTTCTTCTGAGCGGGACTCTGGGGNNCNAAATGAANN
CATCATCNNTCNNNNNGACNGGTGGNGGGACNGNGTGCAGTAGCNNAAGNCTAACAACTAACATGACCATCTCGNNGGAG
CTTTTTTCCACTTGAGAGACATTNCTTTTGGCTGTACNNCNTCCTNNNCCCTCCCATCATCTTTNNGCANCNCCNGCCAA
NTNTCNTNCNTCCTTTTGNTTCTNNTTNNTNNTCNNCANCNNNTGGGCTGGCCNGGNGTCTCTTGTNTACTANGNNGNNN
TGGACGGACCCGNNNNNNNGGGTTCCGNTTCNNTANAAAANCGTACCTNNNNGNNGCNNAGGGNNNNNGCCCCCAANNGG
NAAAAANTCTTCGTNCNTNNNCCCCGNANNAAGGNNNNATTGNNCNNAATANANANGACNGGCAAAANGGNGNNCNCNTN
NNNNGGNANTCNNNNNNNGGAGGGNCGGNGNNGGGTCNNGGNNNNAACCNTNTTCNCAANNNNNNAANNNNNTGNNNCNN
CCCNNNGNTNANTGNNTNGAANGANTTTTGCTNTCNNNCTGGGNNNNNATCTNNNTTNCNCTNNNGGTTTNTANNGNNNT
NNNNCCNCTCTCNCANNNNANTNNNNNAAGCNNNTANNNNGGGTTTTNNTTNNNNNTTTTTTNNNNNCGNNANNTNTNNN
TNNCNTNNNNNNNNNNNNNNNNNTNNNNNGGGGNNNTTTTNNGGNNAAAATGNNNANNNNNNNNN
```

```
ben1_O_NPTII-F1   NNNNNNNACNTNNNNGAGCCACTCAGCCGCGGGTTTCTGGAGTTTAATGAGCTAAGCACA
ben1_R_NPTII-F1   NNNNNNNACNTNNANGANCCNCTCAGCCGCGGGTTTCTGGAGTTTAATGAGCTAAGCACA
                  ************* *** ** ***************************************

ben1_O_NPTII-F1   TACGTCAGAAACCATTATTGCGCGTTCAAAAGTCGCCTAAGGTCACTATCAGCTAGCAAA
ben1_R_NPTII-F1   TACGTCAGAAACCATTATTGCGCGTTCAAAAGTCGCCTAAGGTCACTATCAGCTAGCAAA
                  ************************************************************

ben1_O_NPTII-F1   TATTTCTTGTCAAAAATGCTCCACTGACGTTCCATAAATTCCCCTCGGTATCCAATTAGA
ben1_R_NPTII-F1   TATTTCTTGTCAAAAATGCTCCACTGACGTTCCATAAATTCCCCTCGGTATCCAATTAGA
                  ************************************************************

ben1_O_NPTII-F1   GTCTCATATTCACTCTCAATCCAAATAATCTGCACCGGATCTGGATCGTTTCGCATGATT
ben1_R_NPTII-F1   GTCTCATATTCACTCTCAATCCAAATAATCTGCACCGGATCTGGATCGTTTCGCATGATT
                  ************************************************************

ben1_O_NPTII-F1   GAACAAGATGGATTGCACGCAGGTTCTCCGGCCGCTTGGGTGGAGAGGCTATTCGGCTAT
ben1_R_NPTII-F1   GAACAAGATGGATTGCACGCAGGTTCTCCGGCCGCTTGGGTGGAGAGGCTATTCGGCTAT
                  ************************************************************
```

```
ben1_O_NPTII-F1   GACTGGGCACAACAGACAATCGGCTGCTCTGATGCCGCCGTGTTCCGGCTGTCAGCGCAG
ben1_R_NPTII-F1   GACTGGGCACAACAGACAATCGGCTGCTCTGATGCCGCCGTGTTCCGGCTGTCAGCGCAG
                  ************************************************************

ben1_O_NPTII-F1   GGGCGCCCGGTTCTTTTTGTCAAGACCGACCTGTCCGGTGCCCTGAATGAACTGCAGGAC
ben1_R_NPTII-F1   GGGCGCCCGGTTCTTTTTGTCAAGACCGACCTGTCCGGTGCCCTGAATGAACTGCAGGAC
                  ************************************************************

ben1_O_NPTII-F1   GAGGCAGCGCGGCTATCGTGGCTGGCCACGACGGGCGTTCCTTGCGCAGCTGTGCTCGAC
ben1_R_NPTII-F1   GAGGCAGCGCGGCTATCGTGGCTGGCCACGACGGGCGTTCCTTGCGCAGCTGTGCTCGAC
                  ************************************************************

ben1_O_NPTII-F1   GTTGTCACTGAAGCGGGAAGGGACTGGCTGCTATTGGGCGAAGTGCCGGGGCAGGATCTC
ben1_R_NPTII-F1   GTTGTCACTGAAGCGGGAAGGGACTGGCTGCTATTGGGCGAAGTGCCGGGGCAGGATCTC
                  ************************************************************

ben1_O_NPTII-F1   CTGTCATCTCACCTTGCTCCTGCCGAGAAAGTATCCATCATGGCTGATGCAATGCGGCGG
ben1_R_NPTII-F1   CTGTCATCTCACCTTGCTCCTGCCGAGAAAGTATCCATCATGGCTGATGCAATGCGGCGG
                  ************************************************************

ben1_O_NPTII-F1   CTGCATACGCTTGATCCGGCTACCTGCCCATTCGACCACCAAGCGAAACATCGCATCGAG
ben1_R_NPTII-F1   CTGCATACGCTTGATCCGGCTACCTGCCCATTCGACCACCAAGCGAAACATCGCATCGAG
                  ************************************************************

ben1_O_NPTII-F1   CGAGCACGTACTCGGATGGAAGCCGGTCTTGTCGATCAGGATGATCTGGACGAAGAGCAT
ben1_R_NPTII-F1   CGAGCACGTACTCGGATGGAAGCCGGTCTTGTCGATCAGGATGATCTGGACGAAGAGCAT
                  ************************************************************

ben1_O_NPTII-F1   CAGGGGCTCGCGCCAGCCGAACTGTTCGCCAGGCTCAAGGCGCGCATGCCCGACGGCGAT
ben1_R_NPTII-F1   CAGGGGCTCGCGCCAGCCGAACTGTTCGCCAGGCTCAAGGCGCGCATGCCCGACGGCGAT
                  ************************************************************

ben1_O_NPTII-F1   GATCTCGTCGTGACCCATGGCGATGCCTGCTTGCCGAATATCATGGTGGAAAATGGCCGC
ben1_R_NPTII-F1   GATCTCGTCGTGACCCATGGCGATGCCTGCTTGCCGAATATCATGGTGGAAAATGGCCGC
                  ************************************************************

ben1_O_NPTII-F1   TTTTCTGGATTCATCGACTGTGGCCGGCTGGGTGTGGCGGACCGCTATCNNNCATAGCGT
ben1_R_NPTII-F1   TTTTCTGGATTCATCGACTGTGGCCGGCTGGGTGTGGCGGACCGCTATCNGACATAGCGT
                  **************************************************  ********

ben1_O_NPTII-F1   TGGCTACCCGTGATATTGCTGAAGAGCTTGGCGGCGAATGGGCTGACCGCTTCCTCGTGC
ben1_R_NPTII-F1   TGGCTACCCGTGATATTGCTGAAGAGCTTGGCGGCGAATGGGCTGACCGCTTCCTCGTGC
                  ************************************************************

ben1_O_NPTII-F1   TTTACGGTATCGCCGCTCCCGATTCGCAGCGCATCNCCTTCTATCGCCTNCTTGACNAGT
ben1_R_NPTII-F1   TTTACGGTATCGCCGCTCCCGATTCNCAGCGCATCGCCTTCTATCGCCTTCTTGACGANT
                  ************************* ********* ************* ****** * *

ben1_O_NPTII-F1   TCTTCTGANCGGNNNNNNNNNNGGGNNNNNNNNNNNNNNNNNAAAAAGNAN
ben1_R_NPTII-F1   TCTTCTGANCNGGNNNNNNNNNNNNNNNGGNNNGNNNANNNNNNAA--------
                  ********** * *********      *** *** ***** *


ben1_O_NPTII-F2   -NNNNNNNNGNGGCGGNNNGCNNNNNNCNNNNNTCCGGCTNACCTNNCCCATT--CGACC
ben1_R_NPTII-F2   NNNNNNNNNGCGGNNNNNNNNNNNNNNCNNNNNNCCGNNTNACNTNNCCCATTTNCGACC
                   ********* **   ***  ************ ***  **** *********  *****
```

```
ben1_O_NPTII-F2   ACCAAGCGAAACATCGCATCGAGCGAGCACGTACTCGGATGGAAGCCGGTCTTG-TCGAT
ben1_R_NPTII-F2   ACCAAGCGAAACATCGCATCGAGCGAGCACGTACTCGGATGGAAGCCGGTCTNNGTCGAT
                  ****************************************************   *****

ben1_O_NPTII-F2   CAGGATGATCTGGACGAAGAGCATCAGGGGCTCGCGCCAGCCGAACTGTTCGCCAGGCTC
ben1_R_NPTII-F2   CAGGATGATCTGGACGAAGAGCATCAGGGGCTCGCGCCAGCCGAACTGTTCGCCAGGCTC
                  ************************************************************

ben1_O_NPTII-F2   AAGGCGCGCATGCCCGACGGCGATGATCTCGTCGTGACCCATGGCGATGCCTGCTTGCCG
ben1_R_NPTII-F2   AAGGCGCGCATGCCCGACGGCGATGATCTCGTCGTGACCCATGGCGATGCCTGCTTGCCG
                  ************************************************************

ben1_O_NPTII-F2   AATATCATGGTGGAAAATGGCCGCTTTTCTGGATTCATCGACTGTGGCCGGCTGGGTGTG
ben1_R_NPTII-F2   AATATCATGGTGGAAAATGGCCGCTTTTCTGGATTCATCGACTGTGGCCGGCTGGGTGTG
                  ************************************************************

ben1_O_NPTII-F2   GCGGACCGCTATCAGGACATAGCGTTGGCTACCCGTGATATTGCTGAAGAGCTTGGCGGC
ben1_R_NPTII-F2   GCGGACCGCTATCAGGACATAGCGTTGGCTACCCGTGATATTGCTGAAGAGCTTGGCGGC
                  ************************************************************

ben1_O_NPTII-F2   GAATGGGCTGACCGCTTCCTCGTGCTTTACGGTATCGCCGCTCCCGATTCGCAGCGCATC
ben1_R_NPTII-F2   GAATGGGCTGACCGCTTCCTCGTGCTTTACGGTATCGCCGCTCCCGATTCGCAGCGCATC
                  ************************************************************

ben1_O_NPTII-F2   GCCTTCTATCGCCTTCTTGACGAGTTCTTCTGAGCGGGACTCTGGGGNN-GAAATGAAAG
ben1_R_NPTII-F2   GCCTTCTATCGCCTTCTTGACGAGTTCTTCTGAGCGGGACTCTGGGGNNCNAAATGAANN
                  *************************************************  *******
```

File S5
BEN1 BL-100 analysis

File S6
Fluence rate analysis

File S7
Next generation unstable *ben1-1* Hypocotyl data

File S8
Real time RT-PCR Expression data

Files S5-S8 are available for download at http://www.g3journal.org/lookup/suppl/doi:10.1534/g3.113.006353/-/DC1.

Table S1 Table of PCR primer sequences used in the study.

Primer Name	Primer Sequences
GSP1	5'-TTTATATCTTCGTCGTTGCCT-3'
GSP2	5'-ACCTTTTGCAACTGGCTTTT-3'
LBb1.3	5'-ATTTTGCCGATTTCGGAAC-3'
PRT1	5'-ATGGTGAGAGAAGAACAAGA-3'
PRT2	5'-TTAAAGAAATCCCCTTGCTTG-3'
PQ1	5'-ATCTCTCGCCATGCTCTTTG-3'
PQ2	5'-CTTCATCTCCACCGACGAAC-3'
UBQ1	5'-GAAATGCATGGAGACGGATT-3'
UBQ2	5'-TTGGTCTCTGCTCCCACTCT-3'
ACTIN2 RT-F	5'-GGTCGTACAACCGGTATTGTGCTGG-3'
ACTIN2 RT-R	5'-CTGTGAACGATTCCTGGACCTGCC-3'
PQ3	5'-TCTAAACAAGGAATTCGAGGTC-3'
PQ4	5'-AACCAGAAATGTAGCACCGT-3'
NPT-Fv1/MT1	5'-ACAAGCCGTTTTACGTTTGG-3'
NPT-Rv	5'-TCATTTCGAACCCCAGAGTC-3'
MT2	5'-ATACTTTCTCGGCAGGAGCA-3'
MT3	5'-TTCGCAAGACCCTTCCTCTA-3'
MT4	5'-GTTTTCCCAGTCACGACGTT-3'
NPT-Fv2	5'-TGCTCCTGCCGAGAAAGTAT-3'
pNOS-BS-F	5'-GGGTTTYTGGAGTTTAATGAGYTAAG-3'
pNOS-BS-R	5'-CACTTCRCCCAATARCARCCARTCCCTTCC-3'
pBEN1-BS-F	5'-GTATAGTAGTAAAAAAAAGAAGAAGAAG-3'
pBEN1-BS-R	5'-CTTRTTCTTCTCTCACCATTTTCTCTCT-3'
BEN1-E2-BS-F	5'-TTAGGAATTAAAAGGAGAAGAG-3'
BEN1-E2-BS-R	5'-AATTAAATACCAAAAAACATAAC-3'
