# Supplementary material for: The ben1-1 Brassinosteroid-Catabolism Mutation Is Unstable Due to Epigenetic Modifications of the Intronic T-DNA Insertion
Source: G3 (Bethesda). 2013 Sep 1;3(9):1587–95. doi: 10.1534/g3.113.006353 (PMC3755919; doi:10.1534/g3.113.006353)
Supplement: Supporting Information [file supp_g3.113.006353_FigureS1.pdf]

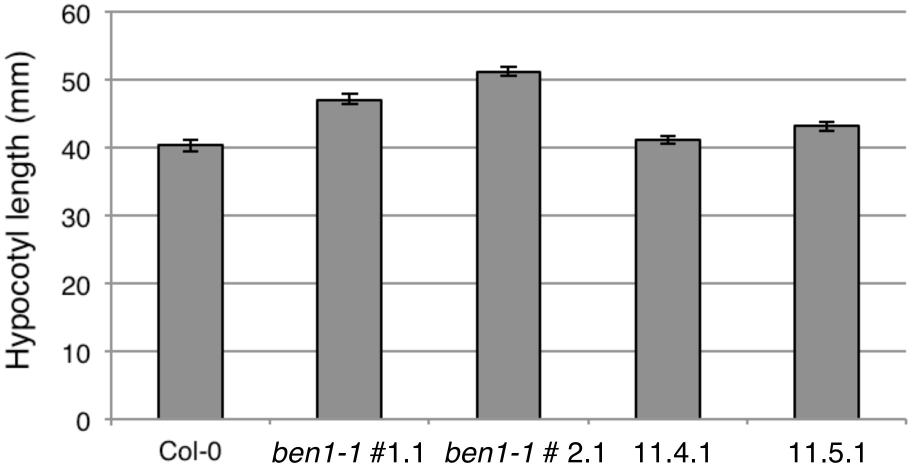

Figure S1 The attenuated hypocotyl-elongation phenotype of the re-isolated *ben1-1* lines is stably inherited to the next generation. The hypocotyl-elongation phenotype of progeny from the re-isolated *ben1-1* lines is significantly different from the original *ben1-1* lines ($p < 0.05$). Seedlings were grown in darkness or 9 µmol m^{-2} sec^{-1} of white-light for five days before being digitized and measured. Hypocotyl lengths were then normalized to seedlings grown in the dark and expressed as a percentage change. Error bars represent standard error (SE). To calculate SE, each seedling value in light was normalized to the average of the genotype in dark. The resulting group of values was used to calculate standard error (SE).
