# Supplementary material for: The ben1-1 Brassinosteroid-Catabolism Mutation Is Unstable Due to Epigenetic Modifications of the Intronic T-DNA Insertion
Source: G3 (Bethesda). 2013 Sep 1;3(9):1587–95. doi: 10.1534/g3.113.006353 (PMC3755919; doi:10.1534/g3.113.006353)
Supplement: Supporting Information [file supp_g3.113.006353_FigureS2.pdf]

```
ben1_O_NPTII-F1   NNNNNNNACNTNNNNGAGCCACTCAGCCGCGGGTTTCTGGAGTTTAATGAGCTAAGCACA
ben1_R_NPTII-F1   NNNNNNNACNTNNANGANCCNCTCAGCCGCGGGTTTCTGGAGTTTAATGAGCTAAGCACA
                  ************* *** ** ***************************************

ben1_O_NPTII-F1   TACGTCAGAAACCATTATTGCGCGTTCAAAAGTCGCCTAAGGTCACTATCAGCTAGCAAA
ben1_R_NPTII-F1   TACGTCAGAAACCATTATTGCGCGTTCAAAAGTCGCCTAAGGTCACTATCAGCTAGCAAA
                  ************************************************************

ben1_O_NPTII-F1   TATTTCTTGTCAAAAATGCTCCACTGACGTTCCATAAATTCCCCTCGGTATCCAATTAGA
ben1_R_NPTII-F1   TATTTCTTGTCAAAAATGCTCCACTGACGTTCCATAAATTCCCCTCGGTATCCAATTAGA
                  ************************************************************

ben1_O_NPTII-F1   GTCTCATATTCACTCTCAATCCAAATAATCTGCACCGGATCTGGATCGTTTCGCATGATT
ben1_R_NPTII-F1   GTCTCATATTCACTCTCAATCCAAATAATCTGCACCGGATCTGGATCGTTTCGCATGATT
                  ************************************************************

ben1_O_NPTII-F1   GAACAAGATGGATTGCACGCAGGTTCTCCGGCCGCTTGGGTGGAGAGGCTATTCGGCTAT
ben1_R_NPTII-F1   GAACAAGATGGATTGCACGCAGGTTCTCCGGCCGCTTGGGTGGAGAGGCTATTCGGCTAT
                  ************************************************************

ben1_O_NPTII-F1   GACTGGGCACAACAGACAATCGGCTGCTCTGATGCCGCCGTGTTCCGGCTGTCAGCGCAG
ben1_R_NPTII-F1   GACTGGGCACAACAGACAATCGGCTGCTCTGATGCCGCCGTGTTCCGGCTGTCAGCGCAG
                  ************************************************************

ben1_O_NPTII-F1   GGGCGCCCGGTTCTTTTTGTCAAGACCGACCTGTCCGGTGCCCTGAATGAACTGCAGGAC
ben1_R_NPTII-F1   GGGCGCCCGGTTCTTTTTGTCAAGACCGACCTGTCCGGTGCCCTGAATGAACTGCAGGAC
                  ************************************************************

ben1_O_NPTII-F1   GAGGCAGCGCGGCTATCGTGGCTGGCCACGACGGGCGTTCCTTGCGCAGCTGTGCTCGAC
ben1_R_NPTII-F1   GAGGCAGCGCGGCTATCGTGGCTGGCCACGACGGGCGTTCCTTGCGCAGCTGTGCTCGAC
                  ************************************************************

ben1_O_NPTII-F1   GTTGTCACTGAAGCGGGAAGGGACTGGCTGCTATTGGGCGAAGTGCCGGGGCAGGATCTC
ben1_R_NPTII-F1   GTTGTCACTGAAGCGGGAAGGGACTGGCTGCTATTGGGCGAAGTGCCGGGGCAGGATCTC
                  ************************************************************

ben1_O_NPTII-F1   CTGTCATCTCACCTTGCTCCTGCCGAGAAAGTATCCATCATGGCTGATGCAATGCGGCGG
ben1_R_NPTII-F1   CTGTCATCTCACCTTGCTCCTGCCGAGAAAGTATCCATCATGGCTGATGCAATGCGGCGG
                  ************************************************************

ben1_O_NPTII-F1   CTGCATACGCTTGATCCGGCTACCTGCCCATTCGACCACCAAGCGAAACATCGCATCGAG
ben1_R_NPTII-F1   CTGCATACGCTTGATCCGGCTACCTGCCCATTCGACCACCAAGCGAAACATCGCATCGAG
                  ************************************************************

ben1_O_NPTII-F1   CGAGCACGTACTCGGATGGAAGCCGGTCTTGTCGATCAGGATGATCTGGACGAAGAGCAT
ben1_R_NPTII-F1   CGAGCACGTACTCGGATGGAAGCCGGTCTTGTCGATCAGGATGATCTGGACGAAGAGCAT
                  ************************************************************
```

```
ben1_O_NPTII-F2   ACCAAGCGAAACATCGCATCGAGCGAGCACGTACTCGGATGGAAGCCGGTCTTG-TCGAT
ben1_R_NPTII-F2   ACCAAGCGAAACATCGCATCGAGCGAGCACGTACTCGGATGGAAGCCGGTCTNNGTCGAT
                  ****************************************************   *****

ben1_O_NPTII-F2   CAGGATGATCTGGACGAAGAGCATCAGGGGCTCGCGCCAGCCGAACTGTTCGCCAGGCTC
ben1_R_NPTII-F2   CAGGATGATCTGGACGAAGAGCATCAGGGGCTCGCGCCAGCCGAACTGTTCGCCAGGCTC
                  ************************************************************

ben1_O_NPTII-F2   AAGGCGCGCATGCCCGACGGCGATGATCTCGTCGTGACCCATGGCGATGCCTGCTTGCCG
ben1_R_NPTII-F2   AAGGCGCGCATGCCCGACGGCGATGATCTCGTCGTGACCCATGGCGATGCCTGCTTGCCG
                  ************************************************************

ben1_O_NPTII-F2   AATATCATGGTGGAAAATGGCCGCTTTTCTGGATTCATCGACTGTGGCCGGCTGGGTGTG
ben1_R_NPTII-F2   AATATCATGGTGGAAAATGGCCGCTTTTCTGGATTCATCGACTGTGGCCGGCTGGGTGTG
                  ************************************************************

ben1_O_NPTII-F2   GCGGACCGCTATCAGGACATAGCGTTGGCTACCCGTGATATTGCTGAAGAGCTTGGCGGC
ben1_R_NPTII-F2   GCGGACCGCTATCAGGACATAGCGTTGGCTACCCGTGATATTGCTGAAGAGCTTGGCGGC
                  ************************************************************

ben1_O_NPTII-F2   GAATGGGCTGACCGCTTCCTCGTGCTTTACGGTATCGCCGCTCCCGATTCGCAGCGCATC
ben1_R_NPTII-F2   GAATGGGCTGACCGCTTCCTCGTGCTTTACGGTATCGCCGCTCCCGATTCGCAGCGCATC
                  ************************************************************

ben1_O_NPTII-F2   GCCTTCTATCGCCTTCTTGACGAGTTCTTCTGAGCGGGACTCTGGGGNN-GAAATGAAAG
ben1_R_NPTII-F2   GCCTTCTATCGCCTTCTTGACGAGTTCTTCTGAGCGGGACTCTGGGGNNCNAAATGAANN
                  *************************************************  *******
```

Figure S2 ***NPTII* gene sequence did not change in the pre-triple and post-triple *ben1-1* mutants.** The *NPTII* gene was amplified using NPT-Fv1, NPT-Fv2 and NPT-Rv from the pre-triple and post-triple *ben1-1* lines. Primers NPT-Fv1 and NPT-Fv2 were used for sequencing *NPTII* gene (Table S1). The color coded yellow nucleotides show the complete *NPTII* coding region in the sequence.
