# Supplementary material for: The ben1-1 Brassinosteroid-Catabolism Mutation Is Unstable Due to Epigenetic Modifications of the Intronic T-DNA Insertion
Source: G3 (Bethesda). 2013 Sep 1;3(9):1587–95. doi: 10.1534/g3.113.006353 (PMC3755919; doi:10.1534/g3.113.006353)
Supplement: Supporting Information [file supp_g3.113.006353_FigureS3.pdf]

(A)

```
ben1_O_pBEN1_BST  GGAATTTAATAATAATGTTTGTAATAAATGGATAAAGAATGTGAATTATTTATATAAATG
ben1_T_pBEN1_BST  GGAATTTAATAATAATGTTTGTAATAAATGGATAAAGAATGTGAATTATTTATATAAATG
Col-0_BST         GGAATTTAATAATAATGTTTGTAATAAATGGATAAAGAATGTGAATTATTTATATAAATG
Col-0_no_BST      GGAATCCAATAACAATGTTTGTAATAAACGGACAAAGAATGTGAATTATTCACATAAATG
                  *****  ***** *************** *** ***************** * *******

ben1_O_pBEN1_BST  ATTTGTAAAATGTGAGTATTTGATTATAATGGATATAATAAAATAAAAATTGTAATAAAG
ben1_T_pBEN1_BST  ATTTGTAAAATGTGAGTATTTGATTATAATGGATATAATAAAATAAAAATTGTAATAAAG
Col-0_BST         ATTTGTAAAATGTGAGTATTTGATTATAATGGATATAATAAAATAAAAATTGTAATAAAG
Col-0_no_BST      ATCTGTAAAATGTGAGCATTTGATTATAACGGATATAATAAAACAAAAATTGTAATAAAG
                  ** ************* ************ ************* ****************

ben1_O_pBEN1_BST  AGAGAAGAGGGTAAAATGGGGAATATGTTATGTGGATAGGTGTGAGAGAGTAAATGATGT
ben1_T_pBEN1_BST  AGAGAAGAGGGTAAAATGGGGAATATGTTATGTGGATAGGTGTGAGAGAGTAAATGATGT
Col-0_BST         AGAGAAGAGGGTAAAATGGGGAATATGTTATGTGGATAGGTGTGAGAGAGTAAATGATGT
Col-0_no_BST      AGAGAAGAGGGCAAAACGGGGAACATGTCACGCGGATAGGTGTGAGAGAGTAAATGACGT
                  *********** **** ****** **** * * ************************ **

ben1_O_pBEN1_BST  AGGTTTTGTTATTTAAGAATTTTTTTAGAGAGGTTTTTATATAT-AGNNG----------
ben1_T_pBEN1_BST  AGGTTTTGTTATTTAAGAATTTTTTTAGAGAGGTTTTTATATATTAGAAGAGAGAGAAAG
Col-0_BST         AGGTTTTGTTATTTAAGAATTTTTTTAGAGAGGTTTTTATATATTAGAAGAGAGAGAAAG
Col-0_no_BST      AGGTTCCGTTATTTAAGAACCCTTCTAGAGAGGTCCC-ACACACCAGAAGAG--------
                  *****  ************   ** *********    * * *  **  *

ben1_O_pBEN1_BST  -------------------
ben1_T_pBEN1_BST  AGATAGAGAGAAAATGGTG
Col-0_BST         AGATAGAGAGAAAATGGTG
Col-0_no_BST      -------------------
```

(B)

```
ben1_O_Ex2_BST  GGTGGTGTTTGAGTTTGGTGGGAAGAATGGGTTAGAGGTTGTGATGTTTGTTATTTTTTT
ben1_T_Ex2_BST  GGTGGTGTTTGAGTTTGGTGGGAAGAATGGGTTAGAGGTTGTGATGTTTGTTATTTTTTT
Col-0_BST       GGTGGTGTTTGAGTTTGGTGGGAAGAATGGGTTAGAGGTTGTGATGTTTGTTATTTTTTT
Col-0_no_BST    GGCGGCGCTTGAGTTCGGTGGGAAGAATGGGTTAGAGGTTGTGACGCTCGTTATTCCTCT
                ** ** * ******* **************************** * * ******  * *

ben1_O_Ex2_BST  TGTTGTTGGATTTTTTATATTTTTGTTGTTGTTTTTTTTTGTTTTTATATTTTTTGTTAT
ben1_T_Ex2_BST  TGTTGTTGGATTTTTTATATTTTTGTTGTTGTTTTTTTTTGTTTTTATATTTTTTGTTAT
Col-0_BST       TGTTGTTGGATTTTTTATATTTTTGTTGTTGTTTTTTTTTGTTTTTATATTTTTTGTTAT
Col-0_no_BST    CGTCGTCGGACCTTTTATATCTTCGTCGTTGCCTTCCTCCGTCTTCATATCTCTCGCCAT
                 ** ** ***  ******** ** ** ****  **  *  ** ** **** * * *  **

ben1_O_Ex2_BST  GTTTTTTG/GTATTTAA
ben1_T_Ex2_BST  GTTTTTTG/GTATTTAA
Col-0_BST       GTTTTTTG/GTATTTAA
Col-0_no_BST    GCTCTTTG/GTACTTAA
                * * **** *** ****
```

Figure S3 Genomic DNA in the *BEN1* Promoter and *BEN1* 2nd Exon-Intron junction does not show any methylation in both the pre-triple *ben1-1* and the *bas1-2 sob7-1 ben1-1* triple-mutant lines. (A) *BEN1* promoter DNA was amplified using primers pBEN1-BS-F and pBEN1-BS-R from the bisulfite converted genomic DNA from the Col-0, pre-triple *ben1-1* and the triple-mutant lines. (B) *BEN1* DNA from the 2nd Exon-Intron junction was amplified using primers BEN1-E2-BS-F and BEN1-E2-BS-R from the bisulfite converted genomic DNA from the Col-0, pre-triple *ben1-1* and the triple-mutant lines. BST= bisulfite treatment. ben1_O= pre-triple *ben1-1*, and ben1_T= bas1-*2 sob7-1 ben1-1* triple-mutant.
