# Supplementary material for: The ben1-1 Brassinosteroid-Catabolism Mutation Is Unstable Due to Epigenetic Modifications of the Intronic T-DNA Insertion
Source: G3 (Bethesda). 2013 Sep 1;3(9):1587–95. doi: 10.1534/g3.113.006353 (PMC3755919; doi:10.1534/g3.113.006353)
Supplement: Supporting Information [file supp_g3.113.006353_FileS1.pdf]

File S1

Bisulfite *BEN1* 2nd exon raw sequences

ACCAGAAGAGAGAGAAAGAGACAGAGAGAAAATGGTGAGAGAAGAACAAGAAGAAGATGACAACAACAACAACAACAACGGAGG
AGGAGAGAGAAAGCTGCTTGTGGCTGATGAAACGGTGCCGTCTTTGTTGGACGAAACGGGGTTGGTTTGTGTAACGGGAGGTTCTG
GTTTCGTTGCTTCTTGGCTCATCATGCGTCTCCTTCAACGTGGCTACTCCGTTCGGGCCACGGTTCGAACCAACTCAGGTTCGAGTTAA
TTAACTTCACTTTCTCTTTACTTATACACTTTGAATCATCATCACTGATTAAGCAAATGCCAATAAAATTATTGTTGTCATATATGGCTGA
TTGGTCCGTTTTAACATAAAGTACATAAATAGAAAGATAGATTATAGTTTTTTTTTTATATATAGAGAGAGATAACATTTAAAGTTGAT
TTTTGGATCGACAATTAAAAATATATATATTTTTATGGATGTGAAAAATTTATTGTACTTGGCTGGAATTATCTGTCAGTTTGTAGGAT
CTTGAAGCTTCATAGCTATGTAAATATGTCATGTTTCCATGCATCTTCTATAGAAAGTCAACCATATATTTTATGATCAATAACTAATAA
GTAATCACGGACCATAAGATATAAATGAAGTTAATAATTCATAAGTTTGTTTATTTTATGCATATTACAGAAGGGAATAAGAAAGATA
TAAGCTACCTAACCGAGCTACCATTTGCATCGGAGAGGCTTCAAATATTCACCGCCGATCTCAACGAACCGGAGAGCTTCAAACCGGC
AATCGAAGGATGCAAAGCCGTATTCCACGTGGCACATCCTATGGACCCAAACAGCAACGAAACAGAAGAGACCGTTACGAAACGTA
CTGTGCAAGGTCTCATGGGGATACTAAAGTCGTGTTTGGACGCTAAAACCGTGAAACGTTTTTTCTACACGTCAAGCGCCGTGACTGT
TTTCTATAGCGGTGGAAACGGCGGTGGAGGAGGCGAAGTAGACGAGAGCGTTTGGAGCGACGTCGAGGTGTTTAGGAATCAAAAG
GAGAAGAGGGTGAGTAGCTCTTACGTCGTGTCGAAAATGGCGGCGGAGACGGCGGCGCTTGAGTTCGGTGGGAAGAATGGGTTAG
AGGTTGTGACGCTCGTTATTCCTCTCGTCGTCGGACCTTTTATATCTTCGTCGTTGCCTTCCTCCGTCTTCATATCTCTCGCCATGCTCTT
TGGTACTTAATTCTCATATCTAATAAGTTTTTTATTTTATTTTTCTCCATGATTAAACTAAAGCTGAAAATTAATAATTTTAAAAACTTAA
CCACTTTGATTACGTATACGTTATATATAGGTATTAATTTTTAAAACTAACCAAACCTTTTATTTCTCAAATAAAGTAAAGTAAATTCAT
AGTACTAGCCTACTAGCTAGCTTGTACTC(**insertion**)TAGTAGTAGTTAAATGGTTTATGGTTGGTTCACCAAGTATCTTGGGAGATCA
TAGCTCGAATTTTCTCAAAAAAAAAATTTTTGAACAAATGCTCGAATTTTCTCATTTATACTTTTGTTTTAAATGAAAAAAATATTCTGC
TCAGGCTTAGTAGATTTATGTTTGGACATAGTGGCTATTGAAAATGTTAGAATTCGAACCAAAATATGTATATTTTATACGAAAAAAC
AGAACTAAAATCCTAATTACTATACGTAACTAATAAAACTCTTTTCTTTTGCTTATAACCTACGTGCGTGGAATTTTTGAGTTATTTAGA
ACGAATTTTGTGTGTGTTAAACTCGTGTAAACATGTGCTAATAATATTCACAAATAGTGTTTTGAGTAATAATCACTATTCACTATAGT
AATATTACGTTTTTTTATGAAGGAAATTACAAGGAGAAGTATTTGTTTGATACTTACAATATGGTGCATATTGATGATGTGGCAAGGG
CGATGATATTTCTATTAGAAAAGCCAGTTGCAAAAGGTAGATATATTTGTTCGTCGGTGGAGATGAAGATCGATGAGGTCTTTGAGT
TTTTGTCTACGAAATTTCCTCAGTTTCAGCTACCTTCAATAGAGTATGTCATTTTTTACTAGTATAATTAATTTCACGTGTAAATATTCTA
TTGTTTTTTTCCAAGAGTTTTTATTATGTTCTTTTTTTGTGTGGGTACGTAAAGTTTGAATAAATATAAAGTAGAGAAGAGGATGGGTC
TTTCATCGAAGAAGCTGAAAAGTGCCGGGTTCGAATTCAAGTATGGAGCCGAGGAAATATTCAGTGGAGCTATAAGGAGCTGTCAA
GCAAGGGGATTTCTTTAAATTCTTATGGGATTTTTGTGTTAAAATTTGTATATGAATTGAAAATATGTTTTTTCTTTATTGGCCATTTCC
GTTTTGTAACATGTTCTAGTTACTTGTTGACGAAAAAAAAAAGAAAGTGTTTTGATTCAG

BLUE=Exons; Black=Introns

Primers:

BEN1-E2-BS-F: TTAGGAATTAAAAGGAGAAGAG

BEN1-E2-BS-R: AATTAAATACCAAAAAACATAAC

Replicate 1

>PK_329142-505_11_BS3_E04.ab1/>Col-0_BST
NNNNNNNNNNNNGNTGTGTTGANTGGTGGTGGAGATGGTGGTGTTTGAGTTTGGTGGGAAGAATGGGTTAGAGGTTGTGA

TGTTTGTTATTTTTTTTGTTGTTGGATTTTTTATATTTTTGTTGTTGTTTTTTTTTGTTTTTATATTTTTTGTTATGTTT
TTTGGTATTTAATTAATCCANCCGCTNTCNNAGCAACTTGNCCCNNNNACCCCCTCGAACGNCTCNNNTCCCCCCCTCCC
CGGCCCCCCAGCTACNAACNCNCTCATCCCNAAGGCTCCANCTAAAACCGTGACTNNCTNCCGTCCCAATTAAGAANGTG
TCTCNNTAAACAAANNTGCNCCCCNNANGACCACCGGTGTCAAACACTTCACTCNTNNAACCTGAAGCCAAANAATCATN
GCCTCNNTTNTGACCCNAANCTCGGAGGGACGCCCNTNATATNNNANCCTTCCCCNCTCCTCTCCCCCNCTTACCCCGAN
ACNNAANCCCGACTGCCCCNTCNNACNTTTCTCAGCCTCCGACAGCGACGCGTATTGATCTCCNCTTTTAACTCCCNCTC
ACNCACTCTTTNGGGTCATNNACCCCNCTNCNCCNNNNNNTNNAGTTTGTAATTATCTCAGGCCCA

>PK_329142-506_12_BS3_F04.ab1/>ben1_O_Ex2_BST
NNNNNNNNNNNNGNTGTGTTGANTGGTGGTGGAGANGGTGGTGTTTGAGTTTGGTGGGAAGAATGGGTTAGAGGTTGTGA
TGTTTGTTATTTTTTTTGTTGTTGGATTTTTTATATTTTTGTTGTTGTTTTTTTTTGTTTTTATATTTTTTGTTATGTTT
TTTGGTATTTAATTANCCAACNCNACNCANNGCCNNTNNACCCCATAACCCCCNCNANNCTNNCTCGTACNCCCCCCNCC
NGNCCCCCTCNCCNNGAACNNCCCGGCCCCGACGGGTCCATCNTANGNCNCGACGGCCCGCGGTGCCAGATANCCAATGG
GCTNCCNAATGAAACNCAAACNCCNCAGGNCCGAGCAGNTCAAAACGNCNNCCATCCCACNGNGAAGGACGAACANCACN
NANNCTCTATCTGGCCNCCCNGTNNGCCNNNGNNCGCTCACNGTCCACCACCCNACCNCAACCCNCCTATNCCNATCAAG
ACACNCCNCNCNACCTGCGNCCTGTCACTCCCAGGCNCCCNACANCGCCGNCTAGNGCGCTCTCCTCNAGCTCCCCCCAC
ATCNNTTCTGAGNTACNCANCCACCTGCNCCTCCCCCNCGATTNCCATNACAANAGCCNAT

>PK_329142-507_13_BS3_G04.ab1/>ben1_R_Ex2_BST
NNNNNNNNNNGNTGTGTTGANTGGTGGTGGAGATGGTGGTGTTTGAGTTTGGTGGGAAGAATGGGTTAGAGGTTGTGATG
TTTGTTATTTTTTTTGTTGTTGGATTTTTTATATTTTTGTTGTTGTTTTTTTTTGTTTTTATATTTTTTGTTATGTTTTT
TGGTATTTAATANCCAANCGCCTTCACAGCAACCTGGCCCCAAAACCCCTTCGAATGCCTCTCNTCCAACCCCCTCCTCT
CCCCNACNCTANNNNCNACCCNACTCCNGCGGCTCCANCCAACANNCCTTCCNCCNNCTCCCCCATCCTNANCCGGCCTT
CNCCNNNNAACATGATCCNCNCANGGCCNANGGCGNCNAANACCGCATCCCTCCATGGNNGAAGCTTGACCATCACNGAC
TCAATATCTGACTCCCAGCCCGGAGAGAGGGCCGCAACNTCACCCCACCCGCCNNCAACCCCCCATNNCTTCCTCGATAC
NGATCCATGNCTGCGACTTGCCTCTTCCCTTGAGTGCNATCGCGGTGCGTAGTGATCTACTCCTCCNACTTCGCCCTGCA
CACACTCATTTNNTTTACTANNATGATCNNNCTNNTCCCNNCNCNNCNNCNAATTATCNCAAGGCACCATTNNAAAANNN
AA

>PK_329142-508_14_BS3_H04.ab1/>ben1_T_Ex2_BST
NNNNNNNNNNTNGNTGTGTTGANTGGTGGTGGAGATGGTGGTGTTTGAGTTTGGTGGGAAGAATGGGTTAGAGGTTGTGA
TGTTTGTTATTTTTTTTGTTGTTGGATTTTTTATATTTTTGTTGTTGTTTTTTTTTGTTTTTATATTTTTTGTTATGTTT
TTTGGTATTTAANNNNCCCNCCCNNNCNGCCNCCCGNCCCCNANTCCCCNTCGNANGCNNCTCNTCCTCCCCCCTCCTCN
CCCCACTCTTNCCCCCCCCCGACCCCNACGGCTCCANCNNACGATCNTTCCCCCCNCNCCNCCTTCGCAGANGGGNNAGN
CNNCACCNAACATGATCCCCCCAGGGCCAAAGGCGTCAAAGACAGCTTCCCNGCANGGNNGAAGNATGACAATCACCCAC
TCAATATCGGACCCCCAGCCCGGANAGAGGCCCCCAACTTCACCCCCCCCGCCCNCGACCCCCCTNTNCGTTNCCCGACC
CTGANNCACGGNCCCGACTTGCCTCTTCCATTGAGTGAAATCGCGCTGCGTACCGACCCACTCCTCACACCTCNCCCGCA
CACTACNTCCGNTTTACCAANNNNATCCGTCNNNCCCCTCANNTCNNCCNATTATCACCAGGCCCATTNTNANNNNNNNN
N

>PK_329142-519_11_BS4_E06.ab1/>Col-0_BST
NNNNNNNNNANNNAAAATATAAAAAATCCAACAACAAAAAAAATAACAAACATCACAACCTCTAACCCATTCTTCCCACC
AAACTCAAACACCACCATCTCCACCACCATTTTCAACACAACATAAAAACTACTCACCCTCTTCTCCTTTTAATTCCTAA
AANNGGNNGNNNACNNNANGGNNNTGNNTNNNCNNATACTNCTCCGGGNGAGCGAGACTACGCTATNTGNTGAGAGGAGT
TGTTNGAGCGAAATNCCATNANAAAAAGGACAT

>PK_329142-520_12_BS4_F06.ab1/>ben1_O_Ex2_BST
NNNNNNNNNANNAAAAATATAAAAAATCCAACAACAAAAAAAATAACAAACATCACAACCTCTAACCCATTCTTCCCACC
AAACTCAAACACCACCATCTCCACCACCATTTTCAACACAACATAAAAACTACTCACCCTCTTCTCCTTTTAATTCCTAA
AAANGTANGNNGAGGGCTNGNGNGGTCAGTGTGACCATCTTCTTCTGGAGGACGTGCCAAAANTTTACTCCTAANCNAGG
TGATTTNCGAGGGCCACCCCATGGNGAANCNCATCGAGTTGAGGGGCATCCTCTTCNAGGAGGACAGAAANNTCN

>PK_329142-521_13_BS4_G06.ab1/>ben1_R_Ex2_BST
NNNNNNNNNNNANCANNNAAAATATAAAAAATCCAACAACAAAAAAAATAACAAACATCACAACCTCTAACCCATTCTTCC
CACCAAACTCAAACACCACCATCTCCACCACCATTTTCAACACAACATAAAAACTACTCACCCTCTTCTCCTTTTAATTC
CTAAAANNNNGANGGGNGTANGGGNNGANCTTGTCCTACATNCNTCGGGCNCCTGCTNGACTCCANCNNCTGGGCCCCG
TGTTTTCCGGGCNATCCCATGNACAATAGNNCACCGAGTACCGGAATGTTCCCGTCCCANGCTTGAACCNGTCNGATCNC

ATTGGTGAANACNANATTACGAACANTCTCNAGTCANCTATTTTNGTGTTACGGACTAANAGNATCANNGTANNCTTCTA
AATTGTCCACNACAACCNAGNANGTTCCNCCACGGTCGCTCATCGGCCGCCGGCTACCCTCANNCTGCATACAGACTGAT
TGGATGACNCAACATCCTNNNTTTGATGAAGGCGATATTCNAGATTAGNTACNTAATGCTNANTCTTAANTCTCTCANAC
TGNTGCATTGGGGACCNTAANGCNCATGCTCTCTNTNCCTAGNCGAACCNACTATAACCTTCNTCTGN

>PK_329142-522_14_BS4_H06.ab1/>ben1_T_Ex2_BST
NNNNNNNNNNNNNNNANNNAAAAATATAAAAAATCCAACAACAAAAAAAATAACAAACATCACAACCTCTAACCCATTCT
TCCCACCAAACTCAAACACCACCATCTCCACCACCATTTTCAACACAACATAAAAACTACTCACCCTCTTCTCCTTTTAA
TTCCTAAATNNGCCNGNGGGTTNGNGGGGNGGGGNCGGTCTTCNTCGGGATGGGNCNGACTNACNNGCTGTGAANGTGGT
TTCGGGNNATACNCCCNGGAGAACCACATCGCTCTGAGGGGCATCCTCTTGGAGGANANCGAAACGTCCTGGGGGAGNNG
TGGAGTACAANNNGANNNACCACAGCNTTANCNTGNTGGCGGACAAGAATAAGGGTNGCATTTTGTTGAACTTCATGATC
CACCTGTACTCGAAGGCTGGTGTTGTAGCTNANCNACCCNTGCCTACAGCTCAGCCCNNGGANNGACCGGCCCTGGTGGC
GGCCCGAACTNNTTNCCTGATC

```
ben1_O_Ex2_BST  GGTGGTGTTTGAGTTTGGTGGGAAGAATGGGTTAGAGGTTGTGATGTTTGTTATTTTTTT
ben1_T_Ex2_BST  GGTGGTGTTTGAGTTTGGTGGGAAGAATGGGTTAGAGGTTGTGATGTTTGTTATTTTTTT
Col-0_BST       GGTGGTGTTTGAGTTTGGTGGGAAGAATGGGTTAGAGGTTGTGATGTTTGTTATTTTTTT
Col-0_no_BST    GGCGGCGCTTGAGTTCGGTGGGAAGAATGGGTTAGAGGTTGTGACGCTCGTTATTCCTCT
                ** ** * ******* **************************** * * ******  * *

ben1_O_Ex2_BST  TGTTGTTGGATTTTTTATATTTTTGTTGTTGTTTTTTTTTGTTTTTATATTTTTTGTTAT
ben1_T_Ex2_BST  TGTTGTTGGATTTTTTATATTTTTGTTGTTGTTTTTTTTTGTTTTTATATTTTTTGTTAT
Col-0_BST       TGTTGTTGGATTTTTTATATTTTTGTTGTTGTTTTTTTTTGTTTTTATATTTTTTGTTAT
Col-0_no_BST    CGTCGTCGGACCTTTTATATCTTCGTCGTTGCCTTCCTCCGTCTTCATATCTCTCGCCAT
                ** ** ***  ******** ** ** ****  **  *  ** ** **** * * *  **

ben1_O_Ex2_BST  GTTTTTTG/GTATTTAA
ben1_T_Ex2_BST  GTTTTTTG/GTATTTAA
Col-0_BST       GTTTTTTG/GTATTTAA
Col-0_no_BST    GCTCTTTG/GTACTTAA
                * * **** *** ****
```

Replicate 2

>PK_329142-511_17_BS3_C05.ab1/>ben1_O_Ex2_BST
NNNNNNNNNNNNGNTGTGTTGANTGGTGGTGGAGATGGTGGTGTTTGAGTTTGGTGGGAAGAATGGGTTAGAGGTTGTGA
TGTTTGTTATTTTTTTTTGTTGTTGGATTTTTTATATTTTTGTTGTTGTTTTTTTTTTTATTTTATATTTTTTGTTATGTTT
TTTGGTATTTAATTANGAANCNTATGGACTGGACCCCNACCCNCTTCTCCGAANCGCNNTCCCNNCNTNNTCTGNTGAAC
CTACTTCTCTGCGGCANCCACCTNATTAANCCCNCCNNNTCNATATGCCCCTNACCCNCCTNCAAANACTATANCCTCNG
GGNGCCCCNGGTCCCTNTCCTNNTGCCNGNCCNCCACTTNTCCNCNNCCNCNTACTNNNAATATGCNCTNNTGGTATATT
ACTAGNATATNACCCNNNNTATTNNTGCNT

>PK_329142-512_18_BS3_D05.ab1/>ben1_R_Ex2_BST
NNNNNNNNNNNNTGTGTTGANTGGTGGTGGAGATGGTGGTGTTTGAGTTTGGTGGGAAGAATGGGTTAGAGGTTGTGATG
TTTGTTATTTTTTTTGTTGTTGGATTTTTTATATTTTTGTTGTTGTTTTTTTTTGTTTTTATATTTTTTGTTATGTTTTT
TGGTATTTAATAAATACCNNNNNTTTAAGGATGTTTTTGAAACGGTCTNCTTCGGGTCGGGGTCCCGCCGCTGCTTNGTA
AACGTAGTTNGTNNACCNCNCCTCCCTNGNACCACGCCANATCNACNTGTTCCTNACTCTCCACCAAGNN

>PK_329142-513_19_BS3_E05.ab1/>ben1_T_Ex2_BST
NNNNNNNNNNNTGNTGTGTTGANTGGTGGTGGAGATGGTGGTGTTTGAGTTTGGTGGGAAGAATGGGTTAGAGGTTGTGA
TGTTTGTTATTTTTTTTTGTTGTTGGATTTTTTATATTTTTGTTGTTGTTTTTTTTTGTTTTTATATTTTTTGTTATGTTT
TTTGGTATTTAATTAATACCCACCAATTTCGGANNNNNNTTNNCNNTCTTCTTCNGGGNGGNGCGANTGCTCTNCTTGTT

GTGNTTTTTTTTTGAGTGCCACCCCCTTTTTAATACGACATCTCTTTNTGTATCGNANTCGTCTTCTTNTTGN

>PK_329142-514_20_BS3_F05.ab1/>ben1_T_Ex2_BST
NNNNNNNNNNGTTGTGTTGANTGGTGGTGGAGATGGTGGTGTTTGAGTTTGGTGGGAAGAATGGGTTAGAGGTTGTGATG
TTTGTTATTTTTTTTGTTGTTGGATTTTTTATATTTTTGTTGTTGTTTTTTTTTGTTTTTATATTTTTTGTTATGTTTTT
TGGTATTTAATAANNNCCNNCCGCTACGTCCAGGAGCNNACCNTCTNTCTCCGGACCACGGCGACTCCGCTACCTGCTCC
GACGTGTTNTTCGAGGGCCACACCCTGTTGAACCGCNTCNCGCTGAATGGTACCNACTTCNNNCATGACNGCNACATCCT
GGGGCTAANNTGGANTNNCCTACNACAGCCACAANNTCTATATCCTGTTTCNATTCNNTGAAGAACAGCANCNCNGTGAA
CTTCTNNATCCNCCNCNCNCNNCNCTAAGGTTCCNTCTNANNNNNNACCNCTTNCTNCNCAACNCTCCCCTCGTNGACGG
NCCCGTGNTGCTGCCCGCAACCACTATCTGANCACCCCTNCCTCCCTGAGTTNTATTTTANNNATN

>PK_329142-525_17_BS4_C07.ab1/>ben1_O_Ex2_BST
NNNNNNNNNNNNNNNNNNAAANTATAAANANATCCAACANNNNAAAAAAATAACAAACTTCCCATCCTCTAACCCATTCTTC
CCACCAGACTCAAACACCACCATCTCCACCACCATTTTCAACACAACATAAAAACTACTCACCCTCTTCTCCTTTTAATT
CCTAAAANGTNNNNNCCGNCNTGGNCNGNNNCGGCATGNATATNNTGCGGGTGGNGCTGACCGTGCTGTNCTGNNTGANC
GTTTNGTTGATCTGCNCCCTGGCCATGNNGAANGNGACCTTGTTCNACTGTAACCNTGTNCTGCTGCNACAACCGNNNNG
GGACCCACNGTGTGTGAAATNGCTGGTGCATAGNGCCGGCTAAATGCACTNCTCGTTGGNNGACNACTCCCATGTTGGGC
CCCAAGCCACCNNTGCTTGATGACTTCTCNCCGTGCTCGCCCTGCTGGTNGCTCTGTTTGNNCTGCTGGTTGANTGNCCG
GNCCNNCACCGGTACCNACTTGGNACAAACAGACNGNATATGACGNCANTCTCTTACANGCTCCATTNGTTNNGAATTGN
GTCTTTNTCTNNCTNTCTCTCAACCGTGTTNTACTNNCCATTCGCTCGTGCNGGGCTCCTGCCACCTCGNGATGTCGGAC
TNCNANAATAATGTANTTTANCNNTGCCTATTNATNTCCTNTGCGNTAAACCNCCTGCTACGCCCAGGTGG

>PK_329142-526_18_BS4_D07.ab1/>ben1_R_Ex2_BST
NNNNNNNNNNNANAANAAAAATATAAAAAATCCAACAACAAAAAAAAATAACAAACATCACAACCTCTAACCCATTCTTCCC
ACCAAACTCAAACACCACCATCTCCACCACCATTTTCAACACAACATAAAAACTACTCACCCTCTTCTCCTTTTAATTCC
TAAAANGTAAAGGGTGATGGNNTCNGCCGGCANGGCCATCCTNTGCGAGAGGCGGACCCTGTGCTGTTGAGTGAACGAAC
TGNTGAGCTGCGCCCTGCCTNNTAGGNATGTGANCNCCTTCNNNGCCAACNCNNNGNNCTAGTNCCATAACNNCATCNAG
CCCCTGNGGATGAGCTNNGNGGGGGGAGCANCGNCCCTATTANCACTANTCGTTAGATCANGACTCGNATGTGGTACGAG
ATTACACCGCTGCTTGAGGGCTGCCCATGATCNGCGTCCTGGAGGCCCTGCTNNATCGTTGNTGTACATANNAGGCCTCT
CNTGCACCGGCTGCTGANAAGAAACAGGCTCCCTGGACAGGCTGGNACTAATGNCCAGGATCGTGTTCCTGATCAAATGG
ATGTTTATCTGGCTCTCTCTGTGCTGTCCTGCTCACTCCATTCGCGCGNGCTTGGACNCCCCCNTGNTGGNAGGNNGTNN
NAAAGNAAACTGNNNNCNANCCNTGCCTGNNCTGNTCCNGGGNCNNNATGCTGCTGNTNNNGNNAGN

>PK_329142-527_19_BS4_E07.ab1/>ben1_T_Ex2_BST
NNNNNNNNNNNNNNNNAAAAATATAAAAAATCCAACAACAAAAAAAAATAACAAACATCACAACCTCTAACCCATTCTTCCC
ACCAAACTCAAACACCACCATCTCCACCACCATTTTCAACACAACATAAAAACTACTCACCCTCTTCTCCTTTTAATTCC
TAAAANGGGTAGAGCGNCCATGATCTCNGCCGGCGGGNNGATCCTGGGCGGGGTGGGGACCCTGCGCGGCTGGGTGAACG
GGCTGGGGTGATGCCCCCTGCCGNTGTGGTAGGTNACCGCCTTGTGTGCCTTAATTATCGGGGTGGATGGNTNGGTGTGG
GAAGGCCAGNGGATGAGNTGTGCGGTGCNCCGCNCCGGNCAGAGTCCTNGCANGGTGNGGACAGCAGCNNGGATGTGCCC
CTGGACGCGCAGGCCGCAAGAGGACTGCGCGTGATCACCCNGCTGGTGGCCCTGTTCNNNCTGATGGTGTNCATGGCNGC
NCTCATNTGCACCACCAGNGCGCAGGACAAAGACAGCCAGGCCAGGCTGGCGCTGACCACCGGCCTCGTGTTCGTGATCN
NNGGGTGCTGAACNTGATCACGCTGAGCTGGGCCGCCNNCTCCATGATCACGTACTTCTNTATTGCCGTGTTGGNNTANN
GNAGNANNANNAAGNNGNNGTCNTACACGTNNCTGTGCTGGTGCNCGGTCGTCAGNTGNTGCTNNNGNNTGGTTGGNCNN
NNGTTGNNTCGNNNTTNGCANNANGNNNNNGCCCANCANACNGGCNCCATCTACACGANCANGTCGCAGAGCCGCCTNNN
GANGNCCCANGGNANNNNGNNNNGGNTNNN

>PK_329142-528_20_BS4_F07.ab1/>ben1_T_Ex2_BST
NNNNNNNNNCNANNANNAAAATATAAAAAATCCAACAACAAAAAAAAATAACAAACATCACAACCTCTAACCCATTCTTCC
CACCAAACTCAAACACCACCATCTCCACCACCATTTTCAACACAACATAAAAACTACTCACCCTCTTCTCCTTTTAATTC
CTAAAANNTGGCTGGGNTGATNTGGNNGGTGACGGAATGGTCTGCGCCGGCATGCCCATCCTGGGCGTGGTGGTGANCCT
GCTGGGCTGGGCGAACGGCCTGGTGAGCTGCGCCTTGTCTATGTGGAATGTGACCTCCTTCNTCNGCAACAACCTCNTGG
TGGCCCAGGTGGTGTGGGAGGGCCTGTGGATGANCTGNGNGGTGCANANCACCGGCGGATGCANTGTCNNGNGTAGGACC
CTCTGCTGGNNCTGCCCNAGGACCTGNANGANCAACAGCCCTGTGGNTGATCNNCCTGCTGGTGGNCCTGTTCGGCCTGN
TGGTGTACCTGGACGGCGCTAGGTGCAACAANTGCGTGCAGGATAACGATATCCACGNCAANCTGGTGGCNACTGGCGCN
NTCCTGTTTCGTGATCACGGCGAGCTGACCCNCATCNCNGTGNNCTNGAGCNNCGANCNTGCCNTCTGGGAGTCCTGNNC
ANGCTGNNGGTGNAGNCNCACACCNANGCANNTNNGCTGNNCGNNTGTACTTGNNNNGCANCTCNANCGNNNN
