# Supplementary material for: The ben1-1 Brassinosteroid-Catabolism Mutation Is Unstable Due to Epigenetic Modifications of the Intronic T-DNA Insertion
Source: G3 (Bethesda). 2013 Sep 1;3(9):1587–95. doi: 10.1534/g3.113.006353 (PMC3755919; doi:10.1534/g3.113.006353)
Supplement: Supporting Information [file supp_g3.113.006353_FileS2.pdf]

File S2

Bisulfite sequences of the *BEN1* promoter

ccattccatctcattcacgcgttttattttctctcattcatattaattatatattaatcacacaatcctaataatatcccttaatctatgcct
agaacatgcatggtattataattaatacattcgacaccactagaagccaaaagtatagtagtaaaaaaaagaagaagaagctggtttgtacgt
aagaatgaaaataacggaatccaataacaatgtttgtaataaacggacaaagaatgtgaattattcacataaatgatctgtaaaatgtgagca
tttgattataacggatataataaaacaaaaattgtaataaagagagaagagggcaaaacggggaacatgtcacgcggataggtgtgagagagt
aaatgacgtaggttccgttatttaagaacccttctagagaggttcccacacaccagaagagagagaaagagacagagagaaaATGGTGAGAGA
AGAACAAGAAGAAGATGACAACAACAACAACAAC

Primers:

pBEN1-BS-Fv: 5'-GTATAGTAGTAAAAAAAAGAAGAAGAAG-3'

pBEN1-BS-Rv: 5'-CTTRTTCTTCTCTCACCATTTCTCTCT-3'

(RC=reverse compliment)

Replicate 1

>PK_328409-528_5-R_Primer-D_A02.ab1/>Col-0_no_BST
NNNNNNNNNNNCTCTTCTGGTGTGTGGGACCTCTCTAGAAGGGTTCTTAAATAACGGAACCTACGTCATTTACTCTCTCA
CACCTATCCGCGTGACATGTTCCCCGTTTTGCCCTCTTCTCTCTTTATTACAATTTTTGTTTTATTATATCCGTTATAAT
CAAATGCTCACATTTTACAGATCATTTATGTGAATAATTCACATTCTTTGTCCGTTTATTACAAACATTGTTATTGGATT
CCGTTATTTTCATTCTTACGTACAAACCAGCTTCTTCTTCTTTTTTTTACTACTATAN

>RC_328409-528_5-R_Primer-D_A02.ab1/>Col-0_no_BST
NTATAGTAGTAAAAAAAAGAAGAAGAAGCTGGTTTGTACGTAAGAATGAAAATAACGGAATCCAATAACAATGTTTGTAATAAAC
GGACAAAGAATGTGAATTATTCACATAAATGATCTGTAAAATGTGAGCATTTGATTATAACGGATATAATAAAACAAAAATTGTA
ATAAAGAGAGAAGAGGGCAAAACGGGGAACATGTCACGCGGATAGGTGTGAGAGAGTAAATGACGTAGGTTCCGTTATTTAAGAA
CCCTTCTAGAGAGGTCCCACACACCAGAAGAG

>PK_328409-529_6-F_Primer-C_B02.ab1/>Col-0_BST
NNNNNNNNNNGTAGATGANATAATGGAATTTAATAATAATGTTTGTAATAAATGGATAAAGAATGTGAATTATTTATATA
AATGATTTGTAAAATGTGAGTATTTGATTATAATGGATATAATAAAATAAAAATTGTAATAAAGAGAGAAGAGGGTAAAA
TGGGGAATATGTTATGTGGATAGGTGTGAGAGAGTAAATGATGTAGGTTTTGTTATTTAAGAATTTTTTTAGAGAGGTTT
TTATATATTAGAAGAGAGAGAAAGAGATAGAGAGAAAATGGTGAGAGAAGAACAAGACNNCCTNCNNCCTTCCCTCCCCC
CCACCTATCCACATACCATTTTCCCCCTTTCACCCCCTTCTCTCTTTATNCAAATCTTTTTTGCTCATCNCCCNACNAAT
CAAATACCCCATTTTTCACATCNNTCTTCTAAATAATTCCATTCTTTATCCATTTTTCCCTCACATTAGGCTAATTCCTC
ACTNTTTTCTTTCCTTATACAAACNNCTTCTTCTTCTTTTNGGGTACNCNANACCAGCTNTTGCTNCCTTTAGTNNN

>PK_328409-530_6-R_Primer-D_C02.ab1/>Col-0_BST
NNNNNNNNNNCTTCNATATATATAAAAACCTCTCTAAAAAAATTCTTAAATAACAAAACCTACATCATTTACTCTCTCACAC
CTATCCACATAACATATTCCCCATTTTACCCTCTTCTCTCTTTATTACAATTTTTATTTTATTATATCCATTATAATCAA
ATACTCACATTTTACAAATCATTTATATAAATAATTCACATTCTTTATCCATTTATTACAAACATTATTATTAAATTCCA
TTATTTTCATTCTTACATACAAACCAACTTCTTCTTCTTTTTTTTACTACTATAN

>PK_328409-531_7-F_Primer-C_D02.ab1/>ben1_O_pBEN1_BST
NNNNNNNNNNNNNTGTNNATGANATAANGGAATTTAATAATAATGTTTGTAATAAATGGATAAAGAATGTGAATTATTTAT
ATAAATGATTTGTAAAATGTGAGTATTTGATTATAATGGATATAATAAAATAAAAATTGTAATAAAGAGAGAAGAGGGTA
AAATGGGGAATATGTTATGTGGATAGGTGTGAGAGAGTAAATGATGTAGGTTTTGTTATTTAAGAATTTTTTTNNAGAGG
TTCTTATATATTAGACNACAGAGCAAGAG

>PK_328409-532_7-R_Primer-D_E02.ab1/>ben1_O_pBEN1_BST
NNNNNNNNNNNNNNNCNNCTATATATAAAAACCTCTCTAAAAAAATTCTTAAATAACAAAACCTACATCATTTACTCTCTC

ACACCTATCCACATAACATATTCCCCATTTTACCCTCTTCTCTCTTTATTACAATTTTTATTTTATTATATCCATTATAA
TCAAATACTCACATTTTACAAATCATTTATATAAATAATTCACATTCTTTATCCATTTATTACAAACATTATTATTAAAT
TCCATTATTTTCATTCTTACATACAAACCAACTTCTTCTTCTTTTTTTTTACTACTATACANNN

>PK_328409-533_8-F_Primer-C_F02.ab1/>ben1_R_pBEN1_BST
NNNNNTNNGNNTGTANGATGANNTAATGGAATTTAATAATAATGTTTGTAATAAATGGATAAAGAATGTGAATTATTTAT
ATAAATGATTTGTAAAATGTGAGTATTTGATTATAATGGATATAATAAAATAAAAATTGTAATAAAGAGAGAAGAGGGTA
AAATGGGGAATATGTTATGTGGATAGGTGTGAGAGAGTAAATGATGTAGGTTTTGTTATTTAAGAATTTTTTTAGAGAGG
TTTTTATATATTAGAAGAGAGAGAAAGAGATAGAGAGAAAATGGTGAGAGAAGAACAAGAANANANACATNTTTACTCTC
TCACACCTATCCACATAANTATTCCCATTTTACCCTCTTCTCTCTTTATTAAATTTTTATTNNN

>PK_328409-534_8-R_Primer-D_G02.ab1/>ben1_O_pBEN1_BST
NNNNNNNNNNNCTTCNATATATAAAAACCTCTCTAAAAAAATTCTTAAATAACAAAACCTACATCATTTACTCTCTCACA
CCTATCCACATAACATATTCCCCATTTTACCCTCTTCTCTCTTTATTACAATTTTTATTTTATTATATCCATTATAATCA
AATACTCACATTTTACAAATCATTTATATAAATAATTCACATTCTTTATCCATTTATTACAAACATTATTATTAAATTCC
ATTATTTTCATTCTTACATACAAACCAACTTCTTCTTCTTTTTTTTTACTACTATAN

>PK_328409-535_9-F_Primer-C_H02.ab1/>ben1_T_pBEN1_BST
NNNNNNNNNNGTAGATGANATAATGGAATTTAATAATAATGTTTGTAATAAATGGATAAAGAATGTGAATTATTTATATA
AATGATTTGTAAAATGTGAGTATTTGATTATAATGGATATAATAAAATAAAAATTGTAATAAAGAGAGAAGAGGGTAAAA
TGGGGAATATGTTATGTGGATAGGTGTGAGAGAGTAAATGATGTAGGTTTTGTTATTTAAGAATTTTTTTAGAGAGGTTT
TTATATATTAGAAGAGAGAGAAAGAGATAGAGAGAAAATGGTGAGAGAAGAACAAGAAAACCTACATCATTTACTCTCTC
ACACCTATCCACATAACATATTCCCCATTTTACCCTCTTCTCTCTTTANNNANNTTTTATTTTATTATATCCATTANAAT
CAATACTCCATTTTCAAATCATTNATATAAATAATTCCNTTCNTTNNNCNNNGTTCCANACATTNTTNTTNNNT

>PK_328409-536_9-R_Primer-D_A03.ab1/>ben1_T_pBEN1_BST
NNNNNNNNNNNNNNNNTCTNNNNNNTATAAAAACCTCTCTAAAAAAATTCTTAAATAACAAAACCTACATCATTTACTCTC
TCACACCTATCCACATAACATATTCCCCATTTTACCCTCTTCTCTCTTTATTACAATTTTTATTTTATTATATCCATTAT
AATCAAATACTCACATTTTACAAATCATTTATATAAATAATTCACATTCTTTATCCATTTATTACAAACATTATTATTAA
ATTCCATTATTTTCATTCTTACATACAAACCAACTTCTTCTTCTTTTTTTTTACTACTATACCANNGNNNNGGCCGTGGNN
NGAGCGTNCGGCCTTTTTGNTGTTGCGTNCGGTCTGTTTTTTGTAACAACAATNGCGCCTANGCCATCTTCNATTTTCGG
NGNCCTTTANCTTGCCACGNCCCAGAGCNCCNCAGTAGCCGCCGNATCCNCCNNAAANNGGGNAGTCCACCCANNAGATG
AAGCTGATCGTTTCCCNCNACCCCNACGGGGGGCNCGNTNCCCAACTTTTGTTCCTGTTTNNGAGAGCNCANNTNANNAA
TTGAGACTGNNNNGGNCTGGCTGGTTCCCNGTTNAGATTNTAAACCGCACACTGTTCCCNATATCTCANGACCGGNAGNA
TATGNNTNAGCCCGNCGNANNNNATNNTTANNTAANTGNNCTAGNTTNTGCGGTTANTNCCACTTTCCNNACNNNGAGTC
CTTNNNNCNNNCTNCATTTTCTAACNTCTANCAATGNAGCNNGTTNTTGNAATACCTAAT

Sequence alignment:

```
ben1_O_pBEN1_BST  GGAATTTAATAATAATGTTTGTAATAAATGGATAAAGAATGTGAATTATTTATATAAATG
ben1_T_pBEN1_BST  GGAATTTAATAATAATGTTTGTAATAAATGGATAAAGAATGTGAATTATTTATATAAATG
Col-0_BST         GGAATTTAATAATAATGTTTGTAATAAATGGATAAAGAATGTGAATTATTTATATAAATG
Col-0_no_BST      GGAATCCAATAACAATGTTTGTAATAAACGGACAAAGAATGTGAATTATTCACATAAATG
                  *****  ***** *************** *** ***************** * *******

ben1_O_pBEN1_BST  ATTTGTAAAATGTGAGTATTTGATTATAATGGATATAATAAAATAAAAATTGTAATAAAG
ben1_T_pBEN1_BST  ATTTGTAAAATGTGAGTATTTGATTATAATGGATATAATAAAATAAAAATTGTAATAAAG
Col-0_BST         ATTTGTAAAATGTGAGTATTTGATTATAATGGATATAATAAAATAAAAATTGTAATAAAG
Col-0_no_BST      ATCTGTAAAATGTGAGCATTTGATTATAACGGATATAATAAAACAAAAATTGTAATAAAG
                  ** ************* ************ ************* ****************

ben1_O_pBEN1_BST  AGAGAAGAGGGTAAAATGGGGAATATGTTATGTGGATAGGTGTGAGAGAGTAAATGATGT
ben1_T_pBEN1_BST  AGAGAAGAGGGTAAAATGGGGAATATGTTATGTGGATAGGTGTGAGAGAGTAAATGATGT
Col-0_BST         AGAGAAGAGGGTAAAATGGGGAATATGTTATGTGGATAGGTGTGAGAGAGTAAATGATGT
Col-0_no_BST      AGAGAAGAGGGCAAAACGGGGAACATGTCACGCGGATAGGTGTGAGAGAGTAAATGACGT
                  *********** **** ****** **** * * ************************ **
```

```
ben1_O_pBEN1_BST  AGGTTTTGTTATTTAAGAATTTTTTTAGAGAGGTTTTTATATAT-AGNNG----------
ben1_T_pBEN1_BST  AGGTTTTGTTATTTAAGAATTTTTTTAGAGAGGTTTTTATATATTAGAAGAGAGAGAAAG
Col-0_BST         AGGTTTTGTTATTTAAGAATTTTTTTAGAGAGGTTTTTATATATTAGAAGAGAGAGAAAG
Col-0_no_BST      AGGTTCCGTTATTTAAGAACCCTTCTAGAGAGGTCCC-ACACACCAGAAGAG--------
                  *****  ***********   ** *********    * * *   **   *

ben1_O_pBEN1_BST  -------------------
ben1_T_pBEN1_BST  AGATAGAGAGAAAATGGTG
Col-0_BST         AGATAGAGAGAAAATGGTG
Col-0_no_BST      -------------------
```

Replicate 2

>PK_329142-503_7_pBEN1-Fv_C04.ab1/>ben1_O_pBEN1_BST
NNNNNNNNNNNNNNNNNNATGANATAATGGAATTTAATAATAATGTTTGTAATAAATGGATAAAGAATGTGAATTATTTAT
ATAAATGATTTGTAAAATGTGAGTATTTGATTATAATGGATATAATAAAATAAAAATTGTAATAAAGAGAGAAGAGGGTA
AAATGGGGAATATGTTATGTGGATAGGTGTGAGAGACTAAATGATGTAAGTTTTGTNANTCCTNCNTTTTGTCCNCCCCC
CTCTCCCCCCTCTNACCCCNCCCNCNCCNCTACAGAGGNCCNCCNCCTACCNCNNNCCCGACCNTCCCNCCCCAGTACCN
GTCNNATCCCCTNGACAANNCCCACTCCCCCNACGCCCTCCGGACCCNNCCCTCCNNNNNCTCNCTCNNANANCCATGCT
NCNCTCTNNNTGTCNNCCNCCCCCNNCNCCGAGCCACCCTCCTCCACCNCGCCCCCCCGCCCCCCCCCCCTCTGCCNCTTT
CCCATCGCNNANTGCCTGATGCCACATCCNTCTNNGCCACCCTCCAACTTCGCCGCGTCGTGCTCAACCNCCNCTCNCCC
CNCCCCCACCCTCTCTCNNGCCNCCTCCCACCCCCCNTCCCTNNCCAANNN

>PK_329142-504_8_pBEN1-Fv_D04.ab1/>ben1_R_pBEN1_BST
NNNNNNNNNNNNGTAGATGANATAATGGAATTTAATAATAATGTTTGTAATAAATGGATAAAGAATGTGAATTATTTATA
TAAATGATTTGTAAAATGTGAGTATTTGATTATAATGGATATAATAAAATAAAAATTGTAATAAAGAGAGAAGAGGGTAA
AATGGGGAATATGTTATGTGGATAGGTGTGAGAGAGTAAATGATGTAGGTTTTGTTATTTAAGAATTTTTTTAGAGAGGT
TTTTATATATTAGAAGAGAGAGAAAGAGATAGAGAGAAAATGGTGAGAGAAGAACAAGANNNCCCCCTNANNCCCGTCGG
GTCTCCTTANCNNATACCACCTCNCCGATGCCCCNCNGCGCCNTGANCNNGTCNNCTCTCNGAGNGGCNTGCNCCNCNNC
GCCCCNNTTGNGNCCNNNNACTCCGAGCNNNNNNCGCCCNCCCCGCCCCCCCNNCCCCCACCCTCTGCCTCGNATCTGAC
TCCTGANTGCCGGTGGCCAACCTCCCNCGAATGACNCCCTCCGACNGCGGCGCGTCGTGCTCGCCNCCACTCNCCCCCCC
CCGACACCNCNCTGGGNTCCGNCNCCCCCTGCCCCCCCNCN

>PK_329142-517_7_pBEN1-Rv_C06.ab1/>ben1_O_pBEN1_BST
NNNNNNNNNNNNNNNNNNNNNAAAACCTCTCTAAAAAAATTCTTAAATAACAAAACCTACATCATTTACTCTCTCACACC
TATCCACATAACATATTCCCCATTTTACCCTCTTCTCTCTTTATTACAATTTTTATTTTATTATATCCATTATAATCAAA
TACTCACATTTTACAAATCATTTATATAAATAGGTCACATTCTTTATCCATTTATTACAAACATTATTATTAAATTCCAT
TATTTTCATTCTTACATACAAACCAACTTCTTCTTCTTTTTTTTACTACTATACAGGNGCNGNNGGTGGTCNTGTAGGGN
CGGGGTGGCTGNCTGNNGCNGNTNGNNNNGGTGCGTACATTTACTAGGNNCCTANCNNNNGCGAGNCGCTGGCNGCGTAG
TCCTGNAGCGGCNGCGGCCCGGNGACCGCTGGTGGGCCGCGNCCTCGAGGGGGNTGTANANGGCCCNGANGATGANNGNN
CGGNCGNGCNGCNGNGGATCAGGNTNAACNNGCCNNTGATCANGGNNGGGATGNCGNNGGCCATCGCGNTN

>PK_329142-518_8_pBEN1-Rv_D06.ab1/>ben1_R_pBEN1_BST
NNGNGNNGNNNNNNNNNANGNNNNNNNNNNNTGCTNNAAAAAGGCTTAAATAACAAAACCTACATNATTTGNTCTCTNNNN
CCTATCCACATAACATATTCCCCATTTTACCCTCTTCTCTCTTTATTACAATTTTTATTTTATTATATCCATTATAATCA
AATGCTCACATTTTACAGATCATTTATATAGATAGTTNACATTCTTTATCCATTTATTACAAACATTATTATTAAATTCC
ATTATTTTCATTCTTACATACAAACCAACTTCTTCTTCTTTTTTTTACTACTATACN
