# Supplementary material for: The ben1-1 Brassinosteroid-Catabolism Mutation Is Unstable Due to Epigenetic Modifications of the Intronic T-DNA Insertion
Source: G3 (Bethesda). 2013 Sep 1;3(9):1587–95. doi: 10.1534/g3.113.006353 (PMC3755919; doi:10.1534/g3.113.006353)
Supplement: Supporting Information [file supp_g3.113.006353_FileS3.pdf]

**File S3**

**Bisulfite pNOS raw sequences**

GTTTACCCGCCAATATATCCTGTCAAACACTGATAGTTTAAACTGAAGGCGGGAAACGACAATCTGATCATGAGCGGAGAATTAAGGGAGTCACGT
TATGACCCCCGCCGATGACGCGGGACAAGCCGTTTTACGTTTGGAACTGACAGAACCGCAACGTTGAAGGAGCCACTCAG**CCGCGG**GTTTCTGGA
GTTTAATGAGCTAAGCACATACGTCAGAAACCATTATTGCGCGTTCAAAAGTCGCCTAAGGTCACTATCAGCTAGCAAATATTTCTTGTCAAAAATG
CTCCACTGACGTTCCATAAATTCCCCTCGGTATCCAATTAGAGTCTCATATTCACTCTCAATCCAAATAATCTGCACCGGATCTGGATCGTTTCGCATG
ATTGAACAAGATGGATTGCACGCAGGTTCTCCGGCCGCTTGGGTGGAGAGGCTATTCGGCTATGACTGGGCACAACAGACAATCGGCTGCTCTGA
TGCCGCCGTGTTCCGGCTGTCAGCGCAGGGGCGCCCGGTTCTTTTTGTCAAGACCGACCTGTCCGGTGCCCTGAATGAACTGCAGGACGAGGCAG
CGCGGCTATCGTGGCTGGCCACGACGGGCGTTCCTTGCGCAGCTGTGCTCGACGTTGTCACTGAAGCGGGAAGGGACTGGCTGCTATTGGGCGAA
GTGCCGGGGCAGGATCTCCTGTCATCTCACCTTGCTCCTGCCGAGAAAGTATCCATCATGGCTGATGCAATGCGGCGGCTGCATACGCTTGATCCG
GCTACCTGCCCATTCGACCACCAAGCGAAACATCGCATCGAGCGAGCACGTACTCGGATGGAAGCCGGTCTTGTCGATCAGGATGATCTGGACGA
AGAGCATCAGGGGCTCGCGCCAGCCGAACTGTTCGCCAGGCTCAAGGCGCGCATGCCCGACGGCGATGATCTCGTCGTGACCCATGGCGATGCCT
GCTTGCCGAATATCATGGTGGAAAATGGCCGCTTTTCTGGATTCATCGACTGTGGCCGGCTGGGTGTGGCGGACCGCTATCAGGACATAGCGTTG
GCTACCCGTGATATTGCTGAAGAGCTTGGCGGCGAATGGGCTGACCGCTTCCTCGTGCTTTACGGTATCGCCGCTCCCGATTCGCAGCGCATCGCCT
TCTATCGCCTTCTTGACGAGTTCTTCTGA

## Primers:

pNOS-BS-F (Primer A/pROK2-Fv): 5'-GGGTTTYTGGAGTTTAATGAGYTAAG-3'
pNOS-BS-R (Primer B/pROK2-Rv): 5'-CACTTCRCCCAATARCARCCARTCCCTTCC-3'

# Replicate 1

>PK_328409-501_1-F_Primer-A_A01.ab1/>*ben1*_O_pNOS_no_BST
CNNNNNNNNNNNANATTATTGCGCGTTCNNNGTCGCCTAAGGTCACTATCAGCTAGCAAATATTTCTTGTCAAAAATGCT
CCACTGACGTTCCATAAATTCCCCTCGGTATCCAATTAGAGTCTCATATTCACTCTCAATCCAAATAATCTGCACCGGAT
CTGGATCGTTTCGCATGATTGAACAAGATGGATTGCACGCAGGTTCTCCGGCCGCTTGGGTGGAGAGGCTATTCGGCTAT
GACTGGGCACAACAGACAATCGGCTGCTCTGATGCCGCCGTGTTCCGGCTGTCAGCGCAGGGGCGCCCGGTTCTTTTTGT
CAAGACCGACCTGTCCGGTGCCCTGAATGAACTGCAGGACGAGGCAGCGCGGCTATCGTGGCTGGCCACGACGGGCGTTC
CTTGCGCAGCTGTGCTCGACGTTGTCACTGAAGCGGGAAGGGACTGGCTGCTATTGGGCGAAGTGAACNCNTCAAGCACA
GCTGCGCAGGNNNNNCGTCNTGGCCAGCCNNATAGCCCCGCTNCTTCTCCTGNNNTTCNTTAAGGNNCCGGNANNGGNNG
NCTTGANAAAAAAGAACCGGGGGCCCNNGNNNNAAANCCGGAAACCGGGCGCNNNNAACCCCNNNNNNNNTTGTNNNNGCC
NNANAAANCNNAAANGCCTCCCCCCCNNGGGNNNNNANCNTGNTNNNNTNNNTTNTNTTCGNNNNNNNNNCNNATNNNAN
NNNTNN

>PK_328409-502_1-R_Primer-B_B01.ab1
NNNNNNNNNNNNNNNNNNGTCGAGCACAGCTGCGCAGGAACGCCCGTCGTGGCCAGCCACGATAGCCGCGCTGCCTCGTCC
TGCAGTTCATTCAGGGCACCGGACAGGTCGGTCTTGACAAAAAGAACCGGGCGCCCCTGCGCTGACAGCCGGAACACGGC
GGCATCAGAGCAGCCGATTGTCTGTTGTGCCCAGTCATAGCCGAATAGCCTCTCCACCCAAGCGGCCGGAGAACCTGCGT
GCAATCCATCTTGTTCAATCATGCGAAACGATCCAGATCCGGTGCAGATTATTTGGATTGAGAGTGAATATGAGACTCTA
ATTGGATACCGAGGGGAATTTATGGAACGTCAGTGGAGCATTTTTGACAAGAAATATTTGCTAGCTGATAGTGACCTTAG
GCGACTTTTGAACGCGCAATAATGGTTTCTGACGTATGTGCTTAACTCATTAAACTCCAAAAACNNNN

>RC_328409-502_1-R_Primer-B_B01.ab1/>*ben1*_O_pNOS_no-BST
NNNNGTTTTTGGAGTTTAATGAGTTAAGCACATACGTCAGAAACCATTATTGCGCGTTCAAAAGTCGCCTAAGGTCACTATCAGCTA
GCAAATATTTCTTGTCAAAAATGCTCCACTGACGTTCCATAAATTCCCCTCGGTATCCAATTAGAGTCTCATATTCACTCTCAATCCAAA

TAATCTGCACCGGATCTGGATCGTTTCGCATGATTGAACAAGATGGATTGCACGCAGGTTCTCCGGCCGCTTGGGTGGAGAGGCTAT
TCGGCTATGACTGGGCACAACAGACAATCGGCTGCTCTGATGCCGCCGTGTTCCGGCTGTCAGCGCAGGGGCGCCCGGTTCTTTTTG
TCAAGACCGACCTGTCCGGTGCCCTGAATGAACTGCAGGACGAGGCAGCGCGGCTATCGTGGCTGGCCACGACGGGCGTTCCTGCG
CAGCTGTGCTCGACNNNNNNNNNNNNNNNNN

>PK_328409-503_2-F_Primer-A_C01.ab1/>*ben1*_O_pNOS_BST
CNNNNNNNNANANNTTATTGCNTGTTCNNNTTGCCTAAGNTCACTATCANCTAGCAAATATTTCTTGTCAAAAATGCTCC
ACTGATGTTCCATAAATTCCCCTTGGTATCCAATTAGAGTCTCATATTCACTCTCAATCCAAATAATCTGCACTGGATCT
GGATTGTTTTGCATGATTGAACAAGATGGATTGCATGCAGGTTCTTTGGTTGTTTGGGTGGAGAGGTTATTTGGTTATGA
NTGGGCACAACANATAATTGGCTGCTCTGATGTCGTTGTGTTTNGGTTGTCANNGCANGGNTGCTTGGTTCTTTTTGTCA
AGATTGACCTGTCTGGNGCNCTGAATGAACTGCAGGATGAGGCANNGTGGCTATTGTGGCTGGNCATGATGGGTGTTCCT
TGCGCANCTGTGCTNGATGTTGTCACTGAANTGNGAAGGGACTGGCTGCTATTGGGCGAAGTGA

>PK_328409-504_2-R_Primer-B_D01.ab1
CNNNNNNNNNNNNNNNNTCNANCNCNNCTACNCAAAANACACCCATCATANCCAACCACAATAACCACACTACCTCATCCT
ACAATTCATTCAAAACACCAAACAAATCAATCTTAACAAAAAAAACCAAACACCCCTACACTAACAACCAAAACACAACA
ACATCAAAACAACCAATTATCTATTATGCCCAATCATAACCAAATAACCTCTCCACCCAAACAACCCAAAAAANTGCGNT
GCAANCCATCTTTGNNCNATCATGCAAAAAAACCCAAANCCNGGGGAAATTNTTTGGATNGNNAGNGGNANATGANNNNC
TAATTGGATACCAAGGGGAATTTNTGGNANNTCANGGGNNNNTTTTTGANNAGAAATATTTGNTAGCNGNNNNGGGNCTT
ANGGNANTTTTGNANACNCAAAANNNNNTTCNGANNNATGGGGNNNACTCNNTAAACNCCAAAAAACCCAN
>RC_328409-504_2-R_Primer-B_D01.ab1/>*ben1*_O_pNOS-BST
NTGGGTTTTTTGGNGTTTANNGAGTNNNCCCCATNNNTCNGAANNNNNTTTTGNGTNTNCAAAANTNCCNTAAGNCCCNNNNNCN
GCTANCAAATATTTCTNNTCAAAAANNNNCCCNTGANNTNCCANAAATTCCCCTTGGTATCCAATTAGNNNNTCATNTNCCNCTNN
CNATCCAAANAATTTCCCCNGGNTTTGGGTTTTTTTGCATGATNGNNCAAAGATGGNTTGCANCGCANTTTTTTGGGTTGTTTGGGT
GGAGAGGTTATTTGGTTATGATTGGGCATAATAGATAATTGGTTGTTTTGATGTTGTTGTGTTTTGGTTGTTAGTGTAGGGGTGTTTG
GTTTTTTTTGTTAAGATTGATTTGTTTGGTGTTTTGAATGAATTGTAGGATGAGGTAGTGTGGTTATTGTGGTTGGNTATGATGGGTG
TNTTTTGNGTAGNNGNGNTNGANNNNNNNNNNNNNNNNG

>PK_328409-505_3-F_Primer-A_E01.ab1/>*ben1*_R_pNOS_BST
NNNNNNNNNNNATTNTTATTNCGCGTTCAAAGTCGTCTAAGGTTACTATCAGTTAGNAAATATTTTTTGTCAAAAATGTTT
TACTGACGTTCCATAAATTTTTCTCGGTATTCAATTAGAGTCTCATATTTATTCTCAATTCAAATAATCTGTACCGGATC
TGGATCGTTTCGTATGATTGAACAAGATGGATTGTACGCANGTTTTCCGGTCGTTTGGGTGGAGAGGCTATTCGGCTATG
ACTGGGTACAACAGACAATCGGCTGTTCTGATGCCGCCGTGTTCCGGCTGTCAGTGCANGGGTGTCCGGTTTTTTTTGTC
AAGACCNATCTGTCCGGTGTTCTGAATGAACTGCANGACGAGGTANCGCGGCTATCGTGGCTGGTTACGANGGGNGTTTT
TTGCNTANCTGTGTTCGATGTTGTTACTGAAGCGGGAANGGACTGGCTGCTATTGGGCGAAGTGAN

>PK_328409-506_3-R_Primer-B_F01.ab1
NNNNNNNNNNNNNNNNCGANNCAGCTANCGCAANAACAGCCNGTCNGNACACCACGATAGCGCGCTANCNCGTCCTNGNAG
TTCATTCAGACACCGGACAGATCGGTCTTGACAANANNACCGGACACCCCTGCACTGACAGCCGNAACACGGCGGCATCA
GAACAGCCGATTGTCTGTTGTACCCAGTCATAGCNNNATANCCTCTCCACCCAANNNNNNNGGAAAACCTGCGTACAATCC
ATCTTGTTCAATCATACGAAACGATCCAGATCCGGTACAGATTATTTGAATTGAGANTGAATATGAGACTCTAATTGAAT
ACCGAGAAAAATTTATGNAACGTCANTNNACCATTTTTGACAAAAAATATTTGCTANCTGATAGTAACCTTAGACGACTT
TTGAACGCGCAATAATANTTTCTGACGTATGTACTTNNTCATTAAACTCCAAAANNNNNNNN

>RC_328409-506_3-R_Primer-B_F01.ab1/>*ben1*_R_pNOS-BST

NNNNNNNNNTTTTGGAGTTTAATGANNAAGTACATACGTCAGAAANTATTATTGCGCGTTCAAAAGTCGTCTAAGGTTACTATCAGN
TAGCAAATATTTTTTGTCAAAAATGGTNNANTGACGTTNCATAAATTTTTCTCGGTATTCAATTAGAGTCTCATATTCANTCTCAATTC
AAATAATCTGTACCGGATCTGGATCGTTTCGTATGATTGAACAAGATGGATTGTACGCAGGTTTTCCNNNNNNNTTGGGTGGAGAGG
NTATNNNGCTATGACTGGGTACAACAGACAATCGGCTGTTCTGATGCCGCCGTGTTNCGGCTGTCAGTGCAGGGGTGTCCGGTNNT
NTTGTCAAGACCGATCTGTCCGGTGTCTGAATGAACTNCNAGGACGNGNTAGCGCGCTATCGTGGTGTNCNGACNGGCTGTTNTTG
CGNTAGCTGNNTCGNNNNNNNNNNNNNNN

>PK_328409-507_4-F_Primer-A_G01.ab1/>*Triple*_pNOS-BST
NNNNNNNNNNNANNNTTATTGCGCGTTCAAAGTCGTCTAAGGTTACTATCAGCTAGCAAATATTTTTTGTCAAAAATGCT
CCACTGACGTTCCATAAATTTTTCTCGGTATTCAATTAGAGTCTCATATTCATTCTCAATTCAAATAATCTGTACCGGAT
CTGGATCGTTTCGTATGATTGAACAAGATGGATTGTACGCAGGTTCTCCGGCCGCTTGGGTGGAGAGGCTATTCGGCTAT
GACTGGGTACAACAGACAATCGGCTGTTCTGATGCCGCCGTGTTCCGGCTGTCAGCGCAGGGGCGTCCGGTTTTTTTTGT
CAAGACCGATCTGTCCGGTGTTCTGAATGAACTGCAGGACGAGGCAGCGCGGCTATCGTGGCTGGTTACGACGGGCGTTT
CTTGCGCAGCTGTGTTCGACGTTGTTACTGAAGCGGGAAGGGACTGGCTGCTATTGGGCGAAGTGA

>PK_328409-508_4-R_Primer-B_H01.ab1
NNNNNNNNNNNNNNNNNNNNNTCNANCACAGCTGCGCANAACGCCCGTCGTANCCAGNCCACGATAGCNCGCGCTGCCTCG
TCCTGCAGTTCATTCAGAACACCGGACAGATCGGTCTTGACAAAANNNNNCGGACGCCCCTGCGCTGACAGCCGGAACAC
GGCGGCATCAGAACAGCCGATTGTCTGTTGTACCCAGTCATAGCCGAATAGCCTCTCCACCCAAGCGNNNGGANAACCTG
CGTNNNATCCATCTTGTTCAATCATNCGAAACGATCCAGATCCGGTACAGATTATTTGAATTGAGANTGAATATGAGACT
CTAATTGAATACCGAGANAAATTTATGGAACGTCAGTGNAGCATTTTTGACAAAAAATATTTGCTAGCTGATAGTANCCT
TAGACGACTTTTGAACGCGCAATANTNNTTTCTGACGTATGTNCTTAACTCATTAAACTCCANAAACCCN

>RC_328409-508_4-R_Primer-B_H01.ab1/>*Triple*_pNOS-BST
NGGGTTTNTGGAGTTTAATGAGTTAAGNACATACGTCAGAAANNANTATTGCGCGTTCAAAAGTCGTCTAAGGNTACTATCAGCTA
GCAAATATTTTTTGTCAAAAATGCTNCACTGACGTTCCATAAATTTNTCTCGGTATTCAATTAGAGTCTCATATTCANTCTCAATTCAAA
TAATCTGTACCGGATCTGGATCGTTTCGNATGATTGAACAAGATGGATNNNACGCAGGTTNTCCNNNCGCTTGGGTGGAGAGGCTA
TTCGGCTATGACTGGGTACAACAGACAATCGGCTGTTCTGATGCCGCCGTGTTCCGGCTGTCAGCGCAGGGGCGTCCGNNNNNTTTT
GTCAAGACCGATCTGTCCGGTGTTCTGAATGAACTGCAGGACGAGGCAGCGCGNGCTATCGTGGNCTGGNTACGACGGGCGTTNTG
CGCAGCTGTGNTNGANNNNNNNNNNNNNNNNNNNN

**Sequence alignment:**

```
ben1_O_pNOS_no-BST    AAATATTTCTTGTCAAAAATGCTCCACTGACGTTCCATAAATTCCCCTCGGTATCCAATT
ben1_R_pNOS_BST       AAATATTTTTTGTCAAAAATGTTTACTGACGTTCCATAAATTTTTCTCGGTATTCAATT
ben1_O_pNOS_BST       AAATATTTCTTGTCAAAAATGCTCCACTGATGTTCCATAAATTCCCCTTGGTATCCAATT
                      ******** ************ *   ***** ************    ** ***** *****

ben1_O_pNOS_no-BST    AGAGTCTCATATTCACTCTCAATCCAAATAATCTGCACCGGATCTGGATCGTTTCGCATG
ben1_R_pNOS_BST       AGAGTCTCATATTTATTCTCAATTCAAATAATCTGTACCGGATCTGGATCGTTTCGTATG
ben1_O_pNOS_BST       AGAGTCTCATATTCACTCTCAATCCAAATAATCTGCACTGGATCTGGATTGTTTTGCATG
                      ************* * ******* *********** ** ********** **** * ***

ben1_O_pNOS_no-BST    ATTGAACAAGATGGATTGCACGCAGGTTCTCCGGCCGCTTGGGTGGAGAGGCTATTCGGC
ben1_R_pNOS_BST       ATTGAACAAGATGGATTGTACGCANGTTTTCCGGTCGTTTGGGTGGAGAGGCTATTCGGC
ben1_O_pNOS_BST       ATTGAACAAGATGGATTGCATGCAGGTTCTTTGGTTGTTTGGGTGGAGAGGTTATTTGGT
                      ****************** * *** *** *   **   * ************* **** **
```

```
ben1_O_pNOS_no-BST  TATGACTGGGCACAACAGACAATCGGCTGCTCTGATGCCGCCGTGTTC
ben1_R_pNOS_BST     TATGACTGGGTACAACAGACAATCGGCTGTTCTGATGCCGCCGTGTTC
ben1_O_pNOS_BST     TATGANTGGGCACAACANATAATTGGCTGCTCTGATGTCGTTGTGTTT
                    ***** **** ****** * *** ***** ******* **   *****
```

# Replicate 2

>PK_329142-501_2_pROK2-Fv_A04.ab1/>*ben1*_O_pNOS-BST
CCNNNNNNANANNTTATTGCGTGTTCNNNTTGCCTAAGGTCACTATCAGCTAGCAAATATTTCTTGTCAAAAATGCTCCA
CTGATGTTCCATAAATTCCCCTTGGTATCCAATTAGAGTCTCATATTCACTCTCAATCCAAATAATCTGCACTGGATCTG
GATTGTTTTGCATGATTGAACAAGATGGATTGCATGCAGGTTCTTTGGTTGTTTGGGTGGAGAGGTTATTTGGTTATGAT
TGGGTATAATAGATAATTGGTTGCTCTGATGTTGTTGTGTTTTGGTTGTCAGTGCAGGGGTGTTTGGTTCTTTTTGTCAA
GATTGATCTGTCTGGTGCTCTGAATGAACTGCAGGATGAGGCAGTGTGGCTATTGTGGCTGGTCATGATGGGTGTTCCTT
GCGCAGCTGTGCTTGATGTTGTCACTGAAGCGGGAAGGGACTGGCTGCTATTGGGCGAANNTGANCTCCCNCNAGACTGN
CCNANNNNNCNNCCTCCCNCTTTTNCTTGTGANCNNNNANCCANCNTNNCNNNNNNNNNCTNNNCTTCACTCNCNCNNNCT
CNNNCTTTGGTCTTCCCTCNCCCCNCTGCTCTCTCCCTCCCNN

>PK_329142-502_3_pROK2-Fv_B04.ab1/>*ben1*_R_pNOS-BST
NNNNNNNNNNNNNNTTATTGCGCGTTCAAAGTCGTCTAAGGTTACTATCAGTTANCAAATATTTTTTGTCAAAAATGTTNT
ACTGACGTTCCATAAATTTTTCTCGGTATTCAATTAGAGTCTCATATTTATTCTCAATTCAAATAATCTGTACCGGATCT
GGATCGTTTCGTATGATTGAACAAGATGGATTGTACGCAGGTTTTCCGGTCGTTTGGGTGGAGAGGCTATTCGGCTATGA
CTGGGTACAACAGACAATCGGCTGTTCTGATGCCGCCGTGTTCCGGCTGTCAGTGCAGGGGTGTTCGGTTTTTTTTGTCA
NNANCGATCTGTCCGGTGTTCTGAATGAACTGCAGGACGAGGCANCGCGGCTATCGTGGCTGGTTACGATGGGTGTTTTT
TGCGTANCTGTGTTCGATGTTGTTACTGAANCGGGAAGGGACTGGCTGCTATTGGGGCGAANTGANACNNCCAGNGNCCA
CACNCCTTNNTGCCNNCNNCCNNNTCTNNCNNATGACACCCAACANCNNCNNNCGNNTANNNATCNCCNCNNCTNNNCTC
CCGCNNACTCNTGCATAGNNGTCNNNNNNTCCTGACTGCN

>PK_329142-515_2_pROK2-Rv_A06.ab1/>*ben1*_O_pNOS-BST
NNNNNNNNNANNNNNNNNNNNNNCNNCTACNCAAAAGACACCCATCATAACCNACCACAATAACCACACTACCTCATCCTA
CAATTCATTCAAAACACCAAACAAATCAATCTTAACAAAAAAAACCAAACACCCCTACGCTAACAACCAAAACACAACAA
CATCAAAACAACCAATTATCTATTATACCCAATCGTAACCAAATAACCTCTCCACCCAAACAACCAANANAANNNNNGGN
NNNNCNNNTNNNANATAGNAAAAAGNNTNNAANCNGGGGNNANTATTTGGANNGAGAGNGGNATGAGANNTNNNTGGNNN
CCAGGGGAAATTTTGGGACNTTCAGGGGAGCTTTTTGGACAGAAATATTTGGTAACTGGNNANGGNCCTAAGGCAATTTT
GGACACNNAATAAAGGTTTCNGGNNNANGGGGCTNACCCCTTAAACCNANAAACCCAANNAGGNTGCGGGAGNNGNNGNN
GCACCAGNNGCTCGNTGAGAAAANGCNNNNGAGACTGGTACGATGTCGNAGNATCCGNNCCTGATCNTGCTGNGGGCTGN
NNTGCACTGTGTGNAGCNNNACGGCGACTAAATACTTGGTTTGNNATTGGANGTGCCGACGTCATGCTGGGGTGAANGTG
CCTGTG

>PK_329142-516_3_pROK2-Rv_B06.ab1/>*ben1*_R_pNOS-BST
NNNNNNNNNNNNNANNNNCNNNNNCNGCNNNNNNNNNNNNNCCCNNNCNNNAANNNNCNNNATAAGCCNGCGNTAGCNCGGN
CCTGNANNNNNTTCNNANCACCGGACNNATCGGCCTNGANNNNNNNNNACCGGANNCCCCTGCNCTGACAACCGGAACACGG
NGGCATCANANCAGNNGATNGTCTGTTGTACCNANNGNTAGCCNATTAACCTTCTCCCCCCCAANCGNCCCGGAAAACCT
GCGTACAATCCATCTTGTTCAATCATACGAAACGATCCAGATCCGGTACAGATTATTTGAATTGAGAGTGAATATNANAC
TCTAATTGAATACCGANAAAANNTNATGGAACGTCNGNGNAANATTTTTGNNAAAAAAATATTTGCTANCTGATAGTAAC
CTTAGACGACTTTTGAACGCGCAATANTAATTTCGGACGTATGTACTTAACTCATTAAACTCNNAAAAANNAAN
