# Supplementary material for: The ben1-1 Brassinosteroid-Catabolism Mutation Is Unstable Due to Epigenetic Modifications of the Intronic T-DNA Insertion
Source: G3 (Bethesda). 2013 Sep 1;3(9):1587–95. doi: 10.1534/g3.113.006353 (PMC3755919; doi:10.1534/g3.113.006353)
Supplement: Supporting Information [file supp_g3.113.006353_FileS4.pdf]

File S4

***NPTII* gene sequencing V2**

GTTTACCCGCCAATATATCCTGTCAAACACTGATAGTTTAAACTGAAGGCGGGAAACGACAATCTGATCATGAGCGGAGAATTAAGGGAGTCACGT
TATGACCCCCGCCGATGACGCGGGACAAGCCGTTTTACGTTTGGAACTGACAGAACCGCAACGTTGAAGGAGCCACTCAG**CCG/CGG**GTTTCTGGA
GTTTAATGAGCTAAGCACATACGTCAGAAACCATTATTGCGCGTTCAAAAGTCGCCTAAGGTCACTATCAGCTAGCAAATATTTCTTGTCAAAAATG
CTCCACTGACGTTCCATAAATTCCCCTCGGTATCCAATTAGAGTCTCATATTCACTCTCAATCCAAATAATCTGCACCGGATCTGGATCGTTTCGCATG
ATTGAACAAGATGGATTGCACGCAGGTTCTCCGGCCGCTTGGGTGGAGAGGCTATTCGGCTATGACTGGGCACAACAGACAATCGGCTGCTCTGA
TGCCGCCGTGTTCCGGCTGTCAGCGCAGGGGCGCCCGGTTCTTTTTGTCAAGACCGACCTGTCCGGTGCCCTGAATGAACTGCAGGACGAGGCAG
CGCGGCTATCGTGGCTGGCCACGACGGGCGTTCCTTGCGCAGCTGTGCTCGACGTTGTCACTGAAGCGGGAAGGGACTGGCTGCTATTGGGCGAA
GTGCCGGGGCAGGATCTCCTGTCATCTCACCTTGCTCCTGCCGAGAAAGTATCCATCATGGCTGATGCAATGCGGCGGCTGCATACGCTTGATCCG
GCTACCTGCCCATTCGACCACCAAGCGAAACATCGCATCGAGCGAGCACGTACTCGGATGGAAGCCGGTCTTGTCGATCAGGATGATCTGGACGA
AGAGCATCAGGGGCTCGCGCCAGCCGAACTGTTCGCCAGGCTCAAGGCGCGCATGCCCGACGGCGATGATCTCGTCGTGACCCATGGCGATGCCT
GCTTGCCGAATATCATGGTGGAAAATGGCCGCTTTTCTGGATTCATCGACTGTGGCCGGCTGGGTGTGGCGGACCGCTATCAGGACATAGCGTTG
GCTACCCGTGATATTGCTGAAGAGCTTGGCGGCGAATGGGCTGACCGCTTCCTCGTGCTTTACGGTATCGCCGCTCCCGATTCGCAGCGCATCGCCT
TCTATCGCCTTCTTGACGAGTTCTTCTGAGCGGGACTCTGGGGTTCGAAATGACCGACCAAGCGACGCCCAACCTGCCATCACGAGATTTCGATTCC
ACCGCCGCCTTCTATGAAAGGTTGGGCTTCGGAATCGTTTTCCGGGACGCCGGCTGGATGATCCTCCAGCGCGGGGATCTCATGCTGGAGTTCTTC
GCCCACGGGATCTCTGCGGAACA

>ben1_O_NPTII-F1
NNNNNNNACNTNNNNGAGCCACTCAGCCGCGGGTTTCTGGAGTTTAATGAGCTAAGCACATACGTCAGAAACCATTATTG
CGCGTTCAAAAGTCGCCTAAGGTCACTATCAGCTAGCAAATATTTCTTGTCAAAAATGCTCCACTGACGTTCCATAAATT
CCCCTCGGTATCCAATTAGAGTCTCATATTCACTCTCAATCCAAATAATCTGCACCGGATCTGGATCGTTTCGCATGATT
GAACAAGATGGATTGCACGCAGGTTCTCCGGCCGCTTGGGTGGAGAGGCTATTCGGCTATGACTGGGCACAACAGACAAT
CGGCTGCTCTGATGCCGCCGTGTTCCGGCTGTCAGCGCAGGGGCGCCCGGTTCTTTTTGTCAAGACCGACCTGTCCGGTG
CCCTGAATGAACTGCAGGACGAGGCAGCGCGGCTATCGTGGCTGGCCACGACGGGCGTTCCTTGCGCAGCTGTGCTCGAC
GTTGTCACTGAAGCGGGAAGGGACTGGCTGCTATTGGGCGAAGTGCCGGGGCAGGATCTCCTGTCATCTCACCTTGCTCC
TGCCGAGAAAGTATCCATCATGGCTGATGCAATGCGGCGGCTGCATACGCTTGATCCGGCTACCTGCCCATTCGACCACC
AAGCGAAACATCGCATCGAGCGAGCACGTACTCGGATGGAAGCCGGTCTTGTCGATCAGGATGATCTGGACGAAGAGCAT
CAGGGGCTCGCGCCAGCCGAACTGTTCGCCAGGCTCAAGGCGCGCATGCCCGACGGCGATGATCTCGTCGTGACCCATGG
CGATGCCTGCTTGCCGAATATCATGGTGGAAAATGGCCGCTTTTCTGGATTCATCGACTGTGGCCGGCTGGGTGTGGCGG
ACCGCTATCNNNCATAGCGTTGGCTACCCGTGATATTGCTGAAGAGCTTGGCGGCGAATGGGCTGACCGCTTCCTCGTGC
TTTACGGTATCGCCGCTCCCGATTCGCAGCGCATCNCCTTCTATCGCCTNCTTGACNAGTTCTTCTGANCGGNNNNNNNN
NNGGGGNNNNNNNNNNNNNNNNNAAAAAGNAN

>ben1_R_NPTII-F1
NNNNNNNACNTNNANGANCCNCTCAGCCGCGGGTTTCTGGAGTTTAATGAGCTAAGCACATACGTCAGAAACCATTATTG
CGCGTTCAAAAGTCGCCTAAGGTCACTATCAGCTAGCAAATATTTCTTGTCAAAAATGCTCCACTGACGTTCCATAAATT
CCCCTCGGTATCCAATTAGAGTCTCATATTCACTCTCAATCCAAATAATCTGCACCGGATCTGGATCGTTTCGCATGATT
GAACAAGATGGATTGCACGCAGGTTCTCCGGCCGCTTGGGTGGAGAGGCTATTCGGCTATGACTGGGCACAACAGACAAT
CGGCTGCTCTGATGCCGCCGTGTTCCGGCTGTCAGCGCAGGGGCGCCCGGTTCTTTTTGTCAAGACCGACCTGTCCGGTG
CCCTGAATGAACTGCAGGACGAGGCAGCGCGGCTATCGTGGCTGGCCACGACGGGCGTTCCTTGCGCAGCTGTGCTCGAC
GTTGTCACTGAAGCGGGAAGGGACTGGCTGCTATTGGGCGAAGTGCCGGGGCAGGATCTCCTGTCATCTCACCTTGCTCC
TGCCGAGAAAGTATCCATCATGGCTGATGCAATGCGGCGGCTGCATACGCTTGATCCGGCTACCTGCCCATTCGACCACC
AAGCGAAACATCGCATCGAGCGAGCACGTACTCGGATGGAAGCCGGTCTTGTCGATCAGGATGATCTGGACGAAGAGCAT
CAGGGGCTCGCGCCAGCCGAACTGTTCGCCAGGCTCAAGGCGCGCATGCCCGACGGCGATGATCTCGTCGTGACCCATGG
CGATGCCTGCTTGCCGAATATCATGGTGGAAAATGGCCGCTTTTCTGGATTCATCGACTGTGGCCGGCTGGGTGTGGCGG
ACCGCTATCNGACATAGCGTTGGCTACCCGTGATATTGCTGAAGAGCTTGGCGGCGAATGGGCTGACCGCTTCCTCGTGC
TTTACGGTATCGCCGCTCCCGATTCNCAGCGCATCGCCTTCTATCGCCTTCTTGACGANTTCTTCTGANCNGGNNNNNNN
NNNNNNGGNNNGNNNANNNNNAA

```
>ben1_O_NPTII-F2
NNNNNNNNNGNGGCGGNNNGCNNNNNNCNNNNNTCCGGCTNACCTNNCCCATTCGACCACCAAGCGAAACATCGCATCGAG
CGAGCACGTACTCGGATGGAAGCCGGTCTTGTCGATCAGGATGATCTGGACGAAGAGCATCAGGGGCTCGCGCCAGCCGA
ACTGTTCGCCAGGCTCAAGGCGCGCATGCCCGACGGCGATGATCTCGTCGTGACCCATGGCGATGCCTGCTTGCCGAATA
TCATGGTGGAAAATGGCCGCTTTTCTGGATTCATCGACTGTGGCCGGCTGGGTGTGGCGGACCGCTATCAGGACATAGCG
TTGGCTACCCGTGATATTGCTGAAGAGCTTGGCGGCGAATGGGCTGACCGCTTCCTCGTGCTTTACGGTATCGCCGCTCC
CGATTCGCAGCGCATCGCCTTCTATCGCCTTCTTGACGAGTTCTTCTGAGCGGGACTCTGGGGNNGAAATGAAAGAACCC
CCTCCNNACCTTCCCCCGGATGGGGGNCGAGGGAGCTGATCGCCANGNGGNNNCCANCNNNNANNACAANCCNCCGGCTT
GTGCTNNTTTTNNNCNNNNTTGTTGGTTATTTCNNANNNNTCNCNNNCCNNANNANTCATCNTNCTCACNNCACCNNNNN
NNCNNCNNNNTGTTGTTTTTNTTCTGATTGTTGATCANNNNNCTAANNAGCNNGGCTNNTTTTCTTNNNNCAGAGNNNAA
NNAAACACGCCNACNNCATNCNCNCCCNTTANTCNNTCTTCAGANACGANNTCANNNNNACAAACTNNNNNGCGCNANAN
ANANNATNTATCNCNNNACTCNNNNCTNGAAATANNNTACGANNNNNANANNAAAAANCANCCCCTCTTGCGTGGNTNTN
NACGAGAGTNNNTAGNNNNCNNNNGCGNAAAATNNNNNANACANNNANAGNNNNTGNNTTNATANNCGNCTANTNNTNGG
ANNNNTNNGTNATCNANNTNTNNANNNTNNNNTGTTCNCTNTGNGCCTGAATTTCNCTNTNNTCCCCCCNNNNNNTNNNN
NNCNNGGCTATCNGGCNNNTNTNTTTNTACNNTNTTGGAGGGAAGNNNNNNNNNNNNNNN

>ben1_R_NPTII-F2
NNNNNNNNNNGCGGNNNNNNNNNNNNNNNCNNNNNNNCCGNNTNACNTNNCCCATTTNCGACCACCAAGCGAAACATCGCATC
GAGCGAGCACGTACTCGGATGGAAGCCGGTCTNNGTCGATCAGGATGATCTGGACGAAGAGCATCAGGGGCTCGCGCCAG
CCGAACTGTTCGCCAGGCTCAAGGCGCGCATGCCCGACGGCGATGATCTCGTCGTGACCCATGGCGATGCCTGCTTGCCG
AATATCATGGTGGAAAATGGCCGCTTTTCTGGATTCATCGACTGTGGCCGGCTGGGTGTGGCGGACCGCTATCAGGACAT
AGCGTTGGCTACCCGTGATATTGCTGAAGAGCTTGGCGGCGAATGGGCTGACCGCTTCCTCGTGCTTTACGGTATCGCCG
CTCCCGATTCGCAGCGCATCGCCTTCTATCGCCTTCTTGACGAGTTCTTCTGAGCGGGACTCTGGGGNNCNAAATGAANN
CATCATCNNTCNNNNNGACNGGTGGNGGGACNGNGTGCAGTAGCNNAAGNCTAACAACTAACATGACCATCTCGNNGGAG
CTTTTTTCCACTTGAGAGACATTNCTTTTGGCTGTACNNCNTCCTNNNCCCTCCCATCATCTTTNNGCANCNCCNGCCAA
NTNTCNTNCNTCCTTTTGNTTCTNNTTNNTNNTCNNCANCNNNTGGGCTGGCCNGGNGTCTCTTGTNTACTANGNNGNNN
TGGACGGACCCGNNNNNNNGGGTTCCGNTTCNNTANAAAANCGTACCTNNNNGNNGCNNAGGGNNNNNGCCCCCAANNGG
NAAAAANTCTTCGTNCNTNNNCCCCGNANNAAGGNNNNATTGNNCNNAATANANANGACNGGCAAAANGGNGNNCNCNTN
NNNNGGNANTCNNNNNNNNGGAGGGNCGGNGNNGGGTCNNGGNNNNAACCNTNTTCNCAANNNNNNNAANNNNNTGNNNCNN
CCCNNNGNTNANTGNNTNGAANGANTTTTGCTNTCNNNCTGGGNNNNNATCTNNNTTNCNCTNNNGGTTTNTANNGNNNT
NNNNCCNCTCTCNCANNNNANTNNNNNAAGCNNNTANNNNGGGTTTTNNTTNNNNNTTTTTTNNNNNCGNNANNTNTNNN
TNNCNTNNNNNNNNNNNNNNNNTNNNNNGGGGGNNNTTTTNNGGNNAAAATGNNNANNNNNNNNNN
```

```
ben1_O_NPTII-F1   NNNNNNNACNTNNNNGAGCCACTCAGCCGCGGGTTTCTGGAGTTTAATGAGCTAAGCACA
ben1_R_NPTII-F1   NNNNNNNACNTNNANGANCCNCTCAGCCGCGGGTTTCTGGAGTTTAATGAGCTAAGCACA
                  ************* *** ** ***************************************

ben1_O_NPTII-F1   TACGTCAGAAACCATTATTGCGCGTTCAAAAGTCGCCTAAGGTCACTATCAGCTAGCAAA
ben1_R_NPTII-F1   TACGTCAGAAACCATTATTGCGCGTTCAAAAGTCGCCTAAGGTCACTATCAGCTAGCAAA
                  ************************************************************

ben1_O_NPTII-F1   TATTTCTTGTCAAAAATGCTCCACTGACGTTCCATAAATTCCCCTCGGTATCCAATTAGA
ben1_R_NPTII-F1   TATTTCTTGTCAAAAATGCTCCACTGACGTTCCATAAATTCCCCTCGGTATCCAATTAGA
                  ************************************************************

ben1_O_NPTII-F1   GTCTCATATTCACTCTCAATCCAAATAATCTGCACCGGATCTGGATCGTTTCGCATGATT
ben1_R_NPTII-F1   GTCTCATATTCACTCTCAATCCAAATAATCTGCACCGGATCTGGATCGTTTCGCATGATT
                  ************************************************************

ben1_O_NPTII-F1   GAACAAGATGGATTGCACGCAGGTTCTCCGGCCGCTTGGGTGGAGAGGCTATTCGGCTAT
ben1_R_NPTII-F1   GAACAAGATGGATTGCACGCAGGTTCTCCGGCCGCTTGGGTGGAGAGGCTATTCGGCTAT
                  ************************************************************
```

```
ben1_O_NPTII-F1  GACTGGGCACAACAGACAATCGGCTGCTCTGATGCCGCCGTGTTCCGGCTGTCAGCGCAG
ben1_R_NPTII-F1  GACTGGGCACAACAGACAATCGGCTGCTCTGATGCCGCCGTGTTCCGGCTGTCAGCGCAG
                 ************************************************************

ben1_O_NPTII-F1  GGGCGCCCGGTTCTTTTTGTCAAGACCGACCTGTCCGGTGCCCTGAATGAACTGCAGGAC
ben1_R_NPTII-F1  GGGCGCCCGGTTCTTTTTGTCAAGACCGACCTGTCCGGTGCCCTGAATGAACTGCAGGAC
                 ************************************************************

ben1_O_NPTII-F1  GAGGCAGCGCGGCTATCGTGGCTGGCCACGACGGGCGTTCCTTGCGCAGCTGTGCTCGAC
ben1_R_NPTII-F1  GAGGCAGCGCGGCTATCGTGGCTGGCCACGACGGGCGTTCCTTGCGCAGCTGTGCTCGAC
                 ************************************************************

ben1_O_NPTII-F1  GTTGTCACTGAAGCGGGAAGGGACTGGCTGCTATTGGGCGAAGTGCCGGGGCAGGATCTC
ben1_R_NPTII-F1  GTTGTCACTGAAGCGGGAAGGGACTGGCTGCTATTGGGCGAAGTGCCGGGGCAGGATCTC
                 ************************************************************

ben1_O_NPTII-F1  CTGTCATCTCACCTTGCTCCTGCCGAGAAAGTATCCATCATGGCTGATGCAATGCGGCGG
ben1_R_NPTII-F1  CTGTCATCTCACCTTGCTCCTGCCGAGAAAGTATCCATCATGGCTGATGCAATGCGGCGG
                 ************************************************************

ben1_O_NPTII-F1  CTGCATACGCTTGATCCGGCTACCTGCCCATTCGACCACCAAGCGAAACATCGCATCGAG
ben1_R_NPTII-F1  CTGCATACGCTTGATCCGGCTACCTGCCCATTCGACCACCAAGCGAAACATCGCATCGAG
                 ************************************************************

ben1_O_NPTII-F1  CGAGCACGTACTCGGATGGAAGCCGGTCTTGTCGATCAGGATGATCTGGACGAAGAGCAT
ben1_R_NPTII-F1  CGAGCACGTACTCGGATGGAAGCCGGTCTTGTCGATCAGGATGATCTGGACGAAGAGCAT
                 ************************************************************

ben1_O_NPTII-F1  CAGGGGCTCGCGCCAGCCGAACTGTTCGCCAGGCTCAAGGCGCGCATGCCCGACGGCGAT
ben1_R_NPTII-F1  CAGGGGCTCGCGCCAGCCGAACTGTTCGCCAGGCTCAAGGCGCGCATGCCCGACGGCGAT
                 ************************************************************

ben1_O_NPTII-F1  GATCTCGTCGTGACCCATGGCGATGCCTGCTTGCCGAATATCATGGTGGAAAATGGCCGC
ben1_R_NPTII-F1  GATCTCGTCGTGACCCATGGCGATGCCTGCTTGCCGAATATCATGGTGGAAAATGGCCGC
                 ************************************************************

ben1_O_NPTII-F1  TTTTCTGGATTCATCGACTGTGGCCGGCTGGGTGTGGCGGACCGCTATCNNNCATAGCGT
ben1_R_NPTII-F1  TTTTCTGGATTCATCGACTGTGGCCGGCTGGGTGTGGCGGACCGCTATCNGACATAGCGT
                 **************************************************  ********

ben1_O_NPTII-F1  TGGCTACCCGTGATATTGCTGAAGAGCTTGGCGGCGAATGGGCTGACCGCTTCCTCGTGC
ben1_R_NPTII-F1  TGGCTACCCGTGATATTGCTGAAGAGCTTGGCGGCGAATGGGCTGACCGCTTCCTCGTGC
                 ************************************************************

ben1_O_NPTII-F1  TTTACGGTATCGCCGCTCCCGATTCGCAGCGCATCNCCTTCTATCGCCTNCTTGACNAGT
ben1_R_NPTII-F1  TTTACGGTATCGCCGCTCCCGATTCNCAGCGCATCGCCTTCTATCGCCTTCTTGACGANT
                 ************************* ********* ************* ****** * *

ben1_O_NPTII-F1  TCTTCTGANCGGNNNNNNNNNNNGGGNNNNNNNNNNNNNNNNNNAAAAAGNAN
ben1_R_NPTII-F1  TCTTCTGANCNGGNNNNNNNNNNNNNNNGGNNNGNNNANNNNNNAA--------
                 ********** * *********       *** *** ***** *


ben1_O_NPTII-F2  -NNNNNNNNGNGGCGGNNNGCNNNNNNCNNNNNTCCGGCTNACCTNNCCCATT--CGACC
ben1_R_NPTII-F2  NNNNNNNNNGCGGNNNNNNNNNNNNNNNCNNNNNNCCGNNTNACNTNNCCCATTTNCGACC
                  ********* **    ***   ************ ***   **** *********   *****
```

```
ben1_O_NPTII-F2   ACCAAGCGAAACATCGCATCGAGCGAGCACGTACTCGGATGGAAGCCGGTCTTG-TCGAT
ben1_R_NPTII-F2   ACCAAGCGAAACATCGCATCGAGCGAGCACGTACTCGGATGGAAGCCGGTCTNNGTCGAT
                  ****************************************************   *****

ben1_O_NPTII-F2   CAGGATGATCTGGACGAAGAGCATCAGGGGCTCGCGCCAGCCGAACTGTTCGCCAGGCTC
ben1_R_NPTII-F2   CAGGATGATCTGGACGAAGAGCATCAGGGGCTCGCGCCAGCCGAACTGTTCGCCAGGCTC
                  ************************************************************

ben1_O_NPTII-F2   AAGGCGCGCATGCCCGACGGCGATGATCTCGTCGTGACCCATGGCGATGCCTGCTTGCCG
ben1_R_NPTII-F2   AAGGCGCGCATGCCCGACGGCGATGATCTCGTCGTGACCCATGGCGATGCCTGCTTGCCG
                  ************************************************************

ben1_O_NPTII-F2   AATATCATGGTGGAAAATGGCCGCTTTTCTGGATTCATCGACTGTGGCCGGCTGGGTGTG
ben1_R_NPTII-F2   AATATCATGGTGGAAAATGGCCGCTTTTCTGGATTCATCGACTGTGGCCGGCTGGGTGTG
                  ************************************************************

ben1_O_NPTII-F2   GCGGACCGCTATCAGGACATAGCGTTGGCTACCCGTGATATTGCTGAAGAGCTTGGCGGC
ben1_R_NPTII-F2   GCGGACCGCTATCAGGACATAGCGTTGGCTACCCGTGATATTGCTGAAGAGCTTGGCGGC
                  ************************************************************

ben1_O_NPTII-F2   GAATGGGCTGACCGCTTCCTCGTGCTTTACGGTATCGCCGCTCCCGATTCGCAGCGCATC
ben1_R_NPTII-F2   GAATGGGCTGACCGCTTCCTCGTGCTTTACGGTATCGCCGCTCCCGATTCGCAGCGCATC
                  ************************************************************

ben1_O_NPTII-F2   GCCTTCTATCGCCTTCTTGACGAGTTCTTCTGAGCGGGACTCTGGGGNN-GAAATGAAAG
ben1_R_NPTII-F2   GCCTTCTATCGCCTTCTTGACGAGTTCTTCTGAGCGGGACTCTGGGGNNCNAAATGAANN
                  *************************************************  *******
```
