# Supplementary material for: The ben1-1 Brassinosteroid-Catabolism Mutation Is Unstable Due to Epigenetic Modifications of the Intronic T-DNA Insertion
Source: G3 (Bethesda). 2013 Sep 1;3(9):1587–95. doi: 10.1534/g3.113.006353 (PMC3755919; doi:10.1534/g3.113.006353)
Supplement: Supporting Information [file supp_g3.113.006353_TableS1.pdf]

Table S1 Table of PCR primer sequences used in the study.

Primer Name	Primer Sequences
GSP1	5'-TTTATATCTTCGTCGTTGCCT-3'
GSP2	5'-ACCTTTTGCAACTGGCTTTT-3'
LBb1.3	5'-ATTTTGCCGATTTCGGAAC-3'
PRT1	5'-ATGGTGAGAGAAGAACAAGA-3'
PRT2	5'-TTAAAGAAATCCCCTTGCTTG-3'
PQ1	5'-ATCTCTCGCCATGCTCTTTG-3'
PQ2	5'-CTTCATCTCCACCGACGAAC-3'
UBQ1	5'-GAAATGCATGGAGACGGATT-3'
UBQ2	5'-TTGGTCTCTGCTCCCACTCT-3'
ACTIN2 RT-F	5'-GGTCGTACAACCGGTATTGTGCTGG-3'
ACTIN2 RT-R	5'-CTGTGAACGATTCCTGGACCTGCC-3'
PQ3	5'-TCTAAACAAGGAATTCGAGGTC-3'
PQ4	5'-AACCAGAAATGTAGCACCGT-3'
NPT-Fv1/MT1	5'-ACAAGCCGTTTTACGTTTGG-3'
NPT-Rv	5'-TCATTTCGAACCCCAGAGTC-3'
MT2	5'-ATACTTTCTCGGCAGGAGCA-3'
MT3	5'-TTCGCAAGACCCTTCCTCTA-3'
MT4	5'-GTTTTCCCAGTCACGACGTT-3'
NPT-Fv2	5'-TGCTCCTGCCGAGAAAGTAT-3'
pNOS-BS-F	5'-GGGTTTYTGGAGTTTAATGAGYTAAG-3'
pNOS-BS-R	5'-CACTTCRCCCAATARCARCCARTCCCTTCC-3'
pBEN1-BS-F	5'-GTATAGTAGTAAAAAAAAGAAGAAGAAG-3'
pBEN1-BS-R	5'-CTTRTTCTTCTCTCACCATTTTCTCTCT-3'
BEN1-E2-BS-F	5'-TTAGGAATTAAAAGGAGAAGAG-3'
BEN1-E2-BS-R	5'-AATTAAATACCAAAAAACATAAC-3'
